# Supplementary material for: A transcriptional and regulatory map of mouse somite maturation
Source: Dev Cell. 2023 Oct 9;58(19):1983–1995.e7. doi: 10.1016/j.devcel.2023.07.003 (PMC10563765; doi:10.1016/j.devcel.2023.07.003)

Developmental Cell, Volume *58*

Supplemental information

A transcriptional and regulatory map of mouse somite maturation

Ximena Ibarra-Soria, Elodie Thierion, Gi Fay Mok, Andrea E. Münsterberg, Duncan T. Odom, and John C. Marioni

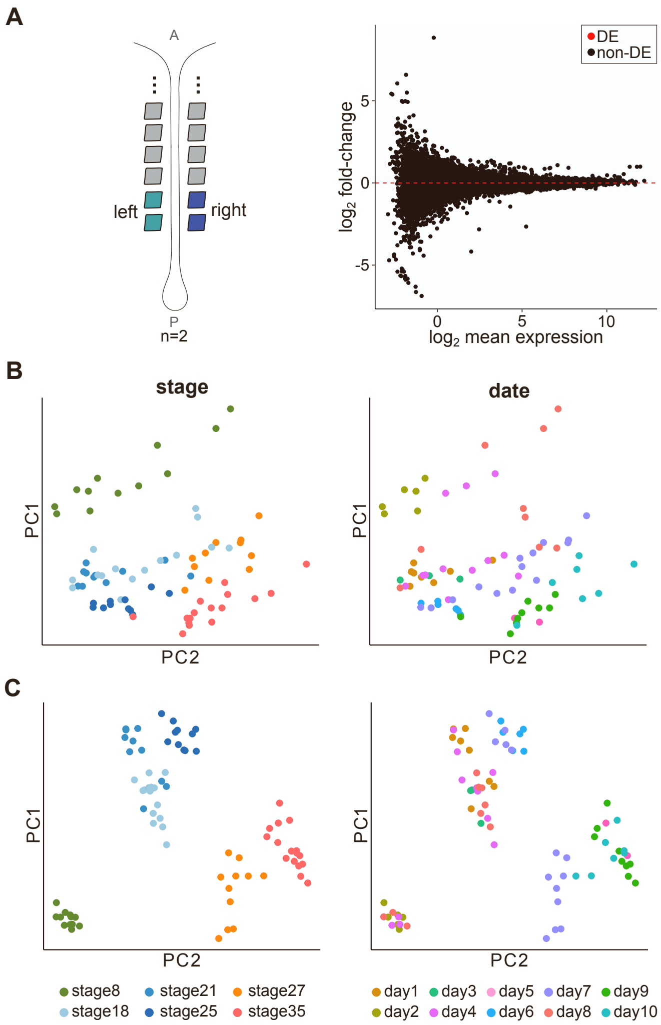

Figure S1 | The left and right somites are transcriptionally equivalent, related to STAR Methods. A) We compared the transcriptomes of matched left and right somites by RNA-seq using the two most posterior somite pairs, as shown in the schematic. A: anterior; P: posterior. The scatter plot shows the average gene expression level on the x-axis and the corresponding log_2 fold-change between the left and right somites on the y-axis. No significantly differentially expressed (DE) genes were identified (FDR 5%), indicating that the transcriptomes of the two somites from the same pair are equivalent. **B)** PCA of the normalised counts of the thousand most variable genes across samples. There is clear separation by developmental stage (left). However, PC1 also separates samples based on their collection day (right). **C)** PCA after regressing out covariates capturing variation unrelated to the experimental design. Samples separate better by their developmental stage (left) and grouping by collection day is no longer evident (right).

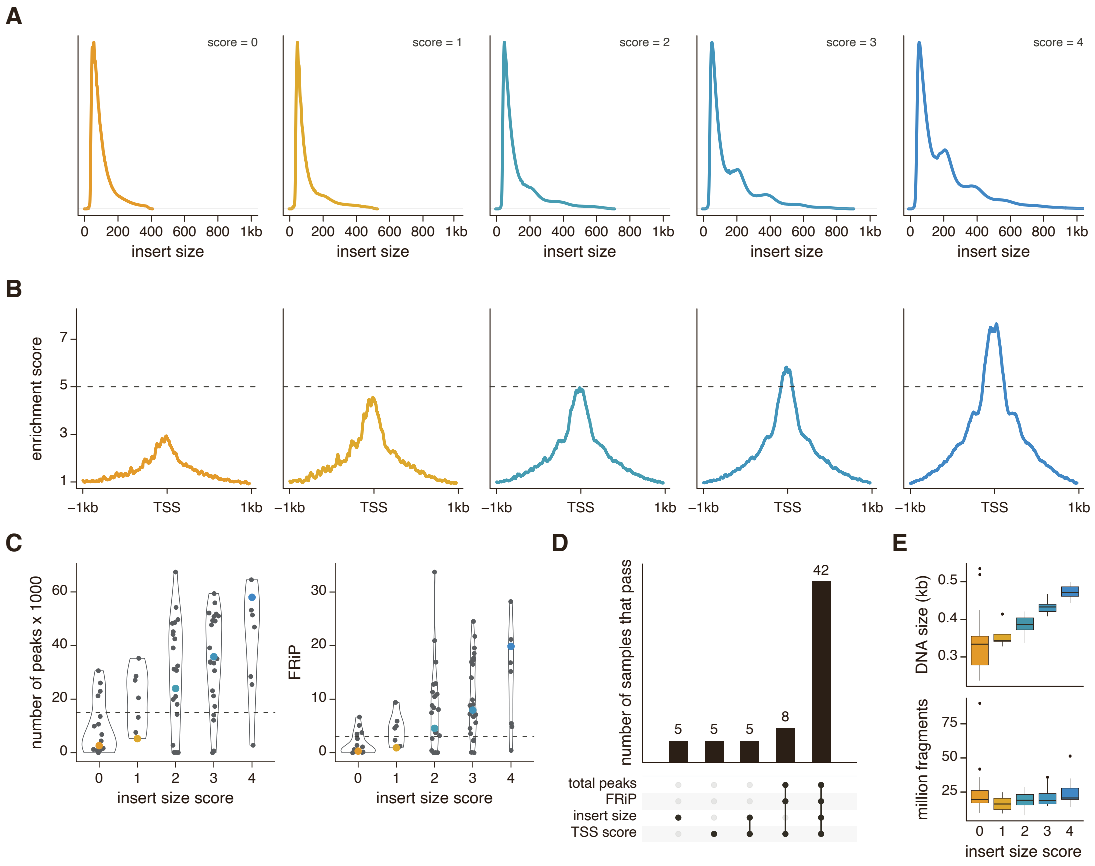

Figure S2 | Quality control of ATAC-seq libraries, related to STAR Methods.

Several metrics were used to determine if the ATAC-seq libraries were of good quality. **A)** Representative density plots of the fragment sizes of the sequencing data for each score group. Scores were determined based on the number of nucleosomal peaks. Colours indicate different score groups, with yellow (blue) shades assigned to poor (good) quality samples. **B)** Representative plots of the cumulative signal for 2 kb intervals centred at the transcription start site (TSS) of expressed genes, for the same samples shown in A. An enrichment score larger than 1 indicates an excess of insertions relative to background. Signal is smoothed by taking the rolling median of 25 bp intervals. **C)** Violin plots depicting the number of total peaks called from each library (left) and the fraction of reads in peaks (FRiP; right), stratified by the sample's insert size distribution score. The samples depicted in A-B are highlighted by coloured points. **D)** Number of samples that pass each of the QC criteria. The 50 samples that passed three or four criteria were deemed of good enough quality for downstream analyses. Samples that failed all four criteria are not shown. **E)** Boxplots of the experimentally determined DNA fragment size (top) and the library size of the sequenced samples (bottom), stratified by the insert size distribution score. No relationship is observed between library size and size distribution score, indicating that the poor-quality samples are not due to insufficient sequencing.

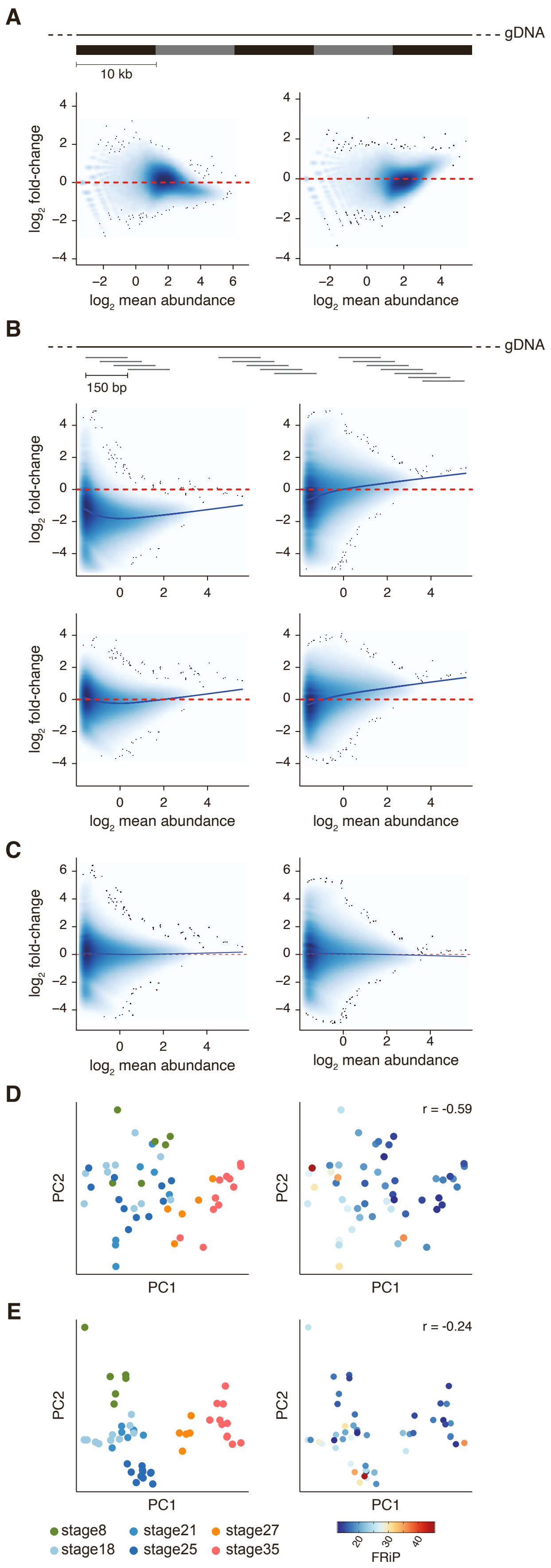

Figure S3 | Normalisation of ATAC-seq data, related to STAR Methods.

A) MA plots of two representative samples. On the x-axis is the $\log_2$ average number of sequencing fragments in 10kb bins covering the mouse genome. The y-axis corresponds to the $\log_2$ fold-change against a reference sample (same for both panels). Comparable samples should show log fold-changes centred around 0. High abundance bins (which contain open regions) show significant deviation from 0. This deviation shows a trend dependent on mean abundance. **B)** To normalise the observed biases we focused on the regions of open chromatin. The MA plots now show on the x-axis the average counts in 150bp windows that slide 50bp, restricted to regions overlapping called peaks. At the top, the raw counts for the same samples in A. At the bottom, scaling normalisation is applied, which results in a shift towards a fold-change of 0. However, the observed trend dependent on mean abundance is still present in the data. **C)** MA plots for the same samples in B, but after applying loess-based normalisation, which computes a size factor for each abundance level. This successfully captured and removed the observed trend, with windows now centred around 0. **D)** PCA of the normalised counts of the 5000 most variable windows. On the left, samples are coloured by developmental stage while on the right they are coloured by their fraction of reads in peaks (FRiP). There is clear grouping of samples based on their FRiP. The Pearson correlation coefficient between FRiP and PC1 is noted. **E)** PCA after regressing out covariates capturing variation unrelated to the experimental design. Samples separate better by their developmental stage (left) and there is no longer a significant correlation with FRiP (right).

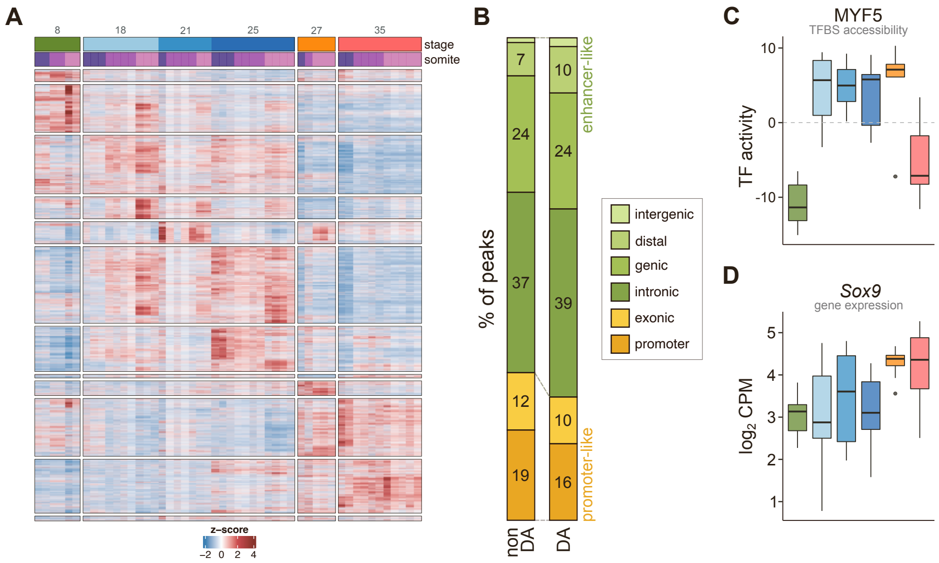

Figure S4 | Differentially accessible chromatin loci across development, related to Figure 3. **A)** Similar to Figure 3B but showing the accessibility levels of all differential peaks across development. Samples (columns) are ordered based on their observed somite number, and their stage and somite level are indicated at the top. Peaks (rows) are split into clusters by hierarchical clustering. **B)** Same as Figure 2E but for differentially accessible regions across development. **C)** Chromatin activity scores from chromVAR for the genome-wide binding sites (TFBS) of MYF5 across development. **D)** Gene expression levels for *Sox9* across development.

						RNA-seq				ATAC-seq			
sampleName	embryo	somite	somiteNumber	somiteStage	collectionDate	RNA_ID	RNA_QC	size (bp)	concentration [nM]	ATAC_ID	ATAC_QC	size (bp)	concentration [nM]
e5_SI	e5	SI	8	8	06.04.18	do26267	1	474	2.42	do26190	1	486	5.78
e5_SII	e5	SII	7	8	06.04.18	do26268	1	442	3.56	do26191	0	425	9.5
e5_SIII	e5	SIII	6	8	06.04.18	do26269	1	439	3	do26192	0	469	10.76
e6_SI	e6	SI	8	8	06.04.18	do26270	1	408	2.76	do26193	0	425	5.86
e6_SII	e6	SII	7	8	06.04.18	do26271	1	403	1.88	do26194	0	439	7.1
e6_SIII	e6	SIII	6	8	06.04.18	do26272	1	390	1.5	NA	NA	NA	NA
e16_SI	e16	SI	8	8	26.04.18	do26285	1	445	2.4	do26205	0	341	6.26
e16_SII	e16	SII	7	8	26.04.18	do26286	1	451	3.1	do26206	1	342	7.88
e16_SIII	e16	SIII	6	8	26.04.18	do26287	1	440	2.9	do26207	1	420	6.08
e26_SI	e26	SI	8	8	01.06.18	do26314	1	470	8.2	do26233	1	437	7.74
e26_SII	e26	SII	7	8	01.06.18	do26315	1	527	12.88	do26234	1	372	5.04
e26_SIII	e26	SIII	6	8	01.06.18	do26316	1	518	8.36	do26235	1	444	8.12
e9_SI	e9	SI	18	18	12.04.18	do26273	1	484	2.36	do26195	1	423	6.84
e9_SII	e9	SII	17	18	12.04.18	do26274	1	416	3.04	do26196	0	414	9.7
e9_SIII	e9	SIII	16	18	12.04.18	do26275	1	438	4.98	do26197	1	437	7.46
e14_SI	e14	SI	18	18	26.04.18	do26279	1	500	6.16	NA	NA	NA	NA
e14_SII	e14	SII	17	18	26.04.18	do26280	1	474	5.06	do26201	1	473	7.72
e14_SIII	e14	SIII	16	18	26.04.18	do26281	1	467	3.98	do26202	0	425	7.56
e15_SI	e15	SI	18	18	26.04.18	do26282	1	474	3.18	do26203	1	393	6.8
e15_SII	e15	SII	17	18	26.04.18	do26283	1	497	2.9	do26204	1	447	6.64
e15_SIII	e15	SIII	16	18	26.04.18	do26284	1	449	3.8	NA	NA	NA	NA
e27_SI	e27	SI	18	18	01.06.18	do26317	1	489	13.08	do26236	1	380	7.66
e27_SII	e27	SII	17	18	01.06.18	do26318	1	446	6.7	do26237	1	364	9.04
e27_SIII	e27	SIII	16	18	01.06.18	do26319	1	378	1.91	do26238	1	348	14.8
e28_SI	e28	SI	18	18	01.06.18	do26320	1	424	14.2	do26239	0	303	17.8
e28_SII	e28	SII	17	18	01.06.18	do26321	1	435	8.02	do26240	1	334	10.1
e28_SIII	e28	SIII	16	18	01.06.18	do26322	1	484	2.52	do26241	1	390	5.97
e1_SI	e1	SI	21	21	05.04.18	do26259	1	479	5.66	do26181	1	328	18
e1_SII	e1	SII	20	21	05.04.18	do26260	1	522	4.32	do26182	1	368	18.6
e1_SIII	e1	SIII	19	21	05.04.18	do26261	1	477	3.76	do26183	0	344	18.1
e2_SI	e2	SI	21	21	05.04.18	do26262	1	469	4.46	do26184	0	396	8.87
e2_SII	e2	SII	20	21	05.04.18	NA	NA	NA	NA	do26185	1	421	6.42
e2_SIII	e2	SIII	19	21	05.04.18	do26263	1	412	3.68	do26186	0	337	12.1
e3_SI	e3	SI	21	21	05.04.18	do26264	1	466	8.98	do26187	0	376	14.9
e3_SII	e3	SII	20	21	05.04.18	do26265	1	488	7.88	do26188	1	409	8.44
e3_SIII	e3	SIII	19	21	05.04.18	do26266	1	514	9.92	do26189	0	414	11.2
e13_SI	e13	SI	21	21	26.04.18	do26276	1	469	9.62	do26198	1	441	7.38
e13_SII	e13	SII	20	21	26.04.18	do26277	1	500	2.4	do26199	1	489	6.22
e13_SIII	e13	SIII	19	21	26.04.18	do26278	1	496	3.76	do26200	1	447	6.76
e19_SI	e19	SI	25	25	20.05.18	do26294	1	504	9.12	do26214	1	421	11.4
e19_SII	e19	SII	24	25	20.05.18	do26295	1	501	6.7	do26215	1	408	6.19
e19_SIII	e19	SIII	23	25	20.05.18	do26296	1	518	9.94	do26216	0	415	8.24
e20_SI	e20	SI	25	25	20.05.18	do26297	1	446	3.9	do26217	1	468	5.3
e20_SII	e20	SII	24	25	20.05.18	do26298	1	415	7.82	do26218	1	419	9.44
e20_SIII	e20	SIII	23	25	20.05.18	do26299	1	422	3	do26219	1	441	6.75
e21_SI	e21	SI	25	25	24.05.18	do26300	1	465	11.9	do26220	1	404	8.26
e21_SII	e21	SII	24	25	24.05.18	NA	NA	NA	NA	do26221	1	461	7.58
e21_SIII	e21	SIII	23	25	24.05.18	do26301	1	586	6.98	do26222	1	438	7.32
e22_SI	e22	SI	25	25	24.05.18	do26302	1	508	7.8	do26223	1	433	5.7
e22_SII	e22	SII	24	25	24.05.18	do26303	1	557	8.86	do26224	1	421	7.34
e22_SIII	e22	SIII	23	25	24.05.18	do26304	1	520	9.14	do26225	1	407	8.58
e23_SI	e23	SI	27	27	24.05.18	do26305	1	590	26.8	do26226	1	237	5.88
e23_SII	e23	SII	26	27	24.05.18	do26306	1	510	8.82	do26227	0	280	32.2
e23_SIII	e23	SIII	25	27	24.05.18	do26307	1	385	1.6	do26228	0	372	8.05
e24_SI	e24	SI	27	27	24.05.18	do26308	1	473	12.98	do26229	1	343	14.8
e24_SII	e24	SII	26	27	24.05.18	do26309	1	437	8.88	do26230	1	405	6.22
e24_SIII	e24	SIII	25	27	24.05.18	do26310	1	391	13.3	do26231	1	361	8.52
e25_SI	e25	SI	27	27	24.05.18	do26311	1	486	14.8	do26232	1	380	9.48
e25_SII	e25	SII	26	27	24.05.18	do26312	1	546	14.12	NA	NA	NA	NA
e25_SIII	e25	SIII	25	27	24.05.18	do26313	1	576	16	NA	NA	NA	NA
e34_SI	e34	SI	27	27	14.06.18	do26337	1	450	16.66	do26256	0	356	15.7
e34_SII	e34	SII	26	27	14.06.18	do26338	1	476	17.52	do26257	0	274	14.3
e34_SIII	e34	SIII	25	27	14.06.18	NA	NA	NA	NA	do26258	0	324	30
e17_SI	e17	SI	35	35	27.04.18	do26288	1	507	15.8	do26208	1	466	7.94
e17_SII	e17	SII	34	35	27.04.18	do26289	0	528	12.2	do26209	1	449	7.52
e17_SIII	e17	SIII	33	35	27.04.18	do26290	1	543	7.82	do26210	1	382	9.8
e29_SI	e29	SI	35	35	11.06.18	do26323	1	468	13.76	do26242	0	535	10.86
e29_SII	e29	SII	34	35	11.06.18	do26324	1	470	12.02	do26243	1	372	15
e29_SIII	e29	SIII	33	35	11.06.18	do26325	1	528	9.04	do26244	0	360	19.2
e30_SI	e30	SI	35	35	11.06.18	do26326	1	489	14.42	do26245	1	386	10.3
e30_SII	e30	SII	34	35	11.06.18	do26327	1	497	12.5	do26246	1	393	12.5
e30_SIII	e30	SIII	33	35	11.06.18	do26328	1	492	4.94	NA	NA	NA	NA
e31_SI	e31	SI	35	35	11.06.18	do26329	1	506	7.46	do26247	1	262	39
e31_SII	e31	SII	34	35	11.06.18	do26330	1	469	10.14	do26248	0	269	20.2
e31_SIII	e31	SIII	33	35	11.06.18	do26331	1	496	7.1	do26249	0	334	37.1
e32_SI	e32	SI	35	35	14.06.18	do26332	1	468	12.3	do26250	1	419	6.73
e32_SII	e32	SII	34	35	14.06.18	NA	NA	NA	NA	do26251	1	436	6.36
e32_SIII	e32	SIII	33	35	14.06.18	do26333	1	428	2.52	do26252	1	500	6.92
e33_SI	e33	SI	35	35	14.06.18	do26334	1	453	21.6	do26253	1	424	10.76
e33_SII	e33	SII	34	35	14.06.18	do26335	1	448	7.42	do26254	0	355	5.82
e33_SIII	e33	SIII	33	35	14.06.18	do26336	1	476	11.84	do26255	0	519	7.78

Table S1 | Metadata of the samples collected and the RNA- and ATAC-seq libraries produced, related to STAR Methods.
The QC columns indicate whether the sample passed quality control (1) or not (0; highlighted in red); NA indicates that the sample did not yield a successful library for sequencing.

RNA-seq																				
sampleName	embryo	somite	somiteNumber	somiteStage	collectionDate	RNA_ID	RNA_QC	size (bp)	concentration [nM]	totalFragments	uniquelyMapped		multimapped		unmapped		uniqueInExons		numberGenes	QCpass
e5_SI	e5	SI	8	8	06.04.18	do26267	1	474	2.42	11,104,305	9,173,394	82.61%	732,698	6.60%	1,198,213	10.79%	7811293	85.15%	19,997	1
e5_SII	e5	SII	7	8	06.04.18	do26268	1	442	3.56	13,116,915	11,025,523	84.06%	835,470	6.37%	1,255,922	9.57%	9471526	85.91%	20,042	1
e5_SIII	e5	SIII	6	8	06.04.18	do26269	1	439	3	15,002,486	12,870,669	85.79%	972,766	6.48%	1,159,051	7.73%	11039612	85.77%	19,860	1
e6_SI	e6	SI	8	8	06.04.18	do26270	1	408	2.76	28,218,690	20,787,050	73.66%	2,052,009	7.27%	5,379,631	19.06%	16323073	78.53%	22,343	1
e6_SII	e6	SII	7	8	06.04.18	do26271	1	403	1.88	28,976,459	21,475,857	74.11%	2,018,767	6.97%	5,481,835	18.92%	16985675	79.09%	22,074	1
e6_SIII	e6	SIII	6	8	06.04.18	do26272	1	390	1.5	26,472,304	17,277,583	65.27%	2,092,029	7.90%	7,102,692	26.83%	13215689	76.49%	22,272	1
e16_SI	e16	SI	8	8	26.04.18	do26285	1	445	2.4	11,679,604	9,127,484	78.15%	783,202	6.71%	1,768,918	15.15%	7778120	85.22%	19,549	1
e16_SII	e16	SII	7	8	26.04.18	do26286	1	451	3.1	30,925,538	24,211,119	78.29%	2,204,497	7.13%	4,509,922	14.58%	20816820	85.98%	21,462	1
e16_SIII	e16	SIII	6	8	26.04.18	do26287	1	440	2.9	24,721,994	19,018,399	76.93%	1,775,099	7.18%	3,928,496	15.89%	16093472	84.62%	22,029	1
e26_SI	e26	SI	8	8	01.06.18	do26314	1	470	8.2	17,096,838	14,592,409	85.35%	1,074,804	6.29%	1,429,625	8.36%	12666068	86.80%	22,087	1
e26_SII	e26	SII	7	8	01.06.18	do26315	1	527	12.88	19,270,385	17,269,913	89.62%	1,071,621	5.56%	928,851	4.82%	14700672	85.12%	24,105	1
e26_SIII	e26	SIII	6	8	01.06.18	do26316	1	518	8.36	14,645,123	13,137,082	89.70%	797,987	5.45%	710,054	4.85%	11294665	85.98%	21,659	1
e9_SI	e9	SI	18	18	12.04.18	do26273	1	484	2.36	15,593,104	12,579,677	80.67%	1,007,566	6.46%	2,005,861	12.86%	10713991	85.17%	20,588	1
e9_SII	e9	SII	17	18	12.04.18	do26274	1	416	3.04	31,478,723	24,015,046	76.29%	2,163,684	6.87%	5,299,993	16.84%	20124042	83.80%	22,634	1
e9_SIII	e9	SIII	16	18	12.04.18	do26275	1	438	4.98	27,148,512	21,202,082	78.10%	1,943,573	7.16%	4,002,857	14.74%	18208045	85.88%	21,933	1
e14_SI	e14	SI	18	18	26.04.18	do26279	1	500	6.16	17,394,443	15,118,264	86.91%	1,065,859	6.13%	1,210,320	6.96%	12954009	85.68%	21,779	1
e14_SII	e14	SII	17	18	26.04.18	do26280	1	474	5.06	19,982,005	17,456,879	87.36%	1,152,538	5.77%	1,372,588	6.87%	14631050	83.81%	22,200	1
e14_SIII	e14	SIII	16	18	26.04.18	do26281	1	467	3.98	10,890,982	9,421,084	86.50%	658,028	6.04%	811,870	7.45%	7986486	84.77%	20,732	1
e15_SI	e15	SI	18	18	26.04.18	do26282	1	474	3.18	17,078,623	13,481,094	78.94%	1,172,388	6.86%	2,425,141	14.20%	10788866	80.03%	22,038	1
e15_SII	e15	SII	17	18	26.04.18	do26283	1	497	2.9	10,364,313	8,441,580	81.45%	678,653	6.55%	1,244,080	12.00%	7027280	83.25%	20,432	1
e15_SIII	e15	SIII	16	18	26.04.18	do26284	1	449	3.8	13,957,817	10,892,022	78.04%	969,397	6.95%	2,096,398	15.02%	9125957	83.79%	21,273	1
e27_SI	e27	SI	18	18	01.06.18	do26317	1	489	13.08	14,965,632	13,169,244	88.00%	873,075	5.83%	923,313	6.17%	11188830	84.96%	22,448	1
e27_SII	e27	SII	17	18	01.06.18	do26318	1	446	6.7	18,204,147	15,468,276	84.97%	1,138,716	6.26%	1,597,155	8.77%	13239896	85.59%	20,979	1
e27_SIII	e27	SIII	16	18	01.06.18	do26319	1	378	1.91	23,044,782	15,687,604	68.07%	1,957,936	8.50%	5,399,242	23.43%	13016231	82.97%	21,107	1
e28_SI	e28	SI	18	18	01.06.18	do26320	1	424	14.2	21,814,598	18,827,750	86.31%	1,407,966	6.45%	1,578,882	7.24%	16485741	87.56%	21,396	1
e28_SII	e28	SII	17	18	01.06.18	do26321	1	435	8.02	16,757,748	14,073,742	83.98%	1,088,761	6.50%	1,595,245	9.52%	12076425	85.81%	20,790	1
e28_SIII	e28	SIII	16	18	01.06.18	do26322	1	484	2.52	15,095,217	12,315,332	81.58%	1,033,774	6.85%	1,746,111	11.57%	10363915	84.15%	20,544	1
e1_SI	e1	SI	21	21	05.04.18	do26259	1	479	5.66	19,115,686	15,918,562	83.27%	1,309,923	6.85%	1,887,201	9.87%	13636604	85.66%	21,988	1
e1_SII	e1	SII	20	21	05.04.18	do26260	1	522	4.32	16,223,856	13,632,341	84.03%	1,052,151	6.49%	1,539,364	9.49%	11380276	83.48%	22,523	1
e1_SIII	e1	SIII	19	21	05.04.18	do26261	1	477	3.76	16,078,906	13,362,203	83.10%	1,060,086	6.59%	1,656,617	10.30%	11447256	85.67%	21,115	1
e2_SI	e2	SI	21	21	05.04.18	do26262	1	469	4.46	17,745,006	14,931,863	84.15%	1,149,455	6.48%	1,663,688	9.38%	12962117	86.81%	20,462	1
e2_SIII	e2	SIII	19	21	05.04.18	do26263	1	412	3.68	26,208,112	21,044,414	80.30%	1,732,159	6.61%	3,431,539	13.09%	17537606	83.34%	22,003	1
e3_SI	e3	SI	21	21	05.04.18	do26264	1	466	8.98	12,070,510	10,319,762	85.50%	773,006	6.40%	977,742	8.10%	8651097	83.83%	21,326	1
e3_SII	e3	SII	20	21	05.04.18	do26265	1	488	7.88	8,914,760	7,662,977	85.96%	558,011	6.26%	693,772	7.78%	6375753	83.20%	20,696	1
e3_SIII	e3	SIII	19	21	05.04.18	do26266	1	514	9.92	9,473,279	8,231,138	86.89%	598,800	6.32%	643,341	6.79%	6683762	81.20%	24,827	1
e13_SI	e13	SI	21	21	26.04.18	do26276	1	469	9.62	19,031,213	16,444,192	86.41%	1,187,389	6.24%	1,399,632	7.35%	13907261	84.57%	21,801	1
e13_SII	e13	SII	20	21	26.04.18	do26277	1	500	2.4	5,274,715	4,539,096	86.05%	342,472	6.49%	393,147	7.45%	3854885	84.93%	18,565	1
e13_SIII	e13	SIII	19	21	26.04.18	do26278	1	496	3.76	12,628,888	10,178,030	80.59%	797,088	6.31%	1,653,770	13.10%	7895052	77.57%	22,795	1
e19_SI	e19	SI	25	25	20.05.18	do26294	1	504	9.12	14,886,262	12,901,995	86.67%	887,518	5.96%	1,096,749	7.37%	10948970	84.86%	21,142	1
e19_SII	e19	SII	24	25	20.05.18	do26295	1	501	6.7	16,366,161	14,062,380	85.92%	1,037,077	6.34%	1,266,704	7.74%	12199572	86.75%	21,038	1
e19_SIII	e19	SIII	23	25	20.05.18	do26296	1	518	9.94	17,630,796	15,148,231	85.92%	1,065,552	6.04%	1,417,013	8.04%	12944414	85.45%	21,879	1
e20_SI	e20	SI	25	25	20.05.18	do26297	1	446	3.9	21,319,850	17,305,153	81.17%	1,368,369	6.42%	2,646,328	12.41%	14187805	81.99%	22,506	1
e20_SII	e20	SII	24	25	20.05.18	do26298	1	415	7.82	15,418,029	12,469,799	80.88%	921,318	5.98%	2,026,912	13.15%	10109585	81.07%	22,276	1
e20_SIII	e20	SIII	23	25	20.05.18	do26299	1	422	3	16,551,117	13,177,452	79.62%	974,416	5.89%	2,399,249	14.50%	10478383	79.52%	22,499	1
e21_SI	e21	SI	25	25	24.05.18	do26300	1	465	11.9	14,032,161	12,045,302	85.84%	833,824	5.94%	1,153,035	8.22%	10029660	83.27%	21,805	1
e21_SIII	e21	SIII	23	25	24.05.18	do26301	1	586	6.98	19,797,880	17,365,881	87.72%	1,175,918	5.94%	1,256,081	6.34%	14389506	82.86%	23,832	1
e22_SI	e22	SI	25	25	24.05.18	do26302	1	508	7.8	14,736,983	12,770,927	86.66%	1,042,722	7.08%	923,334	6.27%	11009395	86.21%	21,737	1
e22_SII	e22	SII	24	25	24.05.18	do26303	1	557	8.86	16,622,239	14,928,806	89.81%	944,611	5.68%	748,822	4.50%	12848950	86.07%	21,395	1
e22_SIII	e22	SIII	23	25	24.05.18	do26304	1	520	9.14	16,896,833	15,083,645	89.27%	983,086	5.82%	830,102	4.91%	13056410	86.56%	21,854	1

sampleName	embryo	somite	somiteNumber	somiteStage	collectionDate	RNA_ID	RNA_QC	size (bp)	concentration [nM]	totalFragments	uniquelyMapped		multimapped		unmapped		uniqueInExons		numberGenes	QCpass
e23_SI	e23	SI	27	27	24.05.18	do26305	1	590	26.8	7,988,810	6,947,827	86.97%	495,655	6.20%	545,328	6.83%	5977893	86.04%	20,149	1
e23_SII	e23	SII	26	27	24.05.18	do26306	1	510	8.82	21,886,661	19,168,094	87.58%	1,327,708	6.07%	1,390,859	6.35%	16418749	85.66%	23,407	1
e23_SIII	e23	SIII	25	27	24.05.18	do26307	1	385	1.6	22,118,311	13,060,634	59.05%	1,803,166	8.15%	7,254,511	32.80%	9098507	69.66%	23,499	1
e24_SI	e24	SI	27	27	24.05.18	do26308	1	473	12.98	47,578,277	41,692,027	87.63%	3,002,440	6.31%	2,883,810	6.06%	35909124	86.13%	25,226	1
e24_SII	e24	SII	26	27	24.05.18	do26309	1	437	8.88	38,420,055	31,791,135	82.75%	3,309,846	8.61%	3,319,074	8.64%	27773459	87.36%	24,161	1
e24_SIII	e24	SIII	25	27	24.05.18	do26310	1	391	13.3	31,117,702	26,144,820	84.02%	2,177,639	7.00%	2,795,243	8.98%	22629196	86.55%	23,983	1
e25_SI	e25	SI	27	27	24.05.18	do26311	1	486	14.8	24,509,980	20,539,533	83.80%	2,208,717	9.01%	1,761,730	7.19%	18139676	88.32%	23,452	1
e25_SII	e25	SII	26	27	24.05.18	do26312	1	546	14.12	21,765,155	19,324,858	88.79%	1,377,513	6.33%	1,062,784	4.88%	16730504	86.58%	23,325	1
e25_SIII	e25	SIII	25	27	24.05.18	do26313	1	576	16	21,884,352	19,485,576	89.04%	1,364,596	6.24%	1,034,180	4.73%	17027011	87.38%	23,057	1
e34_SI	e34	SI	27	27	14.06.18	do26337	1	450	16.66	33,546,238	29,193,121	87.02%	2,262,418	6.74%	2,090,699	6.23%	24729002	84.71%	25,963	1
e34_SII	e34	SII	26	27	14.06.18	do26338	1	476	17.52	24,831,126	22,068,851	88.88%	1,520,308	6.12%	1,241,967	5.00%	18430704	83.51%	25,214	1
e17_SI	e17	SI	35	35	27.04.18	do26288	1	507	15.8	15,111,788	13,227,335	87.53%	923,753	6.11%	960,700	6.36%	11068316	83.68%	22,645	1
e17_SII	e17	SII	34	35	27.04.18	do26289	1	528	12.2	88,342	71,120	80.51%	6,268	7.10%	10,954	12.40%	59881	84.20%	8,159	0
e17_SIII	e17	SIII	33	35	27.04.18	do26290	1	543	7.82	24,112,273	20,926,547	86.79%	1,474,496	6.12%	1,711,230	7.10%	17689437	84.53%	23,960	1
e29_SI	e29	SI	35	35	11.06.18	do26323	1	468	13.76	27,241,840	23,423,979	85.99%	2,066,584	7.59%	1,751,277	6.43%	20182512	86.16%	23,686	1
e29_SII	e29	SII	34	35	11.06.18	do26324	1	470	12.02	19,935,563	17,051,356	85.53%	1,453,390	7.29%	1,430,817	7.18%	14463539	84.82%	24,695	1
e29_SIII	e29	SIII	33	35	11.06.18	do26325	1	528	9.04	12,410,163	10,739,506	86.54%	786,704	6.34%	883,953	7.12%	8895134	82.83%	22,049	1
e30_SI	e30	SI	35	35	11.06.18	do26326	1	489	14.42	15,437,320	13,568,906	87.90%	928,398	6.01%	940,016	6.09%	11259137	82.98%	23,051	1
e30_SII	e30	SII	34	35	11.06.18	do26327	1	497	12.5	14,387,094	12,502,964	86.90%	923,627	6.42%	960,503	6.68%	10795415	86.34%	21,445	1
e30_SIII	e30	SIII	33	35	11.06.18	do26328	1	492	4.94	11,655,620	9,978,735	85.61%	782,173	6.71%	894,712	7.68%	8253505	82.71%	21,641	1
e31_SI	e31	SI	35	35	11.06.18	do26329	1	506	7.46	23,218,716	19,641,242	84.59%	1,452,083	6.25%	2,125,391	9.15%	16435852	83.68%	23,301	1
e31_SII	e31	SII	34	35	11.06.18	do26330	1	469	10.14	15,194,366	13,221,314	87.01%	960,989	6.32%	1,012,063	6.66%	11083470	83.83%	22,647	1
e31_SIII	e31	SIII	33	35	11.06.18	do26331	1	496	7.1	22,757,132	19,427,603	85.37%	1,415,503	6.22%	1,914,026	8.41%	16270683	83.75%	24,511	1
e32_SI	e32	SI	35	35	14.06.18	do26332	1	468	12.3	25,719,125	22,599,520	87.87%	1,595,615	6.20%	1,523,990	5.93%	18921965	83.73%	24,665	1
e32_SIII	e32	SIII	33	35	14.06.18	do26333	1	428	2.52	17,945,810	13,162,156	73.34%	1,649,726	9.19%	3,133,928	17.46%	10670675	81.07%	20,929	1
e33_SI	e33	SI	35	35	14.06.18	do26334	1	453	21.6	23,850,064	20,744,926	86.98%	1,591,931	6.67%	1,513,207	6.34%	17885958	86.22%	22,422	1
e33_SII	e33	SII	34	35	14.06.18	do26335	1	448	7.42	23,934,429	20,276,034	84.71%	1,833,093	7.66%	1,825,302	7.63%	17363611	85.64%	25,072	1
e33_SIII	e33	SIII	33	35	14.06.18	do26336	1	476	11.84	39,323,747	33,632,900	85.53%	3,004,980	7.64%	2,685,867	6.83%	29112812	86.56%	25,033	1

Table S2 | Quality control statistics from the RNA-seq libraries, related to STAR Methods.

Only one sample failed QC (in red) due to insufficient sequencing depth (totalFragments). The 'uniqueInExons' column indicates the number of fragments uniquely mapped to the genome that also can be assigned unambiguously to annotated exons.

ATAC-seq																					
sampleName	embryo	somite	somiteNumber	somiteStage	collectionDate	ATAC_ID	ATAC_QC	size (bp)	concentration [nM]	totalFragments	mapped		unique		goodQuality		insertSizeDist	numberPeaks	readsInPeaks	TSSenrichment	QCpass
e5_SI	e5	SI	8	8	06.04.18	do26190	1	486	5.78	41,520,261	37,764,252	90.95%	21,474,949	51.72%	20,125,635	93.72%	4	53,210	3,382,072	6.31	1
e5_SII	e5	SII	7	8	06.04.18	do26191	0	425	9.5	30,371,932	27,596,028	90.86%	16,843,741	55.46%	15,485,088	91.93%	3	7	481	2.30	0
e5_SIII	e5	SIII	6	8	06.04.18	do26192	0	469	10.76	32,466,242	29,472,826	90.78%	19,147,566	58.98%	18,121,596	94.64%	4	2,811	80,214	2.96	0
e6_SI	e6	SI	8	8	06.04.18	do26193	0	425	5.86	49,200,969	44,628,904	90.71%	26,846,785	54.57%	24,700,063	92.00%	3	14,058	554,250	3.75	0
e6_SII	e6	SII	7	8	06.04.18	do26194	0	439	7.1	43,376,906	39,417,510	90.87%	23,855,029	54.99%	21,925,536	91.91%	3	683	18,535	2.56	0
e16_SI	e16	SI	8	8	26.04.18	do26205	0	341	6.26	19,523,615	17,703,369	90.68%	10,864,615	55.65%	9,290,851	85.51%	1	5,273	86,320	4.50	0
e16_SII	e16	SII	7	8	26.04.18	do26206	1	342	7.88	20,962,982	19,057,936	90.91%	12,212,591	58.26%	10,659,160	87.28%	1	20,477	488,795	5.54	1
e16_SIII	e16	SIII	6	8	26.04.18	do26207	1	420	6.08	25,555,851	23,211,869	90.83%	13,987,736	54.73%	12,357,920	88.35%	2	39,098	1,036,066	5.28	1
e26_SI	e26	SI	8	8	01.06.18	do26233	1	437	7.74	69,169,028	62,813,968	90.81%	38,236,045	55.28%	35,810,413	93.66%	3	35,081	2,579,196	4.46	1
e26_SII	e26	SII	7	8	01.06.18	do26234	1	372	5.04	43,572,763	39,586,640	90.85%	25,586,947	58.72%	23,131,659	90.40%	2	23,985	1,054,320	4.31	1
e26_SIII	e26	SIII	6	8	01.06.18	do26235	1	444	8.12	67,280,508	61,033,758	90.72%	36,787,364	54.68%	34,969,382	95.06%	4	28,442	1,908,884	4.01	1
e9_SI	e9	SI	18	18	12.04.18	do26195	1	423	6.84	32,624,338	29,564,356	90.62%	16,503,078	50.59%	14,656,807	88.81%	3	51,754	2,539,535	6.73	1
e9_SII	e9	SII	17	18	12.04.18	do26196	0	414	9.7	44,416,466	40,355,298	90.86%	23,754,141	53.48%	21,837,354	91.93%	2	13,212	511,689	3.75	0
e9_SIII	e9	SIII	16	18	12.04.18	do26197	1	437	7.46	48,591,338	44,136,194	90.83%	26,668,661	54.88%	24,342,954	91.28%	3	33,463	1,727,050	4.67	1
e14_SII	e14	SII	17	18	26.04.18	do26201	1	473	7.72	57,427,746	52,127,849	90.77%	26,828,129	46.72%	25,503,529	95.06%	4	57,994	5,065,873	6.82	1
e14_SIII	e14	SIII	16	18	26.04.18	do26202	0	425	7.56	43,138,589	39,100,379	90.64%	23,329,698	54.08%	21,628,928	92.71%	0	4,411	129,143	2.83	0
e15_SI	e15	SI	18	18	26.04.18	do26203	1	393	6.8	21,142,333	19,168,513	90.66%	11,326,869	53.57%	9,826,753	86.76%	2	32,405	866,781	6.03	1
e15_SII	e15	SII	17	18	26.04.18	do26204	1	447	6.64	48,507,019	44,129,083	90.97%	25,268,838	52.09%	23,615,417	93.46%	3	49,130	3,306,248	6.09	1
e27_SI	e27	SI	18	18	01.06.18	do26236	1	380	7.66	61,879,228	56,150,469	90.74%	31,437,853	50.81%	28,226,636	89.79%	2	67,413	5,903,924	7.73	1
e27_SII	e27	SII	17	18	01.06.18	do26237	1	364	9.04	50,280,194	45,713,793	90.92%	28,119,885	55.93%	24,868,793	88.44%	2	48,291	2,717,378	6.50	1
e27_SIII	e27	SIII	16	18	01.06.18	do26238	1	348	14.8	43,095,342	39,106,388	90.74%	26,296,450	61.02%	23,083,729	87.78%	0	21,220	804,344	4.16	1
e28_SI	e28	SI	18	18	01.06.18	do26239	0	303	17.8	28,015,581	25,479,427	90.95%	18,180,641	64.89%	15,657,252	86.12%	0	1,152	19,708	2.66	0
e28_SII	e28	SII	17	18	01.06.18	do26240	1	334	10.1	35,113,967	31,943,185	90.97%	20,978,024	59.74%	18,137,094	86.46%	0	22,931	683,499	4.74	1
e28_SIII	e28	SIII	16	18	01.06.18	do26241	1	390	5.97	39,153,744	35,575,751	90.86%	21,865,433	55.85%	19,216,659	87.89%	1	49,674	2,175,964	6.15	1
e1_SI	e1	SI	21	21	05.04.18	do26181	1	328	18	26,285,398	23,805,193	90.56%	15,699,543	59.73%	13,500,945	86.00%	1	35,243	1,267,894	7.18	1
e1_SII	e1	SII	20	21	05.04.18	do26182	1	368	18.6	27,923,115	25,363,674	90.83%	16,868,650	60.41%	14,956,907	88.67%	2	31,206	1,206,547	6.58	1
e1_SIII	e1	SIII	19	21	05.04.18	do26183	0	344	18.1	22,545,472	20,472,674	90.81%	14,252,736	63.22%	12,675,623	88.93%	0	6	278	2.14	0
e2_SI	e2	SI	21	21	05.04.18	do26184	0	396	8.87	30,698,288	27,882,112	90.83%	18,694,786	60.90%	17,124,982	91.60%	2	150	3,419	2.70	0
e2_SII	e2	SII	20	21	05.04.18	do26185	1	421	6.42	42,503,669	38,660,336	90.96%	23,286,344	54.79%	21,466,478	92.18%	3	33,724	1,934,851	5.67	1
e2_SIII	e2	SIII	19	21	05.04.18	do26186	0	337	12.1	21,572,118	19,607,938	90.89%	14,192,023	65.79%	12,516,018	88.19%	1	81	1,816	2.87	0
e3_SI	e3	SI	21	21	05.04.18	do26187	0	376	14.9	27,883,412	25,342,010	90.89%	16,961,929	60.83%	15,491,632	91.33%	2	83	2,439	3.19	0
e3_SII	e3	SII	20	21	05.04.18	do26188	1	409	8.44	34,035,542	30,921,806	90.85%	20,205,853	59.37%	18,337,752	90.75%	2	21,201	834,258	4.99	1
e3_SIII	e3	SIII	19	21	05.04.18	do26189	0	414	11.2	33,237,354	30,189,528	90.83%	19,287,813	58.03%	17,771,925	92.14%	3	12,144	428,856	4.02	0
e13_SI	e13	SI	21	21	26.04.18	do26198	1	441	7.38	36,859,933	33,359,197	90.50%	16,685,449	45.27%	15,323,118	91.84%	3	52,140	2,841,072	7.44	1
e13_SII	e13	SII	20	21	26.04.18	do26199	1	489	6.22	46,126,231	41,858,744	90.75%	21,185,446	45.93%	20,090,201	94.83%	4	51,387	4,251,284	7.61	1
e13_SIII	e13	SIII	19	21	26.04.18	do26200	1	447	6.76	31,970,370	28,986,376	90.67%	15,388,171	48.13%	14,096,109	91.60%	3	46,888	2,137,431	6.38	1
e19_SI	e19	SI	25	25	20.05.18	do26214	1	421	11.4	31,525,091	28,547,683	90.56%	16,796,287	53.28%	15,641,546	93.13%	4	50,824	3,834,123	8.56	1
e19_SII	e19	SII	24	25	20.05.18	do26215	1	408	6.19	33,277,602	30,182,182	90.70%	17,514,786	52.63%	15,606,419	89.10%	2	49,316	2,644,494	7.47	1
e19_SIII	e19	SIII	23	25	20.05.18	do26216	0	415	8.24	46,082,073	41,704,050	90.50%	25,551,639	55.45%	23,172,217	90.69%	2	14,305	614,083	4.16	0
e20_SI	e20	SI	25	25	20.05.18	do26217	1	468	5.3	38,450,312	34,902,562	90.77%	18,187,382	47.30%	16,856,794	92.68%	3	47,689	2,977,092	7.50	1
e20_SII	e20	SII	24	25	20.05.18	do26218	1	419	9.44	36,502,685	33,219,443	91.01%	20,946,689	57.38%	19,397,511	92.60%	2	30,605	1,305,794	5.39	1
e20_SIII	e20	SIII	23	25	20.05.18	do26219	1	441	6.75	45,028,161	40,914,608	90.86%	26,120,484	58.01%	24,141,739	92.42%	3	39,115	2,386,067	6.08	1
e21_SI	e21	SI	25	25	24.05.18	do26220	1	404	8.26	18,193,619	16,499,754	90.69%	8,569,971	47.10%	7,956,487	92.84%	2	48,557	2,683,644	9.77	1
e21_SII	e21	SII	24	25	24.05.18	do26221	1	461	7.58	40,543,973	36,810,397	90.79%	20,627,302	50.88%	18,895,591	91.60%	3	51,010	3,118,988	7.06	1
e21_SIII	e21	SIII	23	25	24.05.18	do26222	1	438	7.32	35,421,485	32,182,060	90.85%	16,672,562	47.07%	15,308,685	91.82%	3	55,913	3,325,865	7.46	1
e22_SI	e22	SI	25	25	24.05.18	do26223	1	433	5.7	61,751,926	56,157,557	90.94%	30,769,038	49.83%	28,310,041	92.01%	3	59,371	5,535,868	7.42	1
e22_SII	e22	SII	24	25	24.05.18	do26224	1	421	7.34	41,092,266	37,305,894	90.79%	21,255,700	51.73%	19,013,735	89.45%	2	44,161	2,427,196	6.47	1
e22_SIII	e22	SIII	23	25	24.05.18	do26225	1	407	8.58	61,349,519	55,715,809	90.82%	31,691,112	51.66%	28,654,120	90.42%	2	45,151	3,003,688	6.20	1
e23_SI	e23	SI	27	27	24.05.18	do26226	1	237	5.88	16,621,773	15,083,965	90.75%	11,271,492	67.81%	9,699,800	86.06%	0	26,033	494,922	6.38	1
e23_SII	e23	SII	26	27	24.05.18	do26227	0	280	32.2	33,069,762	30,028,502	90.80%	21,955,382	66.39%	19,092,921	86.96%	0	2,652	58,996	2.79	0
e23_SIII	e23	SIII	25	27	24.05.18	do26228	0	372	8.05	32,049,089	29,116,276	90.85%	19,407,711	60.56%	17,276,310	89.02%	2	2,811	70,947	3.57	0
e24_SI	e24	SI	27	27	24.05.18	do26229	1	343	14.8	34,700,675	31,517,200	90.83%	21,766,776	62.73%	19,015,349	87.36%	1	28,575	1,122,828	5.36	1
e24_SII	e24	SII	26	27	24.05.18	do26230	1	405	6.22	36,776,104	33,402,398	90.83%	20,508,945	55.77%	18,505,572	90.23%	2	54,228	3,130,807	7.42	1
e24_SIII	e24	SIII	25	27	24.05.18	do26231	1	361	8.52	46,008,731	41,773,412	90.79%	28,414,076	61.76%	24,820,262	87.35%	1	27,050	1,227,985	4.74	1
e25_SI	e25	SI	27	27	24.05.18	do26232	1	380	9.48	51,623,765	46,831,482	90.72%	28,176,462	54.58%	25,520,753	90.57%	2	42,475	3,296,630	7.68	1
e34_SI	e34	SI	27	27	14.06.18	do26256	0	356	15.7	27,614,285	25,097,089	90.88%	18,076,382	65.46%	16,779,282	92.82%	0	1,239	25,151	2.23	0
e34_SII	e34	SII	26	27	14.06.18	do26257	0	274	14.3	58,378,625	52,989,711	90.77%	38,558,648	66.05%	35,014,726	90.81%	0	813	23,158	1.97	0
e34_SIII	e34	SIII	25	27	14.06.18	do26258	0	324	30	28,666,363	26,027,868	90.80%	18,397,554	64.18%	17,063,699	92.75%	0	1,653	35,847	2.64	0

sampleName	embryo	somite	somiteNumber	somiteStage	collectionDate	ATAC_ID	ATAC_QC	size (bp)	concentration [nM]	totalFragments	mapped		unique		goodQuality		insertSizeDist	numberPeaks	readsInPeaks	TSSenrichment	QCpass
e17_SI	e17	SI	35	35	27.04.18	do26208	1	466	7.94	45,492,062	41,369,622	90.94%	22,245,164	48.90%	21,162,155	95.13%	4	64,529	5,970,483	7.95	1
e17_SII	e17	SII	34	35	27.04.18	do26209	1	449	7.52	39,016,972	35,453,023	90.87%	19,977,394	51.20%	18,309,152	91.65%	3	33,926	1,614,976	5.28	1
e17_SIII	e17	SIII	33	35	27.04.18	do26210	1	382	9.8	32,048,955	29,156,005	90.97%	18,614,091	58.08%	16,632,055	89.35%	1	19,899	625,586	4.76	1
e29_SI	e29	SI	35	35	11.06.18	do26242	0	535	10.86	86,308,470	78,688,161	91.17%	42,112,412	48.79%	41,825,316	99.32%	0	9,935	458,883	1.72	0
e29_SII	e29	SII	34	35	11.06.18	do26243	1	372	15	37,337,982	33,945,412	90.91%	23,211,427	62.17%	20,946,981	90.24%	2	30,715	1,564,078	6.07	1
e29_SIII	e29	SIII	33	35	11.06.18	do26244	0	360	19.2	28,491,304	25,885,991	90.86%	17,628,708	61.87%	16,233,753	92.09%	0	7,598	198,393	3.78	0
e30_SI	e30	SI	35	35	11.06.18	do26245	1	386	10.3	35,506,847	32,308,450	90.99%	22,573,564	63.58%	20,108,032	89.08%	2	18,073	641,229	4.15	1
e30_SII	e30	SII	34	35	11.06.18	do26246	1	393	12.5	45,918,477	41,699,097	90.81%	27,273,171	59.39%	24,676,203	90.48%	2	21,036	926,212	4.45	1
e31_SI	e31	SI	35	35	11.06.18	do26247	1	262	39	40,486,472	36,709,938	90.67%	26,127,958	64.54%	23,096,416	88.40%	0	30,594	1,539,181	6.07	1
e31_SII	e31	SII	34	35	11.06.18	do26248	0	269	20.2	59,363,143	53,934,215	90.85%	39,535,843	66.60%	35,830,900	90.63%	0	10,671	496,829	3.11	0
e31_SIII	e31	SIII	33	35	11.06.18	do26249	0	334	37.1	33,880,646	30,756,782	90.78%	21,573,355	63.67%	19,623,065	90.96%	0	13,553	516,202	3.87	0
e32_SI	e32	SI	35	35	14.06.18	do26250	1	419	6.73	36,641,370	33,278,083	90.82%	22,217,580	60.64%	21,023,453	94.63%	3	23,125	1,178,262	4.81	1
e32_SII	e32	SII	34	35	14.06.18	do26251	1	436	6.36	66,155,891	60,239,521	91.06%	36,863,084	55.72%	35,286,664	95.72%	3	35,831	2,809,175	5.23	1
e32_SIII	e32	SIII	33	35	14.06.18	do26252	1	500	6.92	107,251,325	97,546,856	90.95%	52,103,795	48.58%	51,368,604	98.59%	4	25,444	2,494,536	3.80	1
e33_SI	e33	SI	35	35	14.06.18	do26253	1	424	10.76	33,566,554	30,537,273	90.98%	19,300,878	57.50%	17,850,388	92.48%	3	17,310	634,722	4.65	1
e33_SII	e33	SII	34	35	14.06.18	do26254	0	355	5.82	123,644,401	112,271,797	90.80%	98,363,427	79.55%	90,080,165	91.58%	0	6,807	449,023	1.39	0
e33_SIII	e33	SIII	33	35	14.06.18	do26255	0	519	7.78	37,302,329	34,000,527	91.15%	18,250,353	48.93%	17,779,911	97.42%	0	2,439	71,493	2.56	0

Table S3 | Quality control statistics from the ATAC-seq libraries, related to STAR Methods.

A third of the samples failed QC (in red). The 'unique' column corresponds to the number of fragments retained after removing PCR duplicates. The 'insertSizeDist', 'numberPeaks', 'readsInPeaks' and 'TSSenrichment' columns correspond to the metrics used to determine if

Data S1: microscopy images of all embryos used in this study, related to STAR methods. Embryo number corresponds to the annotation in Tables S1-3.

Stage: 8 somite pairs

e5	e6
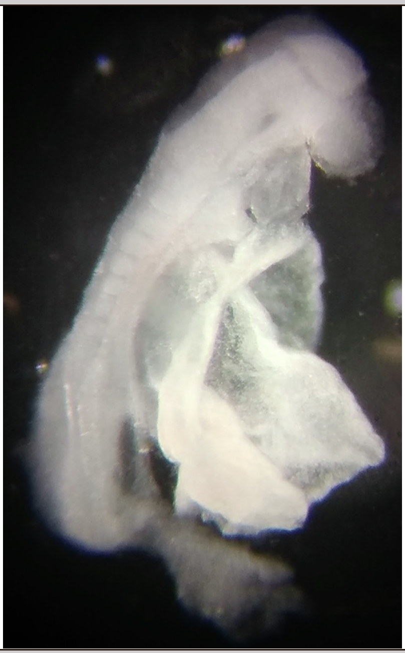	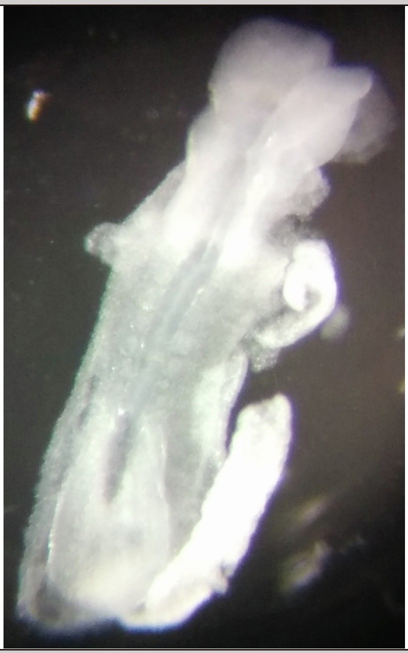
**e16**	**e26**
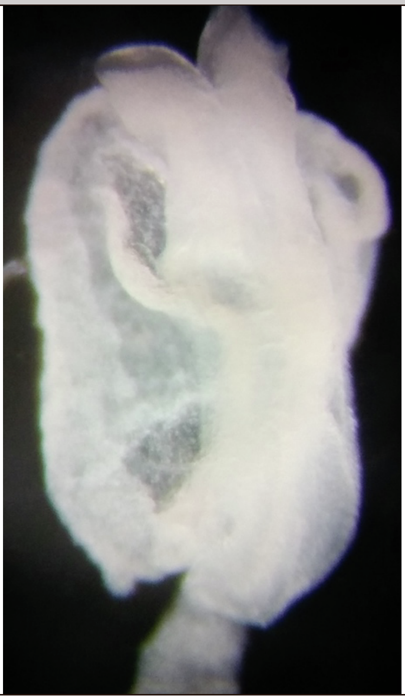	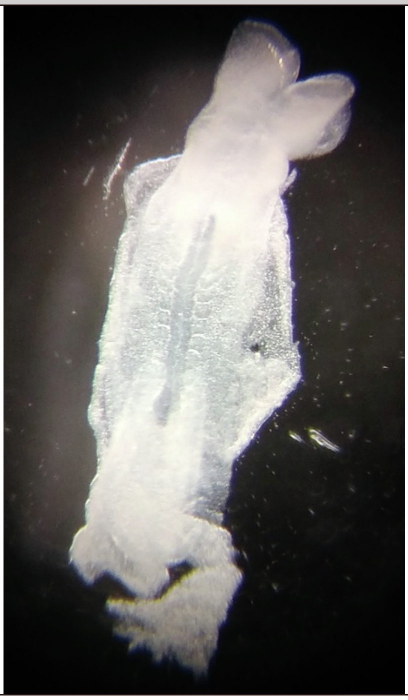

Stage: 18 somite pairs

e9	e14
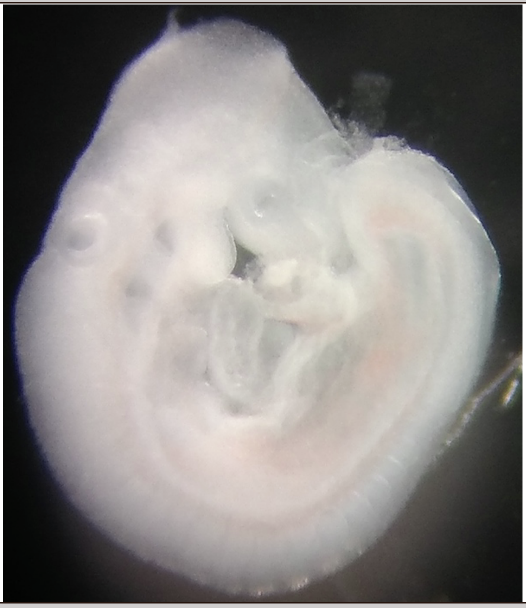	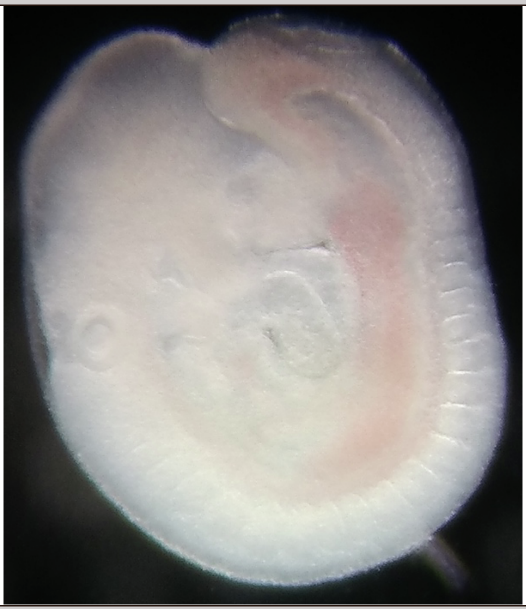
**e15**	**e27**
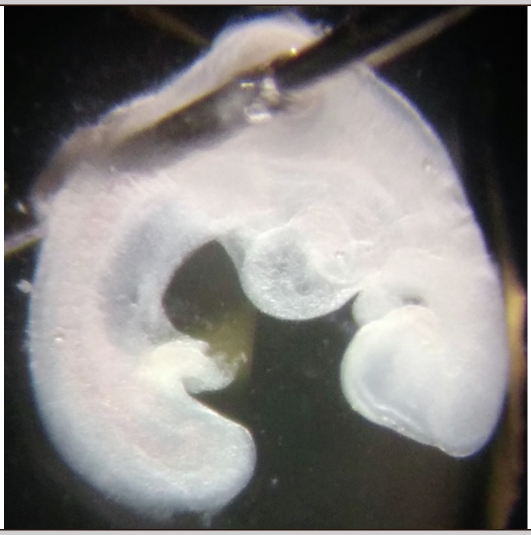	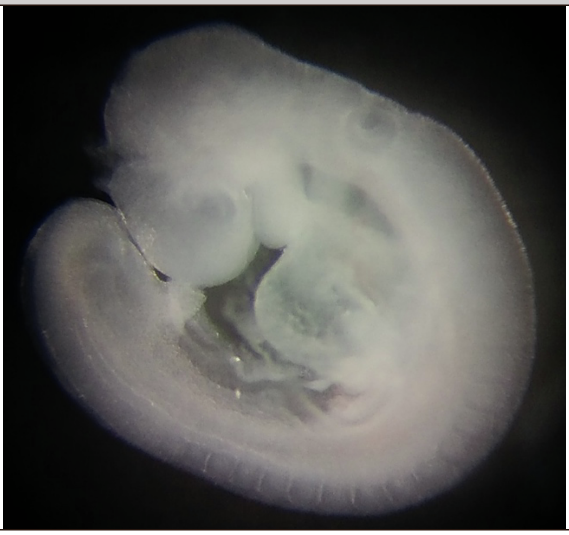
**e28**	
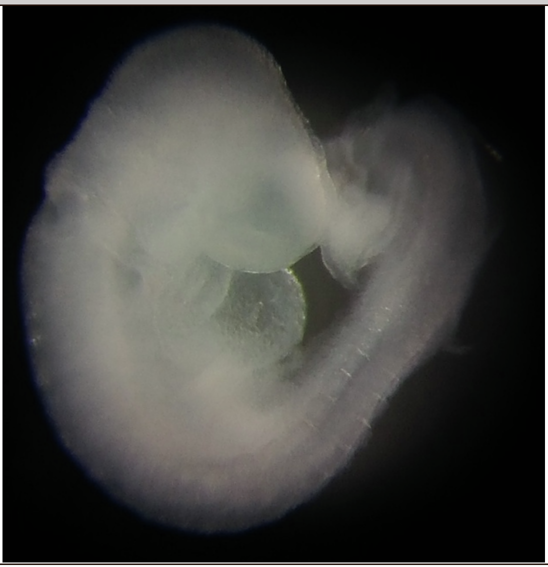	

Stage: 21 somite pairs

e1	e2
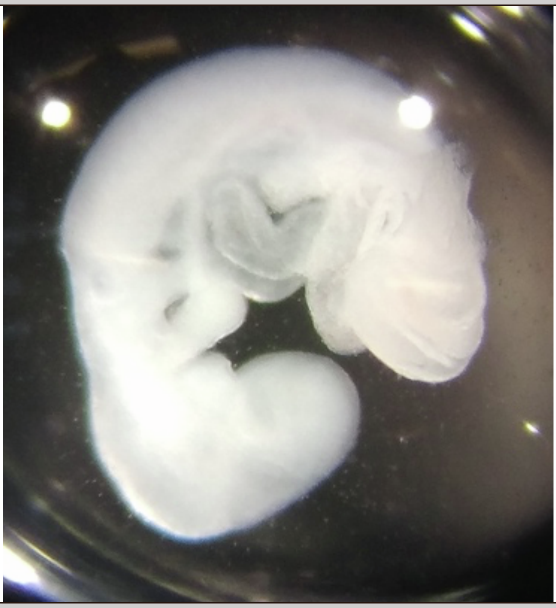	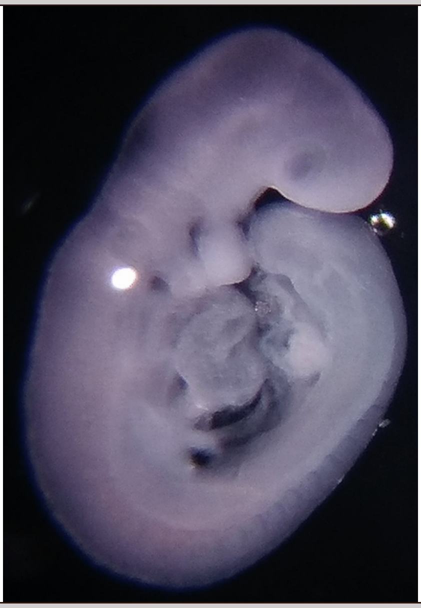
**e3**	**e13**
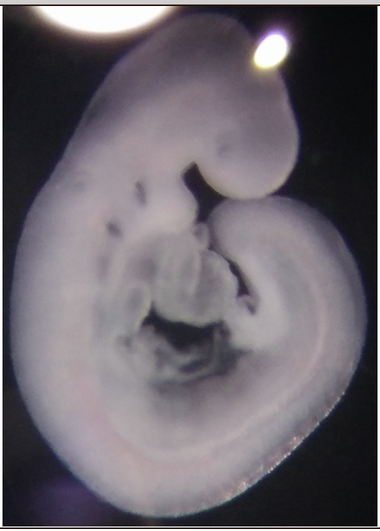	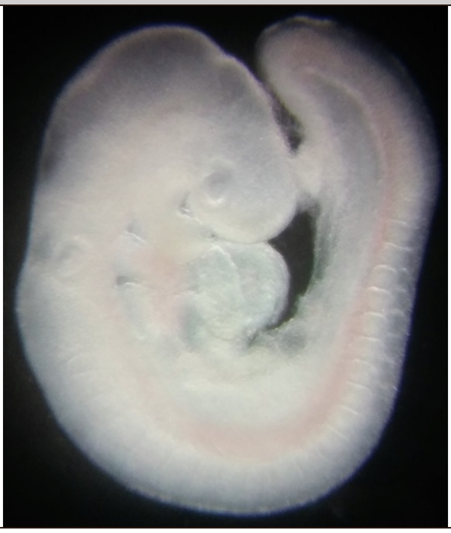

Stage: 25 somite pairs

e19	e20
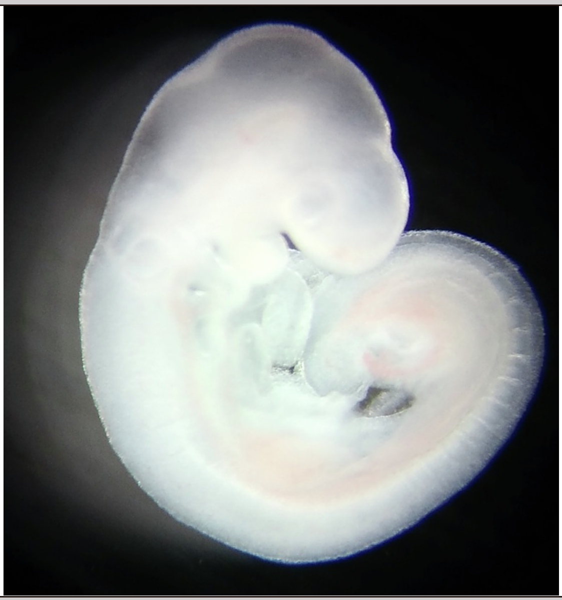	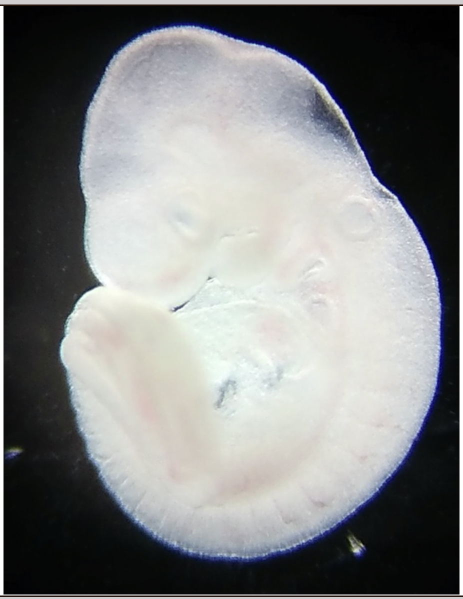
**e21**	**e22**
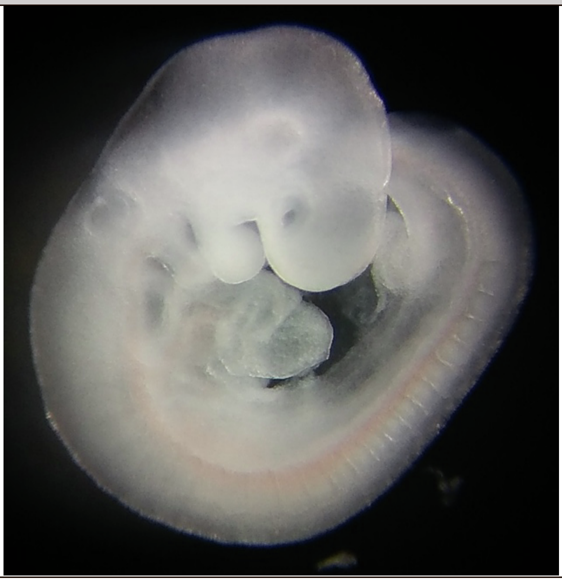	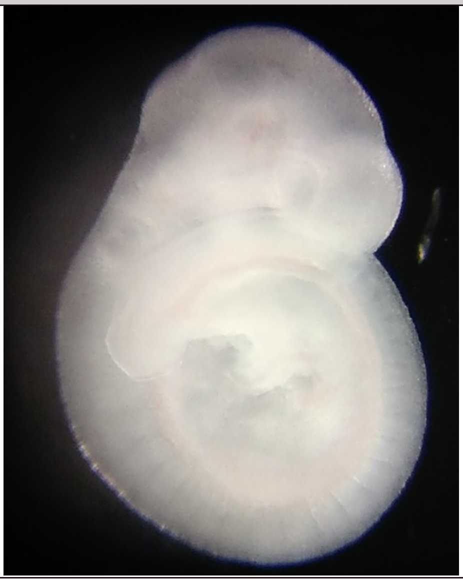

Stage: 27 somite pairs

e23	e24
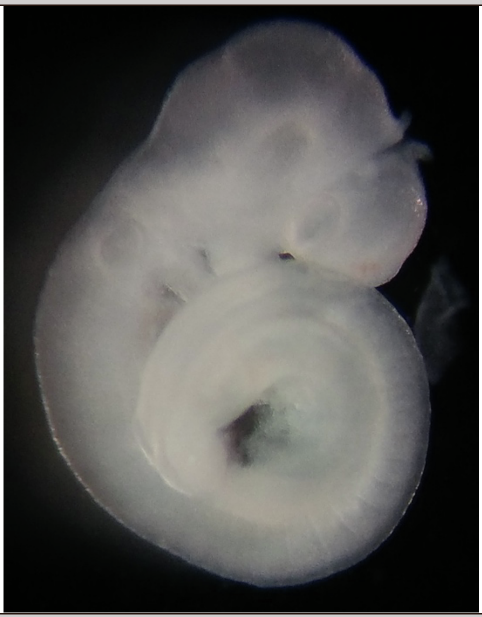	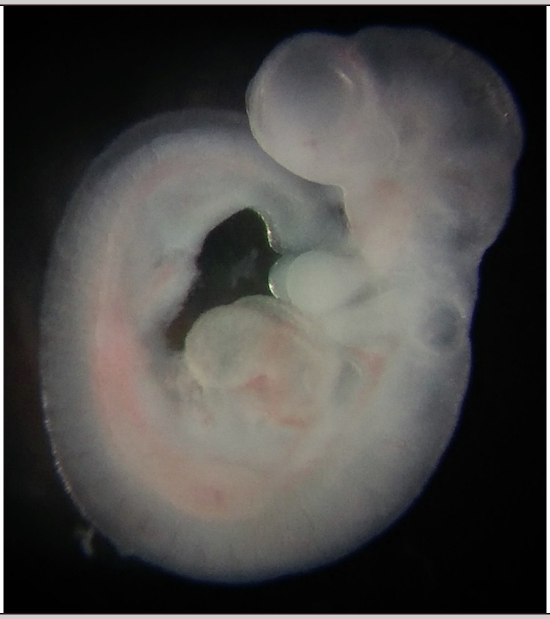
**e25**	**e34**
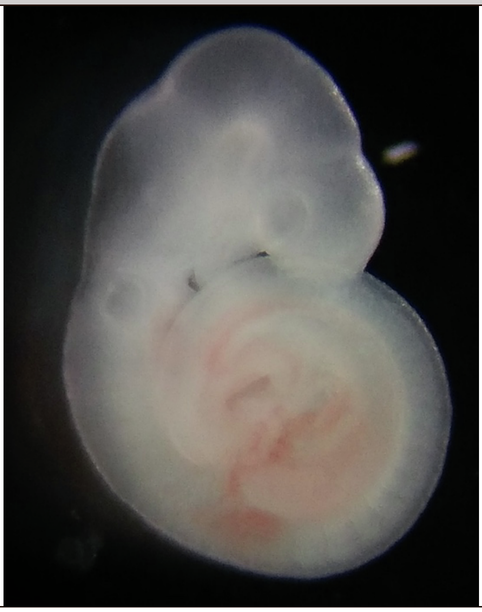	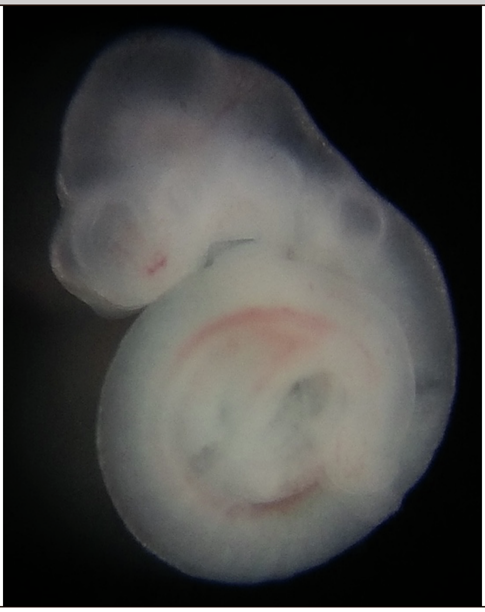

Stage: 35 somite pairs

e17	e29
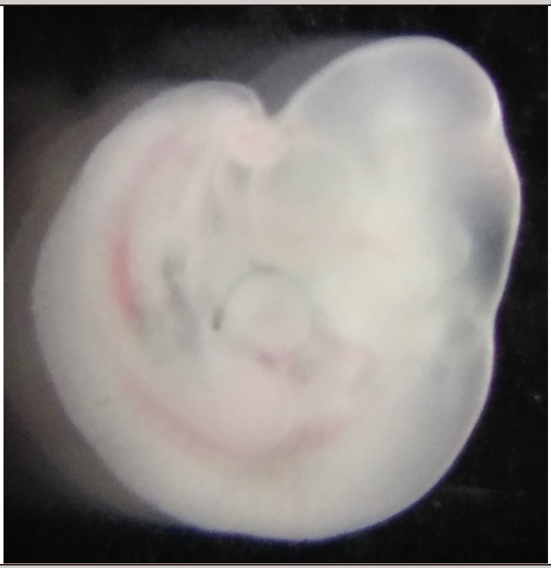	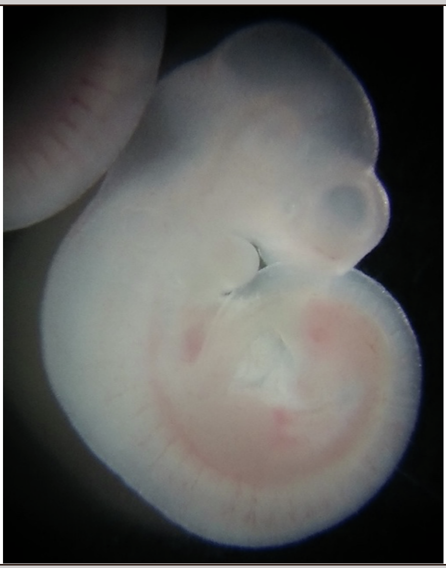
**e30**	**e31**
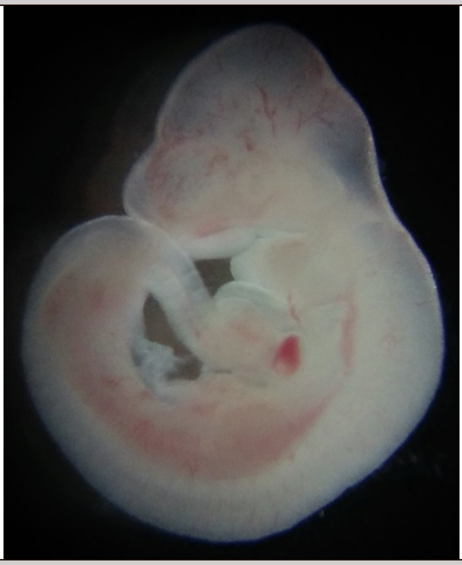	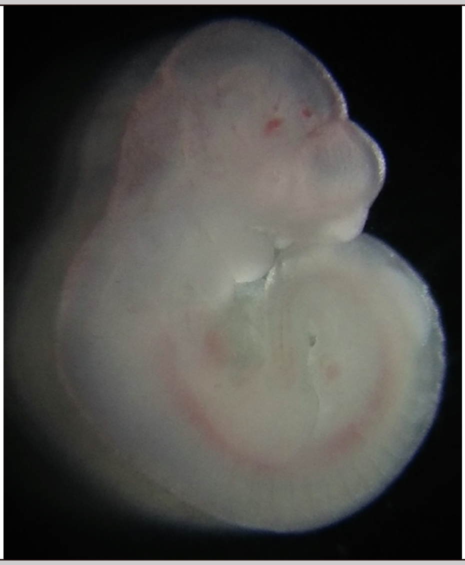
**e32**	**e33**
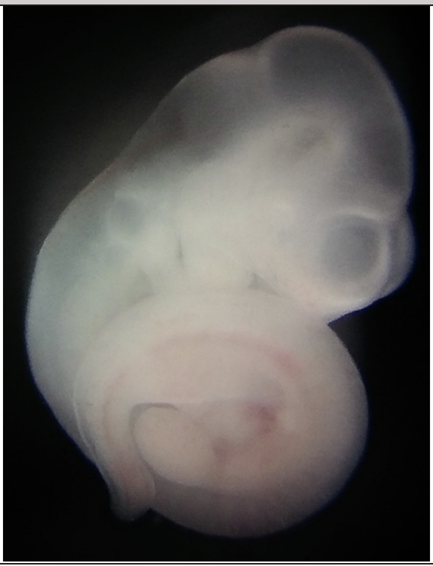	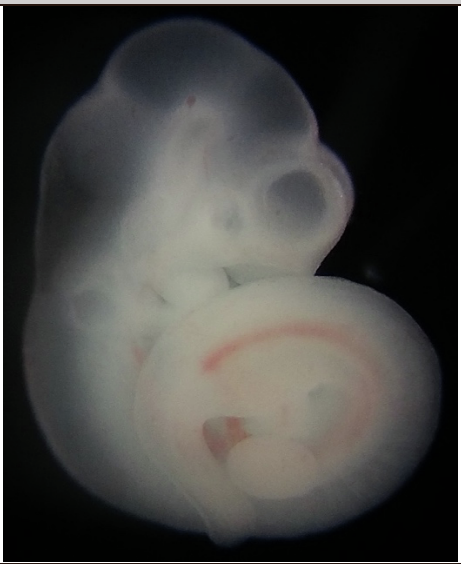

Example of dissected somites

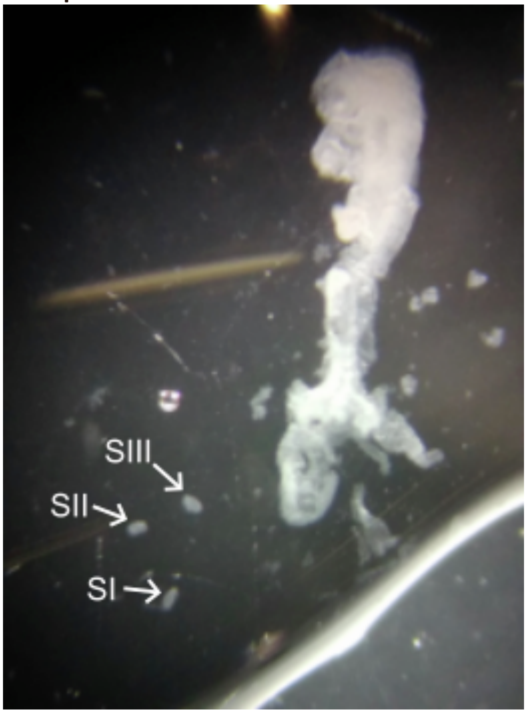

Supplement: Document S1. Figures S1–S4, Tables S1–S3, and Data S1 [file mmc1.pdf]
