# Supplementary material for: A transcriptional and regulatory map of mouse somite maturation
Source: Dev Cell. 2023 Oct 9;58(19):1983–1995.e7. doi: 10.1016/j.devcel.2023.07.003 (PMC10563765; doi:10.1016/j.devcel.2023.07.003)

Resource

Developmental Cell

A transcriptional and regulatory map of mouse somite maturation

Graphical abstract

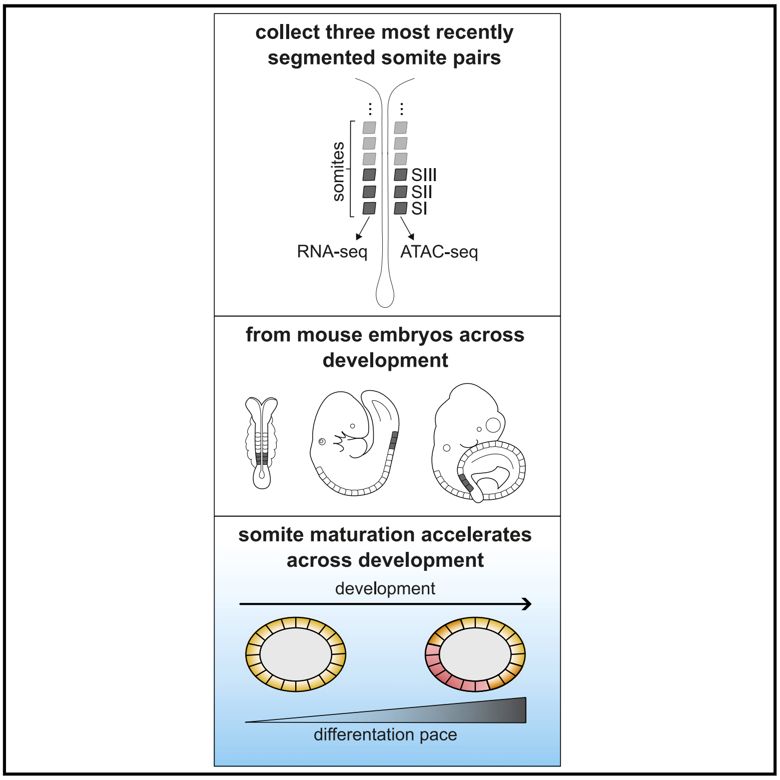

Authors

Ximena Ibarra-Soria, Elodie Thierion, Gi Fay Mok, Andrea E. Münsterberg, Duncan T. Odom, John C. Marioni

Correspondence

ximena.x.ibarra-soria@gsk.com (X.I.-S.), d.odom@dkfz-heidelberg.de (D.T.O.), marioni@ebi.ac.uk (J.C.M.)

In brief

Ibarra-Soria and Thierion et al. characterize the molecular landscape of the three most recently segmented somites across six stages of development in mouse embryos. They find that while all somites follow a common maturation program, diversification into derivative lineages accelerates across development, occurring soon after segmentation in mature embryos.

Highlights

- Somite maturation follows a conserved molecular program across development
- Somite differentiation accelerates with development
- Somites at later development commit to derivative lineages soon after segmentation

Ibarra-Soria et al., 2023, Developmental Cell *58*, 1983–1995
October 9, 2023 © 2023 The Authors. Published by Elsevier Inc.
https://doi.org/10.1016/j.devcel.2023.07.003

Developmental Cell

CellPress
OPEN ACCESS

Resource

A transcriptional and regulatory map of mouse somite maturation

Ximena Ibarra-Soria,[1,6,7,9,*] Elodie Thierion,[1,7] Gi Fay Mok,[2] Andrea E. Münsterberg,[2] Duncan T. Odom,[1,3,8,*] and John C. Marioni[1,4,5,8,*]

[1]Cancer Research UK Cambridge Institute, University of Cambridge, Li Ka Shing Centre, Robinson Way, Cambridge CB2 0RE, UK
[2]School of Biological Sciences, University of East Anglia, Norwich Research Park, Norwich NR4 7TJ, UK
[3]DKFZ, Division of Regulatory Genomics and Cancer Evolution B270, Im Neunheimer Feld 280, Heidelberg, 69120, Germany
[4]European Molecular Biology Laboratory, European Bioinformatics Institute, Wellcome Genome Campus, Cambridge CB10 1SD, UK
[5]Wellcome Sanger Institute, Wellcome Genome Campus, Cambridge CB10 1SA, UK
[6]Present address: Genomic Sciences, GSK R&D, Stevenage SG1 2NY, UK
[7]These authors contributed equally
[8]Senior author
[9]Lead contact
*Correspondence: ximena.x.ibarra-soria@gsk.com (X.I.-S.), d.odom@dkfz-heidelberg.de (D.T.O.), marioni@ebi.ac.uk (J.C.M.)
https://doi.org/10.1016/j.devcel.2023.07.003

SUMMARY

The mammalian body plan is shaped by rhythmic segmentation of mesoderm into somites, which are transient embryonic structures that form down each side of the neural tube. We have analyzed the genome-wide transcriptional and chromatin dynamics occurring within nascent somites, from early inception of somitogenesis to the latest stages of body plan establishment. We created matched gene expression and open chromatin maps for the three leading pairs of somites at six time points during mouse embryonic development. We show that the rate of somite differentiation accelerates as development progresses. We identified a conserved maturation program followed by all somites, but somites from more developed embryos concomitantly switch on differentiation programs from derivative cell lineages soon after segmentation. Integrated analysis of the somitic transcriptional and chromatin activities identified opposing regulatory modules controlling the onset of differentiation. Our results provide a powerful, high-resolution view of the molecular genetics underlying somitic development in mammals.

INTRODUCTION

The segmentation of the body plan during early embryogenesis is a fundamental and conserved feature of all vertebrate species. It results in the metameric organization of the vertebrae and the associated skeletal muscles, nerves, and blood vessels. This segmentation is established via formation of somites, which are transient embryonic structures consisting of hundreds of cells that bud off from the anterior tip of the presomitic mesoderm (PSM) on each side of the neural tube. Each pair of somites is symmetrically and rhythmically formed along the anterior-posterior axis.

The *clock and wavefront model*[1] provides a working hypothesis of how the segmentation process is controlled, by integrating spatiotemporal information from waves of transcriptionally oscillating genes in the PSM (the molecular clock) and antagonistic signaling gradients along the embryo axis (the wavefront). The molecular oscillator is known as the segmentation clock, which drives cyclic and synchronized gene expression along the PSM.[2] The so-called *clock genes* belong to the Notch, Wnt and fibroblast growth factor (FGF) signaling pathways.[3] The wavefront involves posterior gradients of Wnt and FGF signaling that are counteracted by an opposing gradient of retinoic acid (RA) secreted from the somites.[4] When the segmentation clock reaches cells that have passed the wavefront, segmentation genes, including *Mesp2*, are activated, leading to the specification of the somite boundary.[5] As well as specifying the somite boundaries, RA signaling suppresses signals that break left-right symmetry, ensuring that somite production is bilaterally symmetric.[6,7] This periodic addition of somites underlies body plan generation in all vertebrates, and the oscillating signals from Notch, Wnt, and FGF pathways are conserved in the PSM of model organisms as diverse as mouse, chicken, and zebrafish.[8]

The specification of somites along the anterior-posterior axis is determined before somitogenesis by Hox gene expression,[9] and the specific combination of Hox genes expressed along the axis establishes the identity of the resulting vertebrae.[10] Somites are further patterned along the dorsoventral and mediolateral axes, giving rise to two somitic derivatives found in all vertebrates: the sclerotome (precursor of vertebral and rib cartilage, tendons, and blood vessels) and the dermomyotome (precursor of skeletal muscles and back dermis). Fate specification to either derivative is controlled by signals from adjacent tissues. Ventral cells of the somite differentiate into sclerotome under the

This is an open access article under the CC BY license (http://creativecommons.org/licenses/by/4.0/).

influence of Shh signals from the notochord and the floor-plate of the neural tube. Dorsal cells instead receive Wnt signals from the neural tube and the ectoderm and BMP4 from the lateral mesoderm, to give rise to the dermomyotome.[11]

Master transcriptional regulators driving somite differentiation have been identified through classical genetic approaches.[12,13] However, how these master regulators orchestrate somitogenesis through embryonic space and time, and indeed what genes they directly regulate, remains less clear. While several studies have used gene expression microarrays to characterize gene expression patterns during somitogenesis, these studies have all been performed in the PSM.[8,14,15]

Here, we map the transcriptional and chromatin changes that occur across somite maturation by performing high-resolution RNA and ATAC sequencing of individual, manually microdissected somites at six developmental stages. By comparing the three most recently segmented somites, we characterized the molecular basis of the earliest stages of somite maturation. Additionally, we identified patterns of dynamic regulatory activity across development, with pronounced differences between somites that give rise to differing types of vertebrae. By characterizing the biological processes dominating each stage, we found that somite differentiation accelerates with developmental progression. Finally, we used the combined information from the transcriptional and chromatin maps to define regulatory modules with differing activity during early and late development. These molecular programs control the onset of differentiation, thus regulating the timing of skeletal system development. All data can be explored interactively at https://crukci.shinyapps.io/somitogenesis/.

RESULTS

A high-resolution transcriptional and regulatory map of somite maturation

To characterize the transcriptional changes that orchestrate mouse somite maturation, we generated coupled transcriptional and chromatin accessibility profiles of individual somite pairs, across embryonic development. Each somite typically contains 500–1,000 cells, which is sufficient to generate high-resolution small bulk data. We first compared the transcriptomes of matched left and right somites dissected from 20 to 25 somite embryos and observed no significant differences in expression (Figure S1A), indicating that from a molecular genetics perspective, the two somites were indistinguishable. Therefore, for each somite pair, we used one somite to map the transcriptome (RNA sequencing [RNA-seq]) and one to map matched open chromatin (ATAC-seq) (Figure 1A).

After segmentation, somites maintain a round shape for several hours before undergoing an epithelial-to-mesenchymal transition (EMT) when cells commit to somite-derived lineages and initiate migration.[12] To study the molecular changes associated with fate commitment, we collected the three most posterior pairs of somites, which correspond to those most recently segmented, and that have not yet begun EMT[13,16] (Figure 1B). To understand how somite maturation progresses across embryonic development, we sampled these somite trios from embryos at six different developmental stages. We defined the embryonic stage by counting the total number of somite pairs and profiled at least four different embryos containing 8, 18, 21, 25, 27, and 35 pairs of somites (Figure 1B; Table S1). These stages span four of the five different types of vertebrae (cervical, thoracic, lumbar, and sacral; Figure 1A), providing profiles of somites that will contribute to all four structures.

We generated matched transcriptome (RNA-seq) and open chromatin (ATAC-seq) maps from the vast majority (71/81) of the samples (Table S1). From the 77 RNA-seq libraries, all but one produced good-quality transcriptomes (Tables S2). We normalized for sequencing depth and corrected for batch effects associated with the date of somite collection (STAR Methods; Figures S1B and S1C).

Somites from specific axial levels express particular combinations of Hox genes,[10] which are directly associated with segment identity.[17,18] Somites from different embryonic stages consistently showed clear differences in the class and expression level of Hox genes (Figure 1C), and the expression of Hox genes alone accurately ordered samples according to our observed somite stage (Figure 1D).

ATAC-seq libraries were successfully produced from 75 samples, but 25 of these were removed after applying stringent quality control criteria (Figure S2; Table S3). The remaining 50 open chromatin maps showed efficiency biases, which were correlated with mean fragment abundance (Figures S3A and S3B). To compensate for this trend, we used a loess-based normalization strategy (Figure S3C). Additionally, we applied the same batch correction approach that was used on the RNA-seq data to remove technical variation (Figures S3D and S3E).

We classified the possible functional role of open chromatin regions based on their genomic location. Peaks that were within 200 bp of an annotated transcription start site were deemed promoter-like elements and represent 19% of total peaks; an additional 12% of peaks overlapped gene exons. The remainder of the peaks were annotated as enhancer-like elements and subdivided into *proximal* (24%) or *distal* (7%) if they were within 25 and 100 kb of an annotated gene, respectively, or *intergenic* (1%) (Figure 1E).

Epithelial somites deploy a shared maturation program across embryonic development

We systematically profiled the three most recently segmented somites, which are at the beginning of the differentiation process that will give rise to all somitic derivatives, including muscle, bone, cartilage, and dermis.[19–22] Following the nomenclature proposed by Christ and Ordahl,[23] we refer to each somite in these trios as somites I–III, from the most posterior to the most anterior, respectively (Figure 2A).

To characterize the molecular changes underlying somite maturation, we compared all pairwise combinations of somites I, II, and III at each developmental stage. We identified a median of 453 genes that significantly differ per stage (false discovery rate [FDR] < 5% and |fold-change| > 1.5). Most differentially expressed genes had subtle changes in expression, with half showing less than a 2-fold difference between any two somites. To increase statistical power, we repeated the analysis using samples from different stages as replicates and detected genes that showed consistent changes regardless of developmental stage. Altogether, we identified 2,977 significantly differentially expressed genes. Similar numbers of genes were up- and

CellPress
OPEN ACCESS

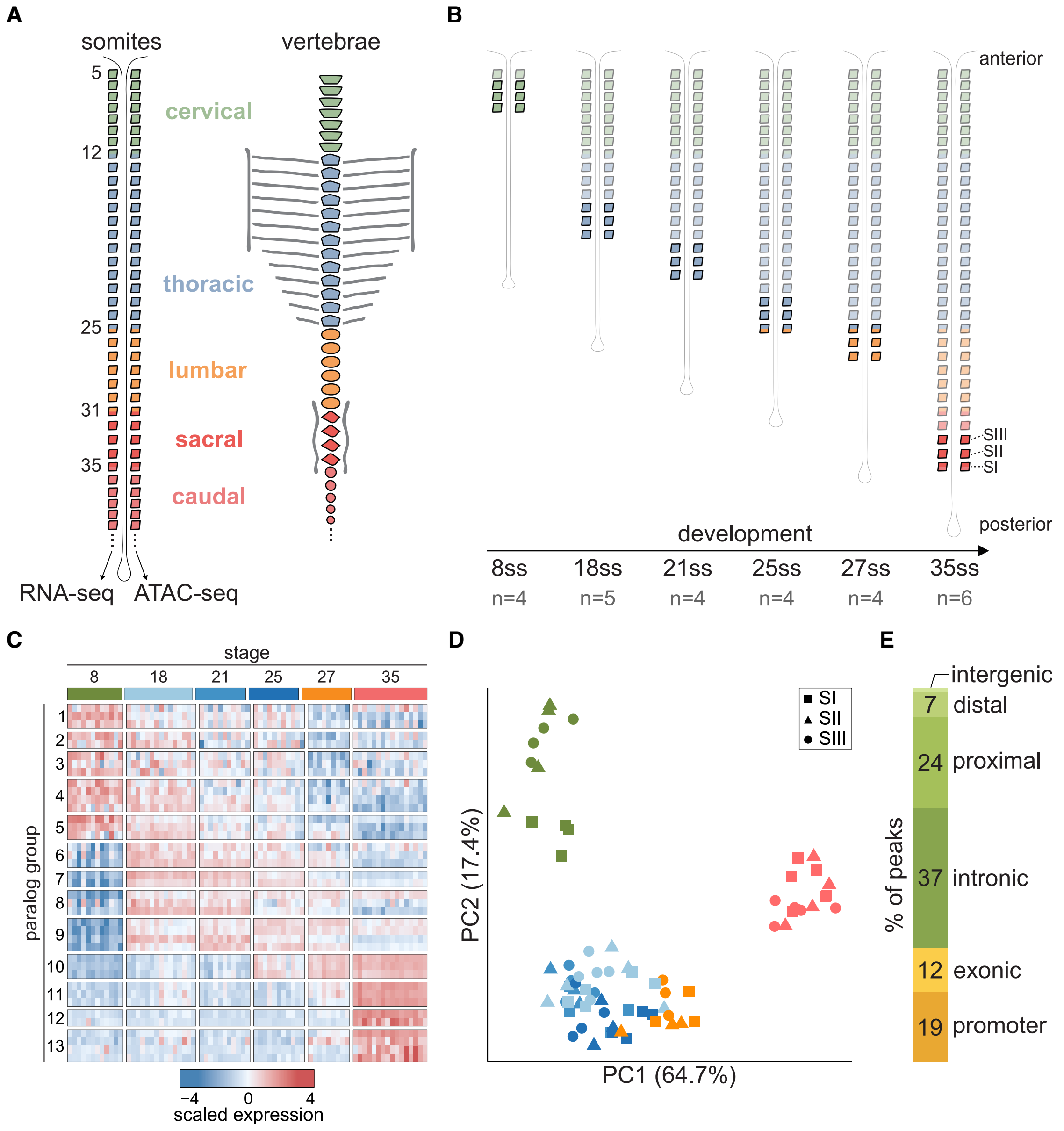

Figure 1. Expression and chromatin profiling of mouse somite maturation

(A) Schematic of somite pairs on each side of the neural tube, and the corresponding vertebrae structures they will form. One somite from each pair was used for RNA-seq, and the other for ATAC-seq. Somites and vertebrae are colored based on their vertebral identity (cervical, thoracic, lumbar, sacral, or caudal). Somites at boundaries between two different types of vertebrae are numbered. The first four somite pairs (occipital) are not shown.

(B) Somites collected in this study. From each embryo, the three most posterior somites (SI–SIII) were collected. Embryos profiled were from six different developmental stages, determined by the number of somites. ss, somite stage; n, number of embryos collected.

(C) Heatmap of the expression of all Hox genes. Samples are ordered in columns according to their observed somite stage. Each row is a different Hox gene, ordered by paralogous groups from 1 to 13. Expression is represented as Z scores.

(D) Principal component analysis of the expression of Hox genes orders somites consistently with their observed somite stage.

(E) Proportion of open chromatin regions classified based on their genomic context.

downregulated, with the strongest differences manifested between somites I and III (Figure 2B). The vast majority of differentially expressed genes (75.8%) showed consistent expression dynamics across different stages. However, most genes (86.9%) also showed differences in expression levels across developmental time, illustrating the complex regulatory dynamics prevalent during embryonic development (Figure 2C).

The genes downregulated along somite maturation were enriched for biological processes related to regionalization and pattern specification, which are active in the PSM and lead to somite segmentation. Consistently, both the Wnt and Notch signaling pathways were preferentially downregulated[3] (Figure 2D). In contrast, a steady progression toward EMT during somite maturation was reflected by the upregulation of cell

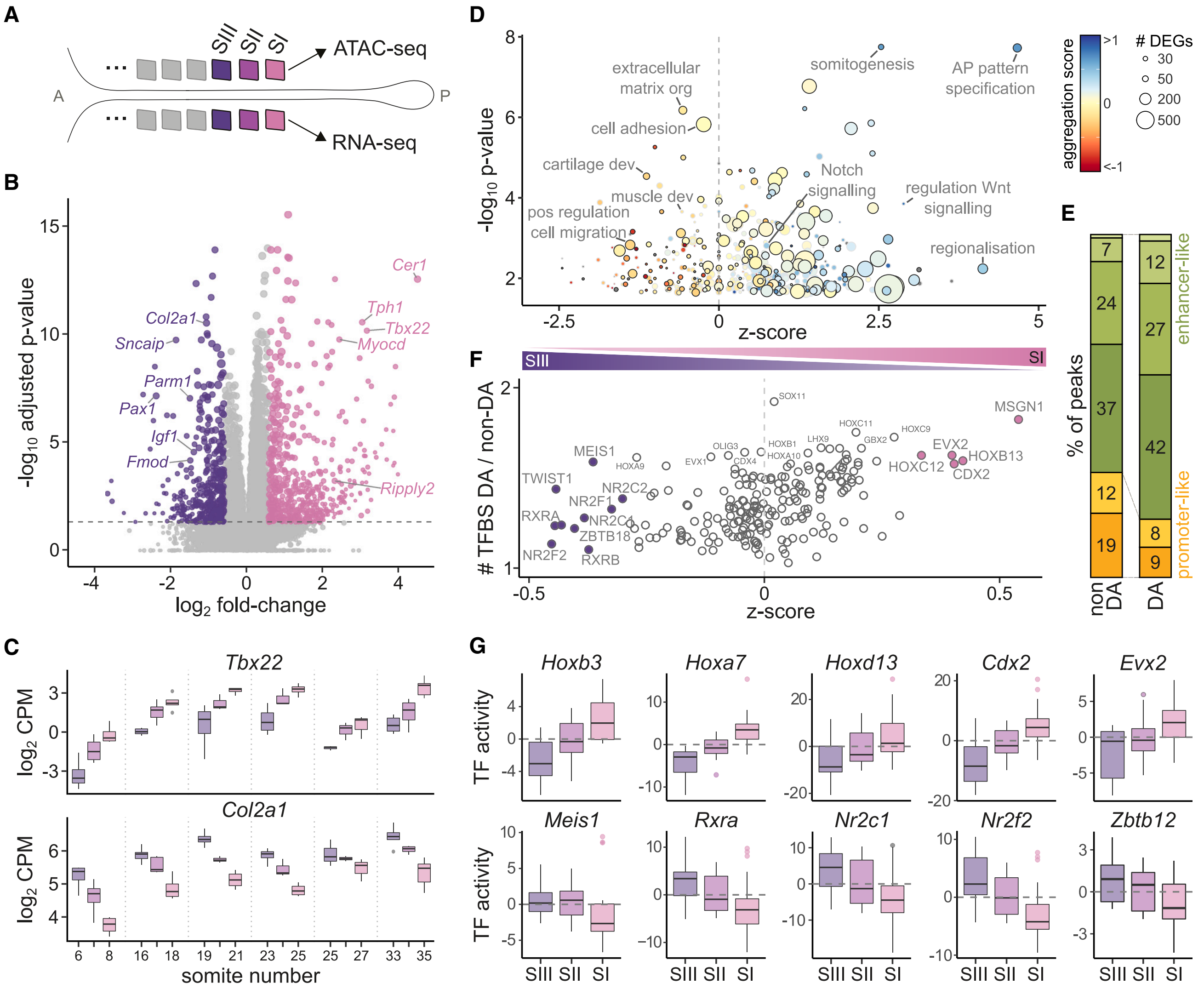

Figure 2. Somites follow a conserved maturation program across development

(A) Schematic indicating the somite trios profiled from each embryo. A, anterior; P, posterior. Color and shading scheme is preserved throughout all figures to indicate the different somites.

(B) Volcano plot of expression changes between somites I and III. Genes significantly differentially expressed are colored.

(C) Differences in gene expression for two representative genes (*Tbx22* and *Col2a1*) among somites I, II, and III are consistently maintained across developmental stages.

(D) Significantly enriched Gene Ontology functional categories in the set of differentially expressed genes. Enrichment significance is shown on the y axis. The x axis indicates whether a term contains a majority of genes that are downregulated (positive) or upregulated (negative) as somites mature. Points are colored based on an "aggregation score," which corresponds to the average fold-change of all differentially expressed genes in the GO term. The size of the points indicates the number of differentially expressed genes in each term. Outlined points correspond to terms that are also significantly enriched in the set of differentially accessible chromatin regions.

(E) Barplot of the proportion of peaks falling in different genomic contexts. Differentially accessible (DA) regions between somites I, II, and III are enriched for enhancers. Colors indicate the same classes as in Figure 1E.

(F) Similar to (D) but showing the enrichment of transcription factor binding sites (TFBSs) in differentially accessible peaks.

(G) Representative examples of motif activity dynamics for TFs that are enriched in differentially accessible peaks. Positive (negative) activity scores indicate the regions are more (less) accessible compared with background chromatin. Hox and other hoemeodomain TF-binding sites close in mature somites, while C4 zinc-finger class of receptors (*Rxra*, *Nr2c1*, *Nr2f2*, and *Zbtb12*) sites become more accessible in SIII.

adhesion and migration programs, together with a switch to positive regulation of Rho and ERK signaling (Figure 2D).

The open chromatin landscape was similarly dynamic across the somite trios, with 2,701 genomic regions showing significantly different accessibility levels (FDR < 5% and |fold-change| > 1.5). Open chromatin regions that actively changed between somites were enriched for enhancer-like regions, with fewer promoter elements (Figure 2E). Indeed, only 506 (18.7%) differentially accessible regions were located within 5 kb of a differentially expressed gene, indicating that the regulatory mechanisms driving

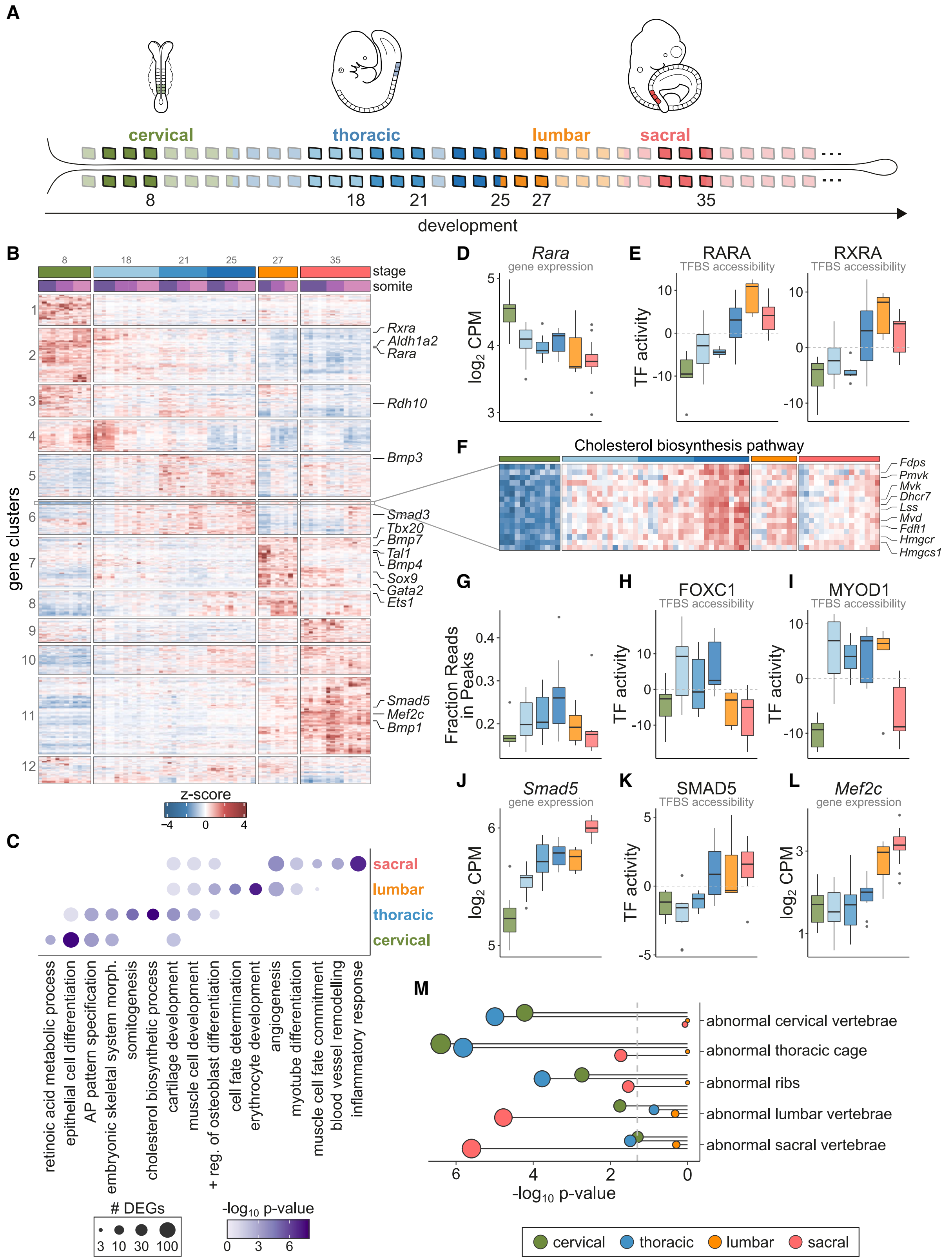

Figure 3. Epithelial somites at late development activate differentiation programs of derivative lineages absent in early stages

(A) Schematic indicating the somites profiled across development, and their vertebral fate. Color scheme is preserved throughout all figures to indicate the different stages.

(legend continued on next page)

expression changes operate through distal regulatory elements, rather than by directly modulating chromatin accessibility at promoters. The coordination of dynamic chromatin accessibility and gene expression changes was also reflected in their shared overrepresentation of the same biological functions (Figure 2D).

Next, we annotated transcription factor (TF)-binding motifs within ATAC-seq peaks and identified 201 regulators whose binding motifs were significantly enriched in the dynamic regions, when compared with static open chromatin (Figure 2F). These included Hox factors, as well as multiple members of the homeodomain, Tal, Sox, and NK families. For example, binding motifs for MSGN1 were present in 22% of all dynamic regions (compared with 12% in non-differentially accessible chromatin), and most of these peaks showed reduced accessibility in more mature somites, consistent with the role of this protein as a master regulator of PSM differentiation[24] (Figure 2F). In contrast, the dynamic peaks with binding motifs for TWIST1, a critical factor mediating EMT, were more accessible in the most mature somites[25] (Figure 2F).

Finally, to understand how the overrepresented TFs regulate somite maturation, we analyzed the accessibility dynamics of the genomic loci with binding sites for each of these TFs. Binding sites for all Hox proteins, regardless of their paralogous group or stage activity pattern, showed decreased accessibility upon somite maturation (Figure 2G). This behavior also extended to most of the other TFs enriched in differentially accessible peaks (Figure 2G). One notable exception gained accessibility as somites matured: the C4 zinc-finger class of receptors, which includes the retinoid-X-receptor-related factors, critical in mediating the biological effects of retinoid signaling and its differentiation-inducing activity[26] (Figure 2G).

Molecular remodeling across development regulates somite responses to the signaling environment

Our data identified profound changes in transcriptional and regulatory activity in somites I–III across development. We compared the RNA-seq profiles of somites among all different developmental stages (Figure 3A) and identified 10,691 genes with significant changes in expression (FDR < 5% and |fold-change| > 1.5; see STAR Methods for details; Figure 3B), including most known TFs (838 from a total of 1,310 expressed). The chromatin landscape was also remodeled extensively, with 33,013 open chromatin regions showing significant differences in accessibility (Figure S4A). In contrast to the changes observed across somite maturation, a much higher proportion of differentially accessible loci were in promoters or close to differentially expressed genes (Figure S4B), indicating that across development, widespread chromatin remodeling plays a crucial role in controlling the genes available for expression.

When ordered by developmental stage, somites from different vertebral fates showed waves of temporally restricted transcriptional and chromatin remodeling (Figures 3B and S4A). We analyzed these patterns of coordinated gene expression by performing enrichment analysis of gene ontology (GO) functional terms. Differentially expressed genes with highest expression in cervical somites (clusters 1–4) were related to epithelial cell development and response to RA signaling, including several RA receptors (Figures 3B and 3C). Additional genes involved in somitogenesis and embryonic patterning were prevalent in both cervical and thoracic somites (clusters 1–6, Figure 3C) and were generally expressed at the highest level in the most recently segmented somite. This suggests that somites at these early developmental stages closely resemble their PSM lineage and are only beginning to activate a somite-specific transcriptional profile.

We used our data to dissect the complex interplay between metabolite production, TF activity and chromatin dynamics involved in RA signaling, which requires precise spatiotemporal regulation for adequate differentiation of progenitor cells.[26] RA signaling effects are mediated by the RA receptor (RAR) and retinoid X receptor (RXR) families. These ligand-dependent TFs can recruit either corepressors or coactivators to induce changes in chromatin condensation and regulate transcription.[26] Expression of the enzymes involved in RA production (*Aldh1a2* and *Rdh10*) as well as of several RAR/RXR TFs peaked early in development (Figures 3B and 3D). However, the accessibility of loci with binding sites for RAR/RXR factors was lowest at this stage (Figure 3E), suggesting their association with corepressors to induce chromatin condensation. As development proceeded these chromatin loci progressively increased in accessibility, maintaining an open chromatin configuration from stage 25 onward (Figure 3E). These data indicate that the epigenetic profile of somites is reshaped across development from a repressive to a permissive state for RA signaling activity.

Thoracic somites showed strong enrichment for genes involved in the development of the skeletal system, including both the muscle and cartilage lineages (clusters 5 and 6; Figure 3C). We observed prominent expression of many

(B) Heatmap of expression of genes differentially expressed across development. Samples (columns) are ordered based on their somite number, and their stage and somite level are indicated at the top. Differentially expressed genes are grouped into clusters by hierarchical clustering.

(C) Gene ontology term enrichment analysis results for sets of genes with highest activity at particular vertebral fates. The size of the circles indicates the number of differentially expressed genes in each term-fate combination, and the intensity of the color corresponds to the significance of the enrichment.

(D) Expression of the retinoic acid receptor gene *Rara* across development. Related factors such as *Rxra* show a similar pattern.

(E) Chromatin activity scores from chromVAR for the genome-wide binding sites (TFBSs) of RARA and RXRA. Positive (negative) scores indicate higher (lower) accessibility than background chromatin.

(F) Zoomed-in region of the heatmap in (B), showing the expression of genes in the cholesterol biosynthesis pathway.

(G) Boxplots of the fraction of reads that are within peaks in each sample.

(H and I) Chromatin activity scores for FOXC1 (H) and MYOD1 (I), as in (E).

(J and K) *Smad5* gene expression (J) and chromatin activity at its TFBSs (K) across stages.

(L) Gene expression levels for *Mef2c* across development.

(M) Significance scores for enrichment of chromatin regions associated with genes that show skeletal abnormalities in knockout (KO) mice. The significance of each term is shown separately for the sets of regions with highest activity at each vertebral fate, indicated by the color of the circle. Circle size is proportional to the significance level. See also Figure S3.

components of the TGF-β, BMP, and Smad signaling pathways, which are fundamental in orchestrating skeletal system development[27] (Figure 3B). We also observed coordinated expression of cholesterol biosynthesis, with maximal expression of 17 metabolically central genes at stage 25 before being downregulated at later stages (Figure 3F). Among several functions, cholesterol plays an important role in the transduction of hedgehog signaling[28,29] and is required for the correct development of muscle and bone.[30,31] Sonic hedgehog (SHH) is secreted by the notochord and controls the specification of the sclerotome during patterning of epithelial somites.[32] Defective cholesterol biosynthesis leads to impaired response to Shh signaling and skeletal defects.[28,29] As expected, we did not detect significant *Shh* expression in the somites. However, we suggest that the tightly controlled expression of the cholesterol pathway components could be a mechanism to control when somites are most responsive to extrinsic hedgehog signaling.

Somite differentiation accelerates across development

Our data identified that chromatin remodeling is concentrated in thoracic somites. In addition to activating the gene programs controlling skeletal system development, somites at stages 18–25 generally showed higher levels of open chromatin, compared with other stages. We observed a sharp increase in the fraction of reads in peaks in somites from stage 18 embryos, with further increases at stages 21 and 25, before dropping in the stage 27 somites (Figure 3G). Consistently, over half of all differentially accessible chromatin loci showed highest accessibility in thoracic somites (Figure S4A). These chromatin loci were enriched for many TF motifs, including several with well described roles in skeletal system development such as forkhead TFs, implicated in both skeletal muscle and cartilage development[33,34] (Figure 3H). We also observed increased accessibility at loci harboring binding sites for MYOD1 (Figure 3I) and MYF5 (Figure S4C), which are essential for cell commitment to the myogenic lineage.[35]

Previous work[36–39] have characterized the expression dynamics of sclerotome and myotome markers, including *Myod1*, at several embryonic stages. These studies showed that somites from younger embryos take longer to activate marker gene expression compared with somites from more advanced embryos. We hypothesized that the shorter times required for marker expression onset in late development stem from a change in the permissiveness of the chromatin landscape, which allows lineage-defining TFs to activate their downstream pathways sooner. Analysis of the active biological processes prevalent at later developmental stages indeed showed a switch from cell development and morphogenesis programs to lineage commitment and differentiation (Figure 3C). These transitions were often accompanied by shifts in the active components of canonical signaling pathways, both by altering the expression of key TFs and by changing the permissiveness of the chromatin at their effector sites throughout the genome. For example, while expression of *Smad3* and *Bmp3* was at its highest levels in thoracic somites, increasing expression of *Smad5* alongside *Bmp1*, *Bmp4*, and *Bmp7* was observed later in development (Figure 3B). *Smad5* was expressed at all stages, albeit at lower levels early on (Figure 3J); however, its binding sites only became accessible from stage 25 onward, when expression was highest (Figure 3K). Signaling through SMAD2/3 and SMAD1/5 have opposing effects on differentiation. For example, while BMP3-SMAD3 block osteogenesis, BMP1 and BMP7, downstream of TGF-β, promote osteoblast production.[27] Thus, the switch in usage of the opposing arms of the SMAD-BMP or SMAD-TGF-β signaling pathways suggests that cells at later developmental stages have progressed further in their differentiation trajectory. Consistent with this, genes crucial for fate determination and commitment also increased in expression across time: *Mef2c* (Figure 3L), which is fundamental in myogenic differentiation, and *Sox9* (Figure S4D), which specifies chondrocytes, peaked in sacral somites.[40,41]

Additionally, we observed significant upregulation of *Bmp4* specifically in lumbar somites (cluster 7, Figure 3B). Besides regulating the development of the skeletal system, BMP4 has been shown to induce the expression of *Flk1* (*Kdr*) in epithelial somites,[42] a factor essential for vasculogenesis and angiogenesis. The dermomyotome derivative lineages include vascular endothelial cells. Concomitant with *Bmp4* upregulation, we also observed increased expression of many other genes involved in angiogenesis, such as *Gata2*, *Tal1*, *Ets1*, and *Tbx20* (cluster 7, Figure 3B). Later in development, sacral somites continued to express high levels of angiogenic factors and further activated more mature programs involved in blood vessel remodeling (Figure 3C).

Finally, to assess whether our molecular atlas captures regulators important in determining vertebral fate identity, we tested for enrichment of genes with specific mouse knockout phenotypes. Genomic loci with highest activity in cervical or thoracic somites were strongly enriched for phenotypes affecting cervical vertebrae and thoracic and rib morphology. In contrast, regions active in sacral somites were associated with abnormal lumbar and sacral vertebrae (Figure 3M). Thus, our catalog of differential activity along the axial skeleton can be utilized to identify genes and regulatory elements important for the specification of the different vertebral structures.

Skeletogenesis is shaped by opposing regulatory modules

Next, we leveraged the paired design of our dataset to map chromatin-transcription regulatory interactions driving somite maturation and differentiation, by applying the functional inference of gene regulation (FigR) method[43] (with minor modifications, see STAR Methods for details). First, we computed the correlation between the activity levels of all differentially expressed genes and the open chromatin peaks within 100 kb to identify regulatory elements likely to direct nearby gene expression changes. Peak-gene pairs were considered significantly associated if they had stronger correlation values compared with randomized interactions. After restricting results to pairs with moderate to high correlation scores (>0.3), we identified 12,803 putative regulatory interactions, involving 47% of all differentially expressed genes. Although a small fraction of the interactions included promoter-like peaks in the immediate vicinity of the genes, most resembled enhancers and were dozens of kilobases away, with a median distance of 38 kb (interquartile range: 13.1–67.5 kb). Linked peaks overlapped more often with FANTOM5 and ENCODE enhancers (H3K27ac-high, H3K4me3-low signature) compared with all peaks, lending

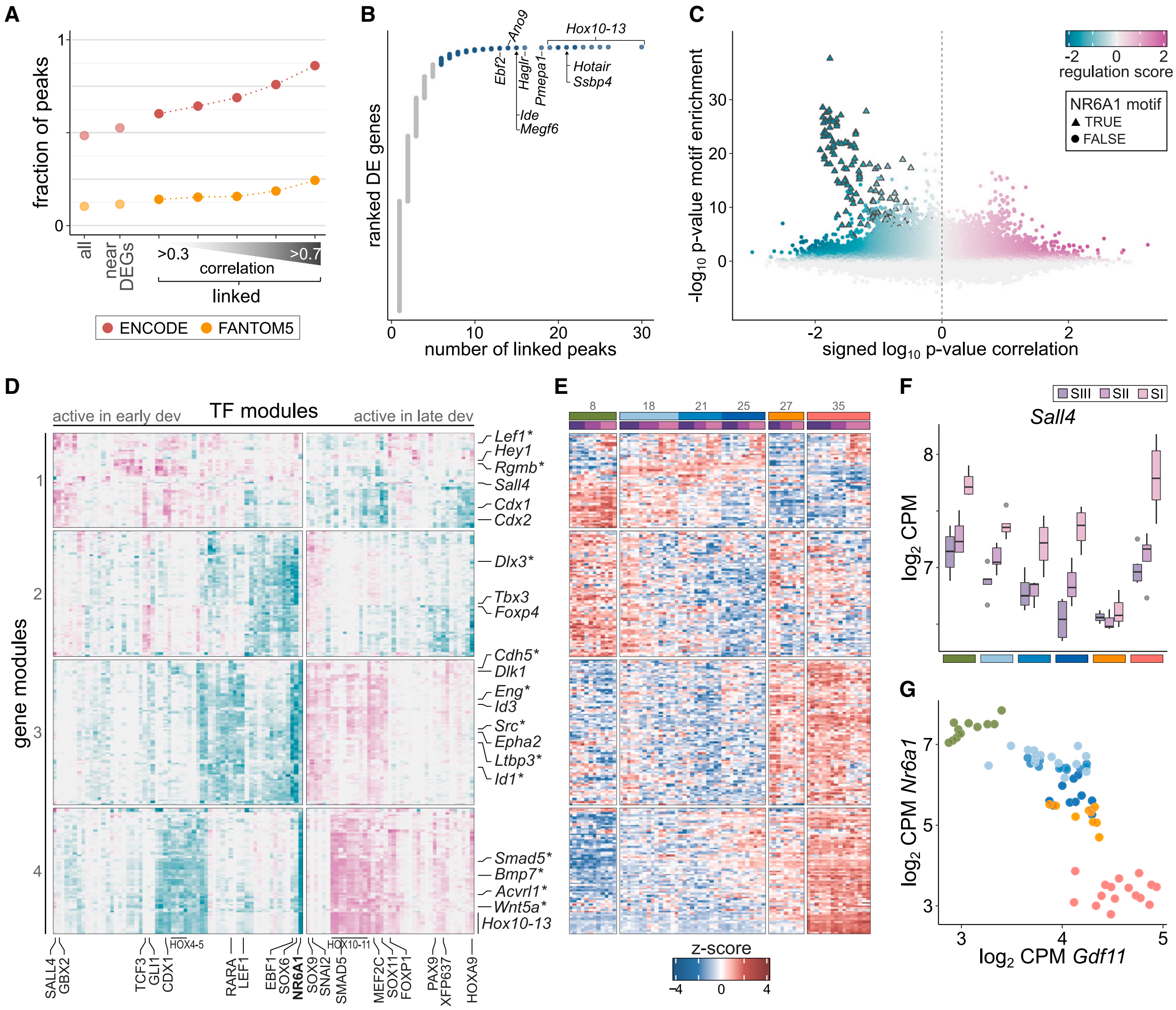

Figure 4. Regulatory modules with opposing activity along embryonic development control timely activation of skeletogenesis pathways

(A) Fraction of peaks that overlap enhancer elements from the ENCODE (red) and FANTOM5 (orange) catalogs. Peaks identified as putative regulators of differentially expressed genes (linked peaks) are more likely to be annotated enhancers. This fraction increases as the set of peaks is restricted to stronger interactions, as shown by limiting to linked peaks with correlation scores higher than 0.3–0.7.

(B) For each differentially expressed (DE) gene, the number of significantly associated peaks within 100 kb. Several hundred genes are linked to a large number of peaks, and these include many late Hox genes.

(C) Regulation scores predicted by FigR between transcription factors (TFs) and genes with many linked peaks (blue set from B). The x axis indicates the strength of the correlation between TF expression and peak accessibility; the y axis corresponds to the significance of the enrichment of the TF-binding sites in the linked peaks. Interactions involving NR6A1 are highlighted with triangles.

(D) Heatmap depicting patterns of regulatory activity between TFs (columns) and genes (rows). Genes are split into four modules by hierarchical clustering. Genes from the TGF-β and BMP signaling pathways are highlighted with asterisks. Color scale is the same as in (C).

(E) Heatmap showing the expression levels of the same genes as in (D) across all somites profiled in this study. Samples (columns) are ordered based on their observed somite stage (indicated at the top).

(F) Expression levels of *Sall4*, one of the TFs with large regulation scores on module 1 genes.

(G) Expression levels of *Nr6a1* and *Gdf11* in all somites show a strong antagonistic relationship.

support for their regulatory activity (Figure 4A). This proportion sharply increased when links were restricted to those with the strongest correlations (Figure 4A). Thus, this strategy serves to enrich the set of chromatin loci for enhancer elements, and to associate their activity to dynamically regulated genes.

Most (93.7%) differentially expressed genes significantly linked with chromatin changes were associated with one to five putative enhancer regions, but a few hundred genes were linked to many more enhancers (Figure 4B). The set of 349 strongly connected genes contained factors key in controlling

somite development and differentiation, suggesting that these processes are under complex regulatory control. Some of the most highly connected genes were Hox factors from late paralogous groups (Figure 4B), consistent with chromatin remodeling playing a crucial role in controlling their timely expression.[44] Next, we scanned the peaks associated with these highly regulated genes and identified enriched TF motifs. TF-gene pairs were assigned a *regulation score* that favors TFs showing correlated expression to the accessibility dynamics of linked peaks (Figure 4C).

We identified opposing transcriptional programs active in early and late development by clustering TFs with large regulation scores (Figures 4D and 4E). Four different modules of regulatory activity were evident. Module 1 acted on genes that show differences in expression between the somite trios, including genes involved in the establishment of anterior-posterior patterning and somitogenesis, such as *Cdx1/2*, *Gbx2*, *Lef1*, and Hox genes from early paralogous groups (Figures 4D and 4E). CDX1 and GBX2 themselves, together with SALL4, showed some of the strongest regulation scores on these genes. Consistent with their role in the specification and patterning of somitic mesoderm, their expression was highest in the most immature somite I (Figure 4F).

The other three modules were instead related to genes that are downregulated (module 2) or upregulated (modules 3 and 4) with developmental progression (Figure 4E). Module 2 activity was influenced by Shh signaling (GLI1; Figure 4D), while genes expressed late in development were under the control of several TFs. Among these, NR6A1 showed a prominent role, particularly in module 4, with its binding sites highly enriched in the peaks linked to these genes (Figure 4C). Expression of *Nr6a1* was negatively correlated with peak accessibility, indicating a repressive regulatory effect. A number of other TFs, including late Hox TFs, showed large positive regulation scores on the same genes (Figure 4D), suggesting antagonistic regulatory activities to NR6A1. Among this set of activating TFs were factors key in the specification of the muscle and cartilage lineages (MEF2C and SOX9), as well as proteins involved in balancing proliferation and differentiation of the progenitor cells, with many required to avoid premature differentiation (SNAI2, ZFP637, HOXA9, and FOXP1; Figure 4D).

Recently, NR6A1 was shown to be a key regulator of the trunk-to-tail transition in the tailbud, where its expression early in development prevents premature activation of late-expressing genes, including late Hox genes. *Nr6a1* expression is then terminated by *Gdf11* to allow the trunk-to-tail transition.[45] Although these regulatory interactions were dissected in undifferentiated tailbud mesoderm cells, we observed the antagonistic expression between *Nr6a1* and *Gdf11* is retained in segmented somites (Figure 4G), suggesting this regulatory program remains at play as cells commit to the somitic lineage. Consistently, FigR predicted the strongest effects exerted by NR6A1 to affect all Hox genes in paralogous groups 10 to 13 (Figure 4D). Additional predicted regulatory interactions included several components of the TGF-β and BMP signaling pathways from modules 3 and 4 (highlighted with asterisks in Figure 4D), with the peaks associated with these genes also showing significant enrichment for NR6A1 binding motifs. Among these genes was *Smad5*, with a regulation score only slightly lower than those observed for late Hox genes. Further, SMAD5 itself was identified as a positive regulator of module 4 genes (Figure 4D). Based on these results, we speculate that the regulatory network controlled by NR6A1 not only regulates the trunk-to-tail transition in paraxial mesoderm, but it may participate in the timely activation of differentiation pathways required for skeletogenesis. As *Nr6a1* expression diminishes in later development, it is possible that its repression of TGF-β and BMP signaling lessens. In turn, other TFs would be able to increase in activity to enhance these pathways and drive the commitment and differentiation of cells down the various somitic derivative lineages.

DISCUSSION

The establishment of the vertebrate body plan through somitogenesis is deeply conserved. Although the molecular mechanisms driving the segmentation process are shared, alterations to the number and class of segments between different species allows facile generation of vastly different body structures among vertebrates.[46] Previous studies have characterized the transcriptional changes accompanying the transition from unsegmented mesoderm to nascent and differentiated somites in the chick,[47] mouse,[48] and human embryos,[49] at a single developmental stage. These studies have provided insights into the molecular pathways controlling segmentation and the subsequent differentiation of somitic mesoderm derivatives.

Here, we have experimentally analyzed how the three most recently segmented somites of mouse embryos remodel their transcriptional and chromatin landscapes at six different developmental stages, capturing the earliest molecular mechanisms that give rise to cervical, thoracic, lumbar, and sacral structures. We identified three thousand genes transcriptionally remodeled during somite maturation. The expression of these genes after segmentation follows the same pattern at independent stages, indicating that somites from different axial levels adhere to a conserved differentiation trajectory. However, we also identified genes expressed in specific developmental stages of the embryo, reflecting changes in the microenvironment in which somites develop.

To increase the accessibility of our data and support the generation of novel hypotheses, we have created an interactive website (https://crukci.shinyapps.io/somitogenesis/) where the expression of any gene can be evaluated during the somite time course. For example, *Pax1* and *Pax9* are important regulators of sclerotome proliferation and differentiation.[50] Consistently, our data show a clear upregulation of both genes as somites mature, and this is accompanied by an overall increase in expression levels during later developmental stages. Furthermore, several downstream targets of *Pax1* and *Pax9* have been identified, including *Col2a1*, *Sox5*, and *Wwp2*.[50] When interrogating their expression patterns in our somite profiles, we observe that the expression of *Col2a1* is strongly correlated with *Pax1* (Pearson r = 0.85), while it shows a more modest relationship with *Pax9* (Pearson r = 0.69). In contrast, the opposite pattern is observed for *Sox5* (*Pax9* Pearson r = 0.67; *Pax1* Pearson r = 0.55). Thus, our data can be exploited to help decipher the regulatory programs underpinning somite maturation.

Our data show that somite differentiation accelerates as embryos grow. We observed that somites from stage 8 embryos maintain a naive transcriptional profile for the entirety of the three segmentation clock cycles captured in our data, but progression along development results in a shortening of the time spent in such an undifferentiated state. At later stages, somitic gene regulation is dominated by TFs controlling cell fate commitment. By the time embryos have formed 35 pairs of somites, differentiation programs of derivative lineages are upregulated within a few hours post-segmentation and can already be observed in somite III. This is consistent with previous work in chick embryos[36–39] that showed pronounced differences in the onset of expression of key factors for the commitment of cells to the myogenic lineage, depending on embryonic age. Our results provide evidence that this is also a feature of mouse embryos.

By integrating our transcriptional and epigenetic datasets, it is possible to start dissecting the regulatory mechanisms controlling the differentiation processes described above. However, several limitations warrant consideration when interpreting these results. First, early and late somites differ in the size and number of cells present at the time of segmentation,[51] meaning that changes in expression or accessibility can be the result of compositional differences and/or changes in the activity within individual cells. Second, the segmentation clock slows down as development proceeds,[51] and thus, somite trios from later stages span a longer time window than those from earlier embryos, increasing the time for these somites to activate lineage determining pathways. Finally, while we have characterized the molecular changes occurring within the somites, somites develop concomitantly with other organ systems. The complexity of the signaling microenvironment and functional processes active across organogenesis differ substantially between embryos from cervical versus sacral stages. Integrating our data with recently generated datasets that profile mouse embryos from different somite stages at single-cell resolution[52] will potentially allow to address some of these questions.

Furthermore, we characterize the regulatory mechanisms controlling cell fate specification and commitment well before the onset of definitive lineage markers, showing that the acceleration of somite differentiation in later development initiates soon after somite segmentation. This phenomenon is not restricted to the myogenic lineage but extends to other cell type populations, including chondrogenic and endothelial cells. In sum, our high-resolution view of the molecular mechanisms underlying the specification and development of somitic lineages has uncovered additional features of somite maturation and is a powerful resource for the developmental biology community to study its progression in mammals.

Limitations of the study

The embryos used in our experiments were carefully staged by the number of segmented somites. However, embryos will be at different stages within the next clock cycle leading to some asynchrony among replicates. Additionally, by using bulk profiling techniques, we have generated the average profile of somites across time, and we do not have any information on cell composition changes. Thus, differences in activity levels between somites could be due to changes in expression regulation and/or abundance of specific cell states.

STAR★METHODS

Detailed methods are provided in the online version of this paper and include the following:

- KEY RESOURCES TABLE
- RESOURCE AVAILABILITY
  - Lead contact
  - Materials availability
  - Data and code availability
- EXPERIMENTAL MODEL AND STUDY PARTICIPANT DETAILS
- METHOD DETAILS
  - Mouse embryo collection and dissection
  - Experimental design
  - RNA-seq experiments and library preparation
  - ATAC-seq experiments and library preparation
- QUANTIFICATION AND STATISTICAL ANALYSIS
  - RNA-seq data processing and quality control
  - RNA-seq data normalization
  - RNA-seq differential expression analysis
  - ATAC-seq data alignment
  - ATAC-seq quality control
  - ATAC-seq peak calling
  - ATAC-seq data normalization
  - ATAC-seq differential accessibility analysis
  - Functional terms enrichment analysis
  - Motif enrichment analysis
  - chromVAR
  - FigR
- ADDITIONAL RESOURCES

SUPPLEMENTAL INFORMATION

Supplemental information can be found online at https://doi.org/10.1016/j.devcel.2023.07.003.

ACKNOWLEDGMENTS

The authors would like to thank Aaron Lun for valuable suggestions on the analytical strategy, Pascale Gilardi-Hebenstreit for insightful comments on the manuscript, and CRUK Cambridge Institute Bioinformatics and Biological Resources Unit Core Facilities. This work was supported by Cancer Research UK (to E.T. and D.T.O.: 20412 and 22398), the Wellcome Trust (to X.I.-S.: 108438/Z/15 to J.C.M. and 202878/Z/16/Z to D.T.O.), the European Molecular Biology Laboratory (to J.C.M.), the European Research Council (to D.T.O.: 615584 and 788937), and Helmholtz NCT funding (D.T.O.: DKFZ Abteiling B270).

AUTHOR CONTRIBUTIONS

E.T., X.I.-S., J.C.M., and D.T.O. designed the study; E.T. performed experiments; A.E.M. and G.F.M. provided microdissection training; X.I.-S. analyzed the data; X.I.-S., J.C.M., and D.T.O. wrote the manuscript; X.I.-S., J.C.M., and D.T.O. oversaw the work. All authors read and approved the final manuscript.

DECLARATION OF INTERESTS

J.C.M. has been an employee of Genentech since September 2022.

Received: January 25, 2023
Revised: May 12, 2023
Accepted: July 4, 2023
Published: July 26, 2023

CellPress
OPEN ACCESS

REFERENCES

1. Cooke, J., and Zeeman, E.C. (1976). A clock and wavefront model for control of the number of repeated structures during animal morphogenesis. J. Theor. Biol. *58*, 455–476. https://doi.org/10.1016/S0022-5193(76)80131-2.
2. Palmeirim, I., Henrique, D., Ish-Horowicz, D., and Pourquié, O. (1997). Avian hairy gene expression identifies a molecular clock linked to vertebrate segmentation and somitogenesis. Cell *91*, 639–648. https://doi.org/10.1016/s0092-8674(00)80451-1.
3. Dequéant, M.L., and Pourquié, O. (2008). Segmental patterning of the vertebrate embryonic axis. Nat. Rev. Genet. *9*, 370–382. https://doi.org/10.1038/nrg2320.
4. Bénazéraf, B., and Pourquié, O. (2013). Formation and segmentation of the vertebrate body axis. Annu. Rev. Cell Dev. Biol. *29*, 1–26. https://doi.org/10.1146/annurev-cellbio-101011-155703.
5. Saga, Y. (2012). The mechanism of somite formation in mice. Curr. Opin. Genet. Dev. *22*, 331–338. https://doi.org/10.1016/j.gde.2012.05.004.
6. Vermot, J., and Pourquié, O. (2005). Retinoic acid coordinates somitogenesis and left-right patterning in vertebrate embryos. Nature *435*, 215–220. https://doi.org/10.1038/nature03488.
7. Vermot, J., Gallego Llamas, J.G., Fraulob, V., Niederreither, K., Chambon, P., and Dollé, P. (2005). Retinoic acid controls the bilateral symmetry of somite formation in the mouse embryo. Science *308*, 563–566. https://doi.org/10.1126/science.1108363.
8. Krol, A.J., Roellig, D., Dequéant, M.L., Tassy, O., Glynn, E., Hattem, G., Mushegian, A., Oates, A.C., and Pourquié, O. (2011). Evolutionary plasticity of segmentation clock networks. Development *138*, 2783–2792. https://doi.org/10.1242/dev.063834.
9. Krumlauf, R. (1994). Hox genes in vertebrate development. Cell *78*, 191–201. https://doi.org/10.1016/0092-8674(94)90290-9.
10. Wellik, D.M. (2007). Hox patterning of the vertebrate axial skeleton. Dev. Dyn. *236*, 2454–2463. https://doi.org/10.1002/dvdy.21286.
11. Weldon, S.A., and Münsterberg, A.E. (2022). Somite development and regionalisation of the vertebral axial skeleton. Semin. Cell Dev. Biol. *127*, 10–16. https://doi.org/10.1016/j.semcdb.2021.10.003.
12. Yusuf, F., and Brand-Saberi, B. (2006). The eventful somite: patterning, fate determination and cell division in the somite. Anat. Embryol. (Berl.) *211* (*Suppl 1*), 21–30. https://doi.org/10.1007/s00429-006-0119-8.
13. Christ, B., Huang, R., and Scaal, M. (2007). Amniote somite derivatives. Dev. Dyn. *236*, 2382–2396. https://doi.org/10.1002/dvdy.21189.
14. Dequéant, M.L., Glynn, E., Gaudenz, K., Wahl, M., Chen, J., Mushegian, A., and Pourquié, O. (2006). A complex oscillating network of signaling genes underlies the mouse segmentation clock. Science *314*, 1595–1598. https://doi.org/10.1126/science.1133141.
15. Ozbudak, E.M., Tassy, O., and Pourquié, O. (2010). Spatiotemporal compartmentalization of key physiological processes during muscle precursor differentiation. Proc. Natl. Acad. Sci. USA *107*, 4224–4229. https://doi.org/10.1073/pnas.0909375107.
16. Jacob, M., Christ, B., and Jacob, H.J. (1975). Regional determination of the paraxial mesoderm in young chick embryos. Verh. Anat. Ges. *69*, 263–269.
17. Kmita, M., and Duboule, D. (2003). Organizing axes in time and space; 25 years of colinear tinkering. Science *301*, 331–333. https://doi.org/10.1126/science.1085753.
18. Mallo, M., Wellik, D.M., and Deschamps, J. (2010). Hox genes and regional patterning of the vertebrate body plan. Dev. Biol. *344*, 7–15. https://doi.org/10.1016/j.ydbio.2010.04.024.
19. Jacob, H.J., Christ, B., and Jacob, M. (1974). Somitogenesis in the chick embryo. Experiments on the position development of the myotome. Verh. Anat. Ges. *68*, 581–589.
20. Christ, B., Brand-Saberi, B., Grim, M., and Wilting, J. (1992). Local signalling in dermomyotomal cell type specification. Anat. Embryol. (Berl.) *186*, 505–510. https://doi.org/10.1007/BF00185464.
21. Ordahl, C.P., and Le Douarin, N.M. (1992). Two myogenic lineages within the developing somite. Development *114*, 339–353. https://doi.org/10.1242/dev.114.2.339.
22. Aoyama, H. (1993). Developmental plasticity of the prospective dermatome and the prospective sclerotome region of an avian somite: (somite/dermomyotome/sclerotome/determination/quail-chick chimera). Dev. Growth Differ. *35*, 507–519. https://doi.org/10.1111/j.1440-169X.1993.00507.x.
23. Christ, B., and Ordahl, C.P. (1995). Early stages of chick somite development. Anat. Embryol. (Berl.) *191*, 381–396. https://doi.org/10.1007/BF00304424.
24. Chalamalasetty, R.B., Garriock, R.J., Dunty, W.C., Jr., Kennedy, M.W., Jailwala, P., Si, H., and Yamaguchi, T.P. (2014). Mesogenin 1 is a master regulator of paraxial presomitic mesoderm differentiation. Development *141*, 4285–4297. https://doi.org/10.1242/dev.110908.
25. Lamouille, S., Xu, J., and Derynck, R. (2014). Molecular mechanisms of epithelial-mesenchymal transition. Nat. Rev. Mol. Cell Biol. *15*, 178–196. https://doi.org/10.1038/nrm3758.
26. Draut, H., Liebenstein, T., and Begemann, G. (2019). New insights into the control of cell fate choices and differentiation by retinoic acid in cranial, axial and caudal structures. Biomolecules *9*. https://doi.org/10.3390/biom9120860.
27. Wu, M., Chen, G., and Li, Y.P. (2016). TGF-β and BMP signaling in osteoblast, skeletal development, and bone formation, homeostasis and disease. Bone Res. *4*, 16009. https://doi.org/10.1038/boneres.2016.9.
28. Xu, S., and Tang, C. (2022). Cholesterol and hedgehog signaling: mutual regulation and beyond. Front. Cell Dev. Biol. *10*, 774291. https://doi.org/10.3389/fcell.2022.774291.
29. Stottmann, R.W., Turbe-Doan, A., Tran, P., Kratz, L.E., Moran, J.L., Kelley, R.I., and Beier, D.R. (2011). Cholesterol metabolism is required for intracellular hedgehog signal transduction in vivo. PLoS Genet. *7*, e1002224. https://doi.org/10.1371/journal.pgen.1002224.
30. Campos, L.M., Rios, E.A., Midlej, V., Atella, G.C., Herculano-Houzel, S., Benchimol, M., Mermelstein, C., and Costa, M.L. (2015). Structural analysis of alterations in zebrafish muscle differentiation induced by simvastatin and their recovery with cholesterol. J. Histochem. Cytochem. *63*, 427–437. https://doi.org/10.1369/0022155415580396.
31. Anderson, R.A., Schwalbach, K.T., Mui, S.R., LeClair, E.E., Topczewska, J.M., and Topczewski, J. (2020). Zebrafish models of skeletal dysplasia induced by cholesterol biosynthesis deficiency. Dis. Model. Mech. *13*. https://doi.org/10.1242/dmm.042549.
32. Murtaugh, L.C., Chyung, J.H., and Lassar, A.B. (1999). Sonic hedgehog promotes somitic chondrogenesis by altering the cellular response to BMP signaling. Genes Dev. *13*, 225–237. https://doi.org/10.1101/gad.13.2.225.
33. Sanchez, A.M., Candau, R.B., and Bernardi, H. (2014). FoxO transcription factors: their roles in the maintenance of skeletal muscle homeostasis. Cell. Mol. Life Sci. *71*, 1657–1671. https://doi.org/10.1007/s00018-013-1513-z.
34. Xu, J., Wang, K., Zhang, Z., Xue, D., Li, W., and Pan, Z. (2021). The role of forkhead box family in bone metabolism and diseases. Front. Pharmacol. *12*, 772237. https://doi.org/10.3389/fphar.2021.772237.
35. Chal, J., and Pourquié, O. (2017). Making muscle: skeletal myogenesis in vivo and in vitro. Development *144*, 2104–2122. https://doi.org/10.1242/dev.151035.
36. Borman, W.H., and Yorde, D.E. (1994). Analysis of chick somite myogenesis by in situ confocal microscopy of desmin expression. J. Histochem. Cytochem. *42*, 265–272. https://doi.org/10.1177/42.2.8288867.
37. Berti, F., Nogueira, J.M., Wöhrle, S., Sobreira, D.R., Hawrot, K., and Dietrich, S. (2015). Time course and side-by-side analysis of mesodermal, pre-myogenic, myogenic and differentiated cell markers in the chicken model for skeletal muscle formation. J. Anat. *227*, 361–382. https://doi.org/10.1111/joa.12353.

38. Mok, G.F., Mohammed, R.H., and Sweetman, D. (2015). Expression of myogenic regulatory factors in chicken embryos during somite and limb development. J. Anat. *227*, 352–360. https://doi.org/10.1111/joa.12340.

39. Maschner, A., Krück, S., Draga, M., Pröls, F., and Scaal, M. (2016). Developmental dynamics of occipital and cervical somites. J. Anat. *229*, 601–609. https://doi.org/10.1111/joa.12516.

40. Green, J.D., Tollemar, V., Dougherty, M., Yan, Z., Yin, L., Ye, J., Collier, Z., Mohammed, M.K., Haydon, R.C., Luu, H.H., et al. (2015). Multifaceted signaling regulators of chondrogenesis: implications in cartilage regeneration and tissue engineering. Genes Dis. *2*, 307–327. https://doi.org/10.1016/j.gendis.2015.09.003.

41. Molkentin, J.D., Black, B.L., Martin, J.F., and Olson, E.N. (1995). Cooperative activation of muscle gene expression by MEF2 and myogenic bHLH proteins. Cell *83*, 1125–1136. https://doi.org/10.1016/0092-8674(95)90139-6.

42. Nimmagadda, S., Geetha Loganathan, P., Huang, R., Scaal, M., Schmidt, C., and Christ, B. (2005). BMP4 and noggin control embryonic blood vessel formation by antagonistic regulation of VEGFR-2 (Quek1) expression. Dev. Biol. *280*, 100–110. https://doi.org/10.1016/j.ydbio.2005.01.005.

43. Kartha, V.K., Duarte, F.M., Hu, Y., Ma, S., Chew, J.G., Lareau, C.A., Earl, A., Burkett, Z.D., Kohlway, A.S., Lebofsky, R., et al. (2022). Functional inference of gene regulation using single-cell multi-omics. Cell Genom. *2*. https://doi.org/10.1016/j.xgen.2022.100166.

44. Soshnikova, N., and Duboule, D. (2009). Epigenetic temporal control of mouse Hox genes in vivo. Science *324*, 1320–1323. https://doi.org/10.1126/science.1171468.

45. Chang, Y.C., Manent, J., Schroeder, J., Wong, S.F.L., Hauswirth, G.M., Shylo, N.A., Moore, E.L., Achilleos, A., Garside, V., Polo, J.M., et al. (2022). Nr6a1 controls Hox expression dynamics and is a master regulator of vertebrate trunk development. Nat. Commun. *13*, 7766. https://doi.org/10.1038/s41467-022-35303-4.

46. Gomez, C., Ozbudak, E.M., Wunderlich, J., Baumann, D., Lewis, J., and Pourquié, O. (2008). Control of segment number in vertebrate embryos. Nature *454*, 335–339. https://doi.org/10.1038/nature07020.

47. Mok, G.F., Folkes, L., Weldon, S.A., Maniou, E., Martinez-Heredia, V., Godden, A.M., Williams, R.M., Sauka-Spengler, T., Wheeler, G.N., Moxon, S., et al. (2021). Characterising open chromatin in chick embryos identifies cis-regulatory elements important for paraxial mesoderm formation and axis extension. Nat. Commun. *12*, 1157. https://doi.org/10.1038/s41467-021-21426-7.

48. Chal, J., Oginuma, M., Al Tanoury, Z., Gobert, B., Sumara, O., Hick, A., Bousson, F., Zidouni, Y., Mursch, C., Moncuquet, P., et al. (2015). Differentiation of pluripotent stem cells to muscle fiber to model Duchenne muscular dystrophy. Nat. Biotechnol. *33*, 962–969. https://doi.org/10.1038/nbt.3297.

49. Xi, H., Fujiwara, W., Gonzalez, K., Jan, M., Liebscher, S., Van Handel, B., Schenke-Layland, K., and Pyle, A.D. (2017). In vivo human somitogenesis guides somite development from hPSCs. Cell Rep. *18*, 1573–1585. https://doi.org/10.1016/j.celrep.2017.01.040.

50. Sivakamasundari, V., Kraus, P., Sun, W., Hu, X., Lim, S.L., Prabhakar, S., and Lufkin, T. (2017). A developmental transcriptomic analysis of Pax1 and Pax9 in embryonic intervertebral disc development. Biol. Open *6*, 187–199. https://doi.org/10.1242/bio.023218.

51. Tam, P.P. (1981). The control of somitogenesis in mouse embryos. J. Embryol. Exp. Morphol. *65* (*Suppl*), 103–128.

52. Qiu, C., Martin, B.K., Welsh, I.C., Daza, R.M., Le, T.-M., Huang, Xingfan, Nichols, E.K., Taylor, M.L., Fulton, O., Oâ Day, D.R., et al. (2023). A single-cell transcriptional timelapse of mouse embryonic development, from gastrula to pup. Preprint at bioRxiv. https://doi.org/10.1101/2023.04.05.535726.

53. Buenrostro, J.D., Wu, B., Chang, H.Y., and Greenleaf, W.J. (2015). ATAC-seq: a method for assaying chromatin accessibility genome-wide. Curr. Protoc. Mol. Biol. *109*, 21.29.1–21.29.9. https://doi.org/10.1002/0471142727.mb2129s109.

54. Dobin, A., Davis, C.A., Schlesinger, F., Drenkow, J., Zaleski, C., Jha, S., Batut, P., Chaisson, M., and Gingeras, T.R. (2013). STAR: ultrafast universal RNA-seq aligner. Bioinformatics *29*, 15–21. https://doi.org/10.1093/bioinformatics/bts635.

55. Robinson, M.D., McCarthy, D.J., and Smyth, G.K. (2010). edgeR: a Bioconductor package for differential expression analysis of digital gene expression data. Bioinformatics *26*, 139–140. https://doi.org/10.1093/bioinformatics/btp616.

56. McCarthy, D.J., Chen, Y., and Smyth, G.K. (2012). Differential expression analysis of multifactor RNA-Seq experiments with respect to biological variation. Nucleic Acids Res. *40*, 4288–4297. https://doi.org/10.1093/nar/gks042.

57. Love, M.I., Huber, W., and Anders, S. (2014). Moderated estimation of fold change and dispersion for RNA-seq data with DESeq2. Genome Biol. *15*, 550. https://doi.org/10.1186/s13059-014-0550-8.

58. Ritchie, M.E., Phipson, B., Wu, D., Hu, Y., Law, C.W., Shi, W., and Smyth, G.K. (2015). limma powers differential expression analyses for RNA-sequencing and microarray studies. Nucleic Acids Res. *43*, e47. https://doi.org/10.1093/nar/gkv007.

59. Lun, A.T., McCarthy, D.J., and Marioni, J.C. (2016). A step-by-step workflow for low-level analysis of single-cell RNA-seq data with Bioconductor. F1000Res *5*, 2122. https://doi.org/10.12688/f1000research.9501.2.

60. Li, H., and Durbin, R. (2009). Fast and accurate short read alignment with Burrows-Wheeler transform. Bioinformatics *25*, 1754–1760. https://doi.org/10.1093/bioinformatics/btp324.

61. Li, H., Handsaker, B., Wysoker, A., Fennell, T., Ruan, J., Homer, N., Marth, G., Abecasis, G., and Durbin, R.; 1000 Genome Project Data Processing Subgroup (2009). The Sequence Alignment/Map format and SAMtools. Bioinformatics *25*, 2078–2079. https://doi.org/10.1093/bioinformatics/btp352.

62. Lun, A.T., and Smyth, G.K. (2016). csaw: a Bioconductor package for differential binding analysis of ChIP-seq data using sliding windows. Nucleic Acids Res. *44*, e45. https://doi.org/10.1093/nar/gkv1191.

63. Durinck, S., Moreau, Y., Kasprzyk, A., Davis, S., De Moor, B., Brazma, A., and Huber, W. (2005). BioMart and Bioconductor: a powerful link between biological databases and microarray data analysis. Bioinformatics *21*, 3439–3440. https://doi.org/10.1093/bioinformatics/bti525.

64. Quinlan, A.R., and Hall, I.M. (2010). BEDTools: a flexible suite of utilities for comparing genomic features. Bioinformatics *26*, 841–842. https://doi.org/10.1093/bioinformatics/btq033.

65. Zhang, Y., Liu, T., Meyer, C.A., Eeckhoute, J., Johnson, D.S., Bernstein, B.E., Nusbaum, C., Myers, R.M., Brown, M., Li, W., et al. (2008). Model-based analysis of ChIP-Seq (MACS). Genome Biol. *9*, R137. https://doi.org/10.1186/gb-2008-9-9-r137.

66. Alexa, A., and Rahnenfuhrer, J. (2022). topGO: enrichment analysis for gene ontology R Package Version 2.50.0. https://doi.org/10.18129/B9.bioc.topGO.

67. Marini, F., and Binder, H. (2019). pcaExplorer: an R/Bioconductor package for interacting with RNA-seq principal components. BMC Bioinformatics *20*, 331. https://doi.org/10.1186/s12859-019-2879-1.

68. Marini, F., Ludt, A., Linke, J., and Strauch, K. (2021). GeneTonic: an R/Bioconductor package for streamlining the interpretation of RNA-seq data. BMC Bioinformatics *22*, 610. https://doi.org/10.1186/s12859-021-04461-5.

69. Gu, Z., and Hübschmann, D. (2023). rGREAT: an R/Bioconductor package for functional enrichment on genomic regions. Bioinformatics *39*. https://doi.org/10.1093/bioinformatics/btac745.

70. McLeay, R.C., and Bailey, T.L. (2010). Motif Enrichment Analysis: a unified framework and an evaluation on ChIP data. BMC Bioinformatics *11*, 165. https://doi.org/10.1186/1471-2105-11-165.

71. Schep, A.N., Wu, B., Buenrostro, J.D., and Greenleaf, W.J. (2017). chromVAR: inferring transcription-factor-associated accessibility from single-cell epigenomic data. Nat. Methods *14*, 975–978. https://doi.org/10.1038/nmeth.4401.

CellPress
OPEN ACCESS

72. Corces, M.R., Trevino, A.E., Hamilton, E.G., Greenside, P.G., Sinnott-Armstrong, N.A., Vesuna, S., Satpathy, A.T., Rubin, A.J., Montine, K.S., Wu, B., et al. (2017). An improved ATAC-seq protocol reduces background and enables interrogation of frozen tissues. Nat. Methods *14*, 959–962. https://doi.org/10.1038/nmeth.4396.

73. Pituello, F., Medevielle, F., Foulquier, F., and Duprat, A.M. (1999). Activation of Pax6 depends on somitogenesis in the chick embryo cervical spinal cord. Development *126*, 587–596. https://doi.org/10.1242/dev.126.3.587.

74. Robinson, M.D., and Oshlack, A. (2010). A scaling normalization method for differential expression analysis of RNA-seq data. Genome Biol. *11*, R25. https://doi.org/10.1186/gb-2010-11-3-r25.

75. Amemiya, H.M., Kundaje, A., and Boyle, A.P. (2019). The ENCODE blacklist: identification of problematic regions of the genome. Sci. Rep. *9*, 9354. https://doi.org/10.1038/s41598-019-45839-z.

76. McLean, C.Y., Bristor, D., Hiller, M., Clarke, S.L., Schaar, B.T., Lowe, C.B., Wenger, A.M., and Bejerano, G. (2010). GREAT improves functional interpretation of cis-regulatory regions. Nat. Biotechnol. *28*, 495–501. https://doi.org/10.1038/nbt.1630.

77. Bailey, T.L., Boden, M., Buske, F.A., Frith, M., Grant, C.E., Clementi, L., Ren, J., Li, W.W., and Noble, W.S. (2009). MEME SUITE: tools for motif discovery and searching. Nucleic Acids Res. *37*, W202–W208. https://doi.org/10.1093/nar/gkp335.

STAR★METHODS

KEY RESOURCES TABLE

REAGENT or RESOURCE	SOURCE	IDENTIFIER
**Chemicals, peptides, and recombinant proteins**		
NEB Next High Fidelity 2X PCR Master Mix	NEB	Catalog # M0541L
EvaGreen Dye 20X in water	Biotum	Catalog #31000
Agencourt AMPure XP Beads	Beckman Coulter, Inc.	Cat. no. A63880
SUPERase-In RNase Inhibitor	Invitrogen	Cat no. AM2696
Dispase II (neutral protease, grade II)	Roche	Cat no. 04942078001
DMEM, high glucose, NEAA, no glutamine	Gibco	Cat. no. 10938025
**Critical commercial assays**		
Nextera XT DNA Library Preparation Kit	Illumina	Cat. no. FC-131-1096
Nextera DNA Sample Preparation kit	Illumina	Cat. no. FC-121-1030
MinElute PCR Purification Kit	Qiagen	Cat. no. / ID: 28004
Zymo Clean & Concentrator kit	Zymo Research	Cat. no. D4014
SMART-seq v4 Ultra Low Input RNA Kit for Sequencing	TaKaRa	Cat. no. 634891
**Deposited data**		
Raw and processed RNA-seq data	This paper	ArrayExpress: E-MTAB-12511
Raw and processed ATAC-seq data	This paper	ArrayExpress: E-MTAB-12539
**Experimental models: Organisms/strains**		
Mouse: C57BL/6J	Charles River	The Jackson Laboratory
**Oligonucleotides**		
Tn5ME-A TCGTCGGCAGCGTCAGATGTGTATAAGAGACAG	Illumina	Cat. no. FC-121-1030
Tn5ME-B GTCTCGTGGGCTCGGAGATGTGTATAAGAGACAG	Illumina	Cat. no. FC-121-1031
Ad1_uni AATGATACGGCGACCACCGAGATCTACACTCGT CGGCAGCGTCAGATGTG	Eurofins	Buenrostro et al.[53]
Ad2.1 CAAGCAGAAGACGGCATACGAGATTCGCCTTAGTCTCG TGGGCTCGGAGATGT	Eurofins	Buenrostro et al.[53]
Ad2.2 CAAGCAGAAGACGGCATACGAGATCTAGTACGGTCTC GTGGGCTCGGAGATGT	Eurofins	Buenrostro et al.[53]
Ad2.3 CAAGCAGAAGACGGCATACGAGATTTCTGCCTGTCTCG TGGGCTCGGAGATGT	Eurofins	Buenrostro et al.[53]
Ad2.4 CAAGCAGAAGACGGCATACGAGATGCTCAGGAGTCTC GTGGGCTCGGAGATGT	Eurofins	Buenrostro et al.[53]
Ad2.5 CAAGCAGAAGACGGCATACGAGATAGGAGTCCGTCTC GTGGGCTCGGAGATGT	Eurofins	Buenrostro et al.[53]
Ad2.7 CAAGCAGAAGACGGCATACGAGATGTAGAGAGGTCTC GTGGGCTCGGAGATGT	Eurofins	Buenrostro et al.[53]
Ad2.9 CAAGCAGAAGACGGCATACGAGATAGCGTAGCGTCTC GTGGGCTCGGAGATGT	Eurofins	Buenrostro et al.[53]
Ad2.10 CAAGCAGAAGACGGCATACGAGATCAGCCTCGGTCT CGTGGGCTCGGAGATGT	Eurofins	Buenrostro et al.[53]
Ad2.11 CAAGCAGAAGACGGCATACGAGATTGCCTCTTGTCTC GTGGGCTCGGAGATGT	Eurofins	Buenrostro et al.[53]
Ad2.12 CAAGCAGAAGACGGCATACGAGATTCCTCTACGTCTC GTGGGCTCGGAGATGT	Eurofins	Buenrostro et al.[53]
**Software and algorithms**		
STAR 2.6.0c	Dobin et al.[54]	https://github.com/alexdobin/STAR
edgeR	Robinson et al.,[55] McCarthy et al.[56]	https://bioconductor.org/packages/release/bioc/html/edgeR.html

(*Continued on next page*)

Continued

REAGENT or RESOURCE	SOURCE	IDENTIFIER
DESeq2	Love et al.[57]	https://bioconductor.org/packages/release/bioc/html/DESeq2.html
Limma	Ritchie et al.[58]	https://www.bioconductor.org/packages/release/bioc/html/limma.html
Scran	Lun et al.[59]	https://www.bioconductor.org/packages/release/bioc/html/scran.html
bwa mem	Li and Durbin[60]	https://github.com/lh3/bwa
Samtools	Li et al.[61]	http://www.htslib.org/
Picard tools	Broad Institute	http://broadinstitute.github.io/picard/
Csaw	Lun and Smyth[62]	https://bioconductor.org/packages/release/bioc/html/csaw.html
biomaRt	Durinck et al.[63]	https://bioconductor.org/packages/release/bioc/html/biomaRt.html
Bedtools	Quinlan and Hall[64]	https://github.com/arq5x/bedtools2
MACS2	Zhang et al.[65]	https://pypi.org/project/MACS2/
topGO	Alexa and Rahnenfuhrer[66]	https://bioconductor.org/packages/release/bioc/html/topGO.html
pcaExplorer	Marini and Binder[67]	https://bioconductor.org/packages/release/bioc/html/pcaExplorer.html
GeneTonic	Marini et al.[68]	https://bioconductor.org/packages/release/bioc/html/GeneTonic.html
rGREAT	Gu and Hübschmann[69]	https://bioconductor.org/packages/release/bioc/html/rGREAT.html
AME	McLeay and Bailey[70]	https://meme-suite.org/meme/doc/ame.html
chromVAR	Schep et al.[71]	http://www.bioconductor.org/packages/release/bioc/html/chromVAR.html
FigR	Kartha et al.[43]	https://buenrostrolab.github.io/FigR/

RESOURCE AVAILABILITY

Lead contact

Further information and requests for resources and reagents should be directed to and will be fulfilled by the lead contact, Ximena Ibarra-Soria (ximena.x.ibarra-soria@gsk.com)

Materials availability

This study did not generate new unique reagents.

Data and code availability

- Raw and processed RNA-seq and ATAC-seq data have been deposited in the ArrayExpress repository. Accession numbers are listed in the key resources table.
- All original code is available at GitHub (https://github.com/xibarrasoria/somitogenesis2022).
- Any additional information required to reanalyse the data reported in this paper is available from the lead contact upon request.

EXPERIMENTAL MODEL AND STUDY PARTICIPANT DETAILS

All experiments followed the Animals (Scientific Procedures) Act 1986 (United Kingdom) and with the approval of the Cancer Research UK Cambridge Institute Animal Welfare and Ethical Review Body (form number: NRWF-DO-01- v3). Animal experiments conformed to the Animal Research: Reporting of *In Vivo* Experiments (ARRIVE) guidelines developed by the National Centre for the Replacement, Refinement and Reduction of Animals in Research (NC3Rs). C57BL/6J strain mice were obtained from Charles River Laboratories and maintained under standard husbandry practices: mice were group housed in Techniplast GM500 Mouse IVC Green Line cages in a room with 12 h light/12 h dark cycle and *ad libitum* access to water and food (LabDiet 5058). Cages contained aspen bedding and cage enrichments (nesting material, aspen chew stick, and cardboard tunnel).

METHOD DETAILS

Mouse embryo collection and dissection

Mouse embryos from the appropriate somite stages (8 to 35 somites) were dissected in RNAse-free conditions in cold PBS, on silicone plates. Utmost care was taken to accurately count the number of somite pairs of each embryo; however, for the 35-somite stage embryos this task becomes very difficult and it is possible that there is a one or two somite error range in the number estimated. Photos of all the embryos profiled are provided in File S1. To dissect out the somites, embryos were treated with dispase II (1mg/mL in DMEM) for 30-45 seconds at 37°C. The three most posterior pairs of somites were then collected using tungsten needles, dissecting out every somite separately. We labelled each somite pair as somite I, II or III from the most posterior to the most anterior, respectively. Thus, somite I corresponds to the most recently segmented somite, while somites II and III were segmented ~2 and ~4 h before[3]; we refer to this as the somite's age. Each individual somite was placed in 10μL of lysis buffer (Takara) containing RNase inhibitor. One somite from each pair was flash frozen in liquid nitrogen and stored at -80°C for later processing for RNA-seq. The matching somites were directly processed to generate ATAC-seq libraries.

Experimental design

We collected at least four different embryos from each developmental stage (Table S1). Dissections were performed on ten different days with every stage represented on at least two separate collection dates. However, samples from the three earliest stages were collected on five days, without overlap with the samples from the remaining three stages, resulting in a partially confounded design.

RNA-seq experiments and library preparation

Reverse transcription was performed directly on frozen lysed somites and cDNA was amplified with 8 cycles of PCR, using the SMART-seq v4 Ultra Low Input RNA Kit for Sequencing (TaKaRa, 634891). RNA-seq libraries were generated from 100pg of amplified cDNA using the NEXTERA XT DNA Library Preparation kit (Illumina, FC-131-1096), according to the manufacturer's instructions, except only a quarter of the recommended reagents' amount was used. The resulting libraries were quantified using a Qubit instrument and their size distributions were assessed with a TapeStation machine. Pooled libraries were sequenced on an Illumina HiSeq 4000 according to manufacturer's instructions to produce paired-end 150bp reads.

ATAC-seq experiments and library preparation

ATAC-seq experiments were performed following the protocol from Corces and colleagues,[72] originally modified from Buenrostro et al.[53] Briefly, individual somites were lysed and transposed with 1μL of transposome (Nextera DNA Sample Preparation kit FC-121-1030) at 37°C for 30 minutes. Samples were then purified with the Zymo Clean & Concentrator kit and eluted in 21μL of elution buffer. Transposed DNA was quantified by qPCR using 5 μl of PCR products. The number of additional cycles was determined by plotting linear Rn versus cycle and corresponded to one third of the maximum fluorescence intensity. Transposed DNA was then amplified with 13 cycles of PCR. The final products were double size-selected with AMPure beads (0.55X - 1.5X) to obtain fragments between 100bp and 700bp. Libraries were quantified and the sizes were assessed with a TapeStation machine. Pooled libraries were sequenced on an Illumina HiSeq 4000 according to manufacturer's instructions to produce paired-end 150bp reads. Samples were sequenced to a median depth of 70.5 million fragments.

QUANTIFICATION AND STATISTICAL ANALYSIS

RNA-seq data processing and quality control

RNA-seq paired-end fragments were aligned to the mouse reference genome (GRCm38) with STAR 2.6.0c[54] with options–outFilterMismatchNmax 6 –outFilterMatchNminOverLread 0.5 –outFilterScoreMinOverLread 0.5 –outSAMtype BAM SortedByCoordinate –outFilterType BySJout –outFilterMultimapNmax 20 –alignSJoverhangMin 8 –alignSJDBoverhangMin 1 –alignIntronMin 20 –alignIntronMax 1000000 –alignMatesGapMax 1000000 –outSAMstrandField intronMotif. On average, 84% of the sequencing fragments mapped uniquely. We also set the option –quantMode GeneCounts to quantify the number of fragments overlapping annotated transcripts, using Ensembl's genome annotation version 96 (http://apr2019.archive.ensembl.org/index.html).

Samples were sequenced to a median depth of 17.4 million paired-end fragments. One sample had a library size of only 88 thousand fragments and was discarded. All other samples showed a uniform number of fragments mapped uniquely (median 85.6%, standard deviation (SD) 5.6%) and most of these were within annotated exons (median 84.9%, SD 2.8%). On average, we detected around 22 thousand expressed genes per sample (Table S2).

To validate the staging of samples we exploited the Hox code that serves as a molecular indicative of developmental stage. As shown in Figures 1C and 1D, our embryo stages defined by the observed number of somites agreed with the expected expression levels of Hox genes. However, samples from one stage 27 embryo were more similar to the stage 35 somites, showing expression of several late Hox genes from paralogous groups 12 and 13 that are only observed in the stage 35 samples. These data suggest this particular embryo was likely of a more advanced stage than 27 somites and was removed from downstream analyses (Table S1).

Finally, to assess the purity of our somite samples, we assessed the expression levels of marker genes for notochord (*Shh* and *Noto*), neural crest (*Foxd3* and *Sox10*) and neural tube (*Olig2* and *Pax6*). We observed very low counts for all these genes, except *Pax6*, suggesting minimal carryover of these tissues into our samples. *Pax6* has been shown to be expressed in neuroepithelial cells neighbouring newly formed somites,[73] indicating a small amount of contamination from these cells is present in the data. *Pax6* levels were similar to those of markers of presomitic mesoderm *Tbx6* and *Msgn1*. However, all these genes were detected several fold lower compared to somitic mesoderm markers such as *Pax3*, *Foxc2*, *Meox1* or *Tcf15*, suggesting the amount of contaminating tissue is minimal.

RNA-seq data normalization

Downstream analyses were restricted to genes with at least 10 counts in three or more samples, as implemented in the filterByExpr function from the edgeR package[55,56]; this represents 20,062 genes. To normalise for differences in sequencing depth we used the calcNormFactors function that implements the weighted trimmed mean of M-values method,[74] and generated counts-per-million normalised expression estimates.

A PCA of the thousand most variable genes (determined from variance-stabilised data, computed with the vst function form the DESeq2[57] package) showed good separation of samples from different developmental stages (Figure S1B). However, we also observed subgrouping by the date of collection, indicating substantial batch effects (Figure S1B). Since the experimental design is partially confounded with the date of collection of the samples, we were unable to include this as a covariate in downstream analyses. Instead, to control for technical variation unrelated to the biological variables of interest, we used the function lmFit from the limma package[58] to fit a linear model of the combination of developmental stage and somite age for each sample. We then performed PCA on the residuals from the fit (function residuals) to capture systematic variation unrelated to the biological design of interest. To determine how many principal components (PCs) captured significant variation we used the parallelPCA function from the scran package[59] on the normalised counts; this function estimates, via permutation analysis, the number of PCs that explain more variation than expected by chance, which in our case was 14. Thus, the 14 first PCs were used as covariates in downstream analyses to control for unwanted variation (Figure S1C). We note that this procedure captures both technical and biological variation not modelled in our design of interest (i.e. the sex of the embryos).

RNA-seq differential expression analysis

To identify genes significantly differentially expressed across conditions we used edgeR,[55,56] with a design matrix of the interaction of each sample's age and developmental stage, plus the 14 PCs representing technical variation as covariates. Dispersion was estimated with the estimateDisp function (setting robust = TRUE) and fitting the model with glmQLFit. Specific contrasts were then tested with the glmQLFTest function.

To identify the regions that change as somites differentiate we compared samples from somites I, II and III. To identify conserved differences across development we defined contrasts for all three pairwise comparisons, using the average of same-age samples from all six stages:

$$somite_{i.vs.j} = \sum_{k \in \{8,18,21,25,27,35\}} stage_k.somite_i \Big/ 6 - \sum_{k \in \{8,18,21,25,27,35\}} stage_k.somite_j \Big/ 6$$

where *i.vs.j* corresponds to *I.vs.II*, *I.vs.III* and *II.vs.III*. To recover possible stage-specific changes, we also defined contrasts on a per-stage basis:

$$somite_{i.vs.j}.stage_k = somite_i.stage_k - somite_j.stage_k$$

where *k* is one of the six stages and *i.vs.j* the same as above. All three pairwise comparisons from each stage were tested at once. Thus, in these cases the p-value indicates whether the gene is differentially expressed between at least a pair of somite ages.

We used a similar approach to test for differences in expression across development. Conserved differences between all somites irrespective of their maturity level were assessed by averaging somites I, II and III and testing each pairwise comparison between the six stages:

$$stage_{k.vs.l} = \sum_{i \in \{I,II,III\}} somite_i.stage_k \Big/ 3 - \sum_{i \in \{I,II,III\}} somite_i.stage_l \Big/ 3$$

where *k.vs.l* corresponds to all pairwise comparisons between the six stages. All contrasts were tested at once to avoid performing too many tests and, again, p-values indicate whether the gene is significantly different between at least a pair of stages. To check for any changes specific to a given somite age we repeated the analysis separately for somites I, II and III:

$$stage_{k.vs.l}.somite_i = stage_k.somite_i - stage_l.somite_i$$

where *i* is any of the three somite ages and *k.vs.l* the same as above. Genes were considered significantly differentially expressed if their adjusted p-value was lower than 0.05 (FDR < 5%) and their absolute fold-change was greater than 1.5. Results from all differential expression analyses are available at https://github.com/xibarrasoria/somitogenesis2022.

ATAC-seq data alignment

Raw sequencing reads were aligned to the mouse reference genome (GRCm38) using bwa mem 0.7.12-r1039[60] with default parameters. On average, 93% of the total fragments were successfully aligned. The resulting SAM files were processed with samtools 1.5.[61] One sample was sequenced to a disproportionately high depth compared to the rest (1.5 billion fragments compared to a median of 70.5 million). The mapped data for this sample was downsampled to 15% of the total reads (samtools -s 0.15), and the resulting BAM file was used in the downstream processing steps.

Duplicated fragments were marked and removed using MarkDuplicates 1.103 from Picard tools (http://broadinstitute.github.io/picard) with option REMOVE_DUPLICATES=TRUE. We further used samtools to remove any pairs that were not properly aligned (-f 0x02); supplementary alignments (-F 0x800); alignments with mapping quality lower than 30 (-q 30); and alignments outside the autosomes or chromosome X. The resulting BAM files represent the clean, good quality alignments used in all downstream analyses.

ATAC-seq quality control

To assess the quality of the libraries we used three different criteria: 1) the insert size distribution of the sequenced fragments; 2) the level of signal enrichment at the transcription start site (TSS) of expressed genes; and 3) the signal-to-noise ratio, assessed by the ability to call peaks (Figure S2; Table S3).

To compute the insert size distribution of each library we used the getPESizes function from the csaw package.[62] Each library's distribution was visually inspected and scored based on the number of nucleosomal peaks. Thus, a score of 0 implies that only short fragments were recovered, a score of 1 indicates presence of mononucleosomes, 2 corresponds to samples with both monomers and dimers, and so on. The maximum score assigned was 4, including samples with fragment sizes corresponding to nucleosome tetramers or larger (Figure S2A).

To estimate the enrichment of fragments at transcription start sites we applied the method recommended by the ENCODE standards for ATAC-seq data (https://www.encodeproject.org/data-standards/terms/#enrichment). Specifically, we restricted the analysis to genes with moderate to high expression as assessed from the RNA-seq data (mean normalised counts per million greater than 10, corresponding to 8,025 genes). We then used the biomaRt package[63] to extract the most 5′ TSS for each gene and created a BED file of 2kb intervals centred at each TSS. We computed the coverage of such intervals using bedtools coverage 2.26.0[64] a BEDPE file containing the Tn5 insertion sites inferred from the aligned fragments (by shifting the start/end coordinates by +5/-4 bp with an ad hoc perl script). To calculate the enrichment at the TSS we first computed the mean insertion counts at each base pair from all genes. We then used the mean of the first and last 100bp as an estimate of the background insertion rate. For each base pair, we computed the enrichment score as the fold-change against the background rate; this results in an enrichment score of ∼1 at the flanks of the 2kb interval which increases as it approaches the TSS (Figure S2B).

Finally, to assess the signal-to-noise ratio of each sample we used MACS2 2.1[65] to call peaks, with options callpeak -f BAMPE -g mm –keep-dup all –broad. Peaks overlapping blacklisted regions[75] (obtained from https://github.com/Boyle-Lab/Blacklist/blob/master/lists/mm10-blacklist.v2.bed.gz) were discarded. We calculated the fraction of reads in peaks (FRiP) as the total fragments overlapping called peaks over the total library size and used this, along with the total number of peaks, as proxies for the signal-to-noise ratio (Figure S2C).

Libraries with an insert size distribution showing a good nucleosomal pattern generally had good TSS enrichment scores and signal-to-noise ratios. For each sample we defined a quality control pass if they had an insert size distribution score of 2 or higher; a fraction of reads in peaks of 3% or larger; at least 15,000 peaks; and a TSS enrichment score of 5 or higher (Figure S2D). Samples satisfying at least three of these criteria were annotated as good quality and used in downstream analyses (50 of the 75 libraries). Importantly, insert size distribution scores were positively correlated with the experimentally measured DNA fragment sizes but showed no relation to sequencing depth, indicating that samples with poor quality control characteristics are not due to insufficient sequencing (Figure S2E).

ATAC-seq peak calling

To define a unified set of peaks for the whole dataset we combined the clean BAM files from the 50 samples that passed quality control and used them as input for MACS2 (same parameters as stated above). By calling peaks on the combined data from all samples, the peak calling process becomes agnostic to the different conditions in our experimental design, which is important for downstream differential accessibility analyses. After removing peaks overlapping blacklisted regions, a total of 131,743 peaks were called, with a median width of 777 bp (interquartile range 418-1394 bp). We re-computed the FRiP for each sample using this common peak set.

It is possible that by merging all samples together, some low-enrichment stage-specific peaks are lost. Thus, we repeated the peak calling procedure but on a stage-specific basis. When comparing the per-stage peak calls to the set obtained by using all 50 samples, around 96.6% of the peaks called in each stage were also called in the all-sample set (range 93.43-98.09%). The small proportion of peaks missed generally had small fold-changes and high q-values, and thus correspond to low significance calls. This provides confidence that we have not missed stage-specific peaks by merging data from all samples.

ATAC-seq data normalization

To normalise the ATAC-seq data we used the methods implemented in the csaw package.[62] We generated MA plots comparing all pairs of samples by counting the number of fragments in 10 kb windows tiling the genome. For high-abundance windows, which

correspond to open chromatin, we observed a deviation of the $\log_2$ fold-change from the expected value of 0 that was correlated with the abundance level of the genomic region (Figure S3A). Conventional normalization techniques used in the majority of ATAC-seq analyses compute a single size factor that captures systematic differences between samples; these approaches fail to account for the trend observed in our data (Figure S3B). Thus, we instead used a loess-based approach to compute size factors specific to each abundance level. For this, we counted the number of fragments mapped to 150 bp windows, sliding along the genome by 50 bp, using the function windowCounts (with filter set to 75 and excluding any reads overlapping blacklisted regions). We then filtered out any windows that did not overlap the common peak set or that had less than an average count of 4 fragments across samples. We finally used this set of windows to compute the size factors with the normOffsets function (with type=loess). This approach successfully removed the observed trend (Figure S3C). However, the first principal component estimated from the normalised counts of the 5000 most variable windows was strongly correlated with samples' FRiP (Pearson's r = -0.59), suggesting other technical effects were still dominant in the data (Figure S3D).

To remove unwanted variation from the dataset we used the same strategy that we applied to the RNA-seq samples. That is, we obtained the residuals from a linear model fit of the interaction of the stage and somite age of each sample and applied PCA to capture the major sources of variation. We retained the first 18 PCs, since these were deemed to explain significantly more variation than chance (as determined by the parallelPCA function), which significantly removed efficiency and batch effects (Figure S3E).

ATAC-seq differential accessibility analysis

To test for differences in accessibility across conditions we used the approach implemented in csaw.[62] We based the analysis on the window counts described above. Window counts along with the corresponding size factors were converted into a DGEList object compatible with edgeR[55] to perform differential analysis. The same approach as described for the RNA-seq data was used.

After each window was tested, we used the mergeWindows function to merge windows that were no more than 150 bp apart, restricting the maximum width to 1.5 kb. Regions larger than 1.5 kb were broken into smaller overlapping regions of roughly equal size (+/- 100bp). We then computed a combined p-value for each of these regions with the combineTests function, using Simes' method. Correction for multiple testing was performed at the region level and regions were considered significantly different if their adjusted p-value was lower than 0.05 and their absolute fold-change was greater than 1.5. Results from all differential expression analyses are available at https://github.com/xibarrasoria/somitogenesis2022.

Functional terms enrichment analysis

Gene ontology enrichment analysis was performed using the elim method from the TopGo package,[66] as implemented in the topGOtable function from the PCAexplorer 2.18.0 package.[67] Enrichment of GO terms among differentially expressed genes was computed using all genes expressed in somites as the background. Results were visualised with the GeneTonic R package.[68]

Enrichment analysis of GO terms and mouse KO phenotypes in differentially accessible chromatin regions was computed with GREAT,[76] using the implementation from the rGREAT 1.24.0 package.[69] All somite peaks were used as the background set.

Motif enrichment analysis

To determine transcription factor (TF) motifs enriched in the regions of open chromatin, we used Analysis of Motif Enrichment[70] from the MEME suite,[77] with the human and mouse HOCOMOCOv11_full motif databases. Enrichment in differentially accessible regions (either between somite ages, or between stages, split by the vertebral fate showing highest accessibility) was computed by comparing to a set of non-differentially accessible regions with a similar length distribution.

chromVAR

To estimate the accessibility dynamics of sites harbouring specific TF binding sites we used chromVAR 1.12.0,[71] on the normalised and corrected window counts described above. Following the authors' recommendations, we removed overlapping windows with the filterPeaks function, and then scanned them for matches to the motif collection provided with the package (mouse_pwms_v2). Accessibility deviation scores were then computed with the computeDeviations function. For all plots, we use the z-scores returned by this function.

FigR

To infer peak-gene putative regulatory links we used FigR 0.1.0,[43] restricted to the normalised and corrected counts of the 43 samples with both RNA and ATAC-seq profiles available. The function runGenePeakcorr was used to compute the correlation between each differentially expressed gene and all peaks within 100kb. This function uses chromVAR to determine a set of 100 background peaks matched for accessibility and GC content levels, to determine if the observed correlation of the gene-peak of interest is significantly higher than the correlation of the gene to these unrelated background peaks. chromVAR samples with replacement to define the background set. When there are not enough matching peaks, the number of *different* peaks in the background set can drop substantially; in extreme cases this can result in a single peak repeated 100 times. Using this distribution as a null is non-informative and thus the p-values computed for such gene-peak pairs are misleading. To avoid these cases, we modified the code to check for the number of different peaks included in the background and set the p-values to NA for any gene-peak pairs with background sets

containing fewer than 50 different peaks. Downstream analyses were performed using gene-peak pairs with a p-value < 0.05 and a correlation score greater than 0.3. To infer regulatory interactions, we used the getDORCScores and runFigRGRN functions, on all genes with more than 5 linked peaks. TF-gene pairs with an absolute regulation score greater than 1.25 are considered putative interactions (Figures 4C and 4D).

ADDITIONAL RESOURCES

Website to query the data and results from this publication: https://crukci.shinyapps.io/somitogenesis/

Developmental Cell, Volume *58*

Supplemental information

A transcriptional and regulatory map of mouse somite maturation

Ximena Ibarra-Soria, Elodie Thierion, Gi Fay Mok, Andrea E. Münsterberg, Duncan T. Odom, and John C. Marioni

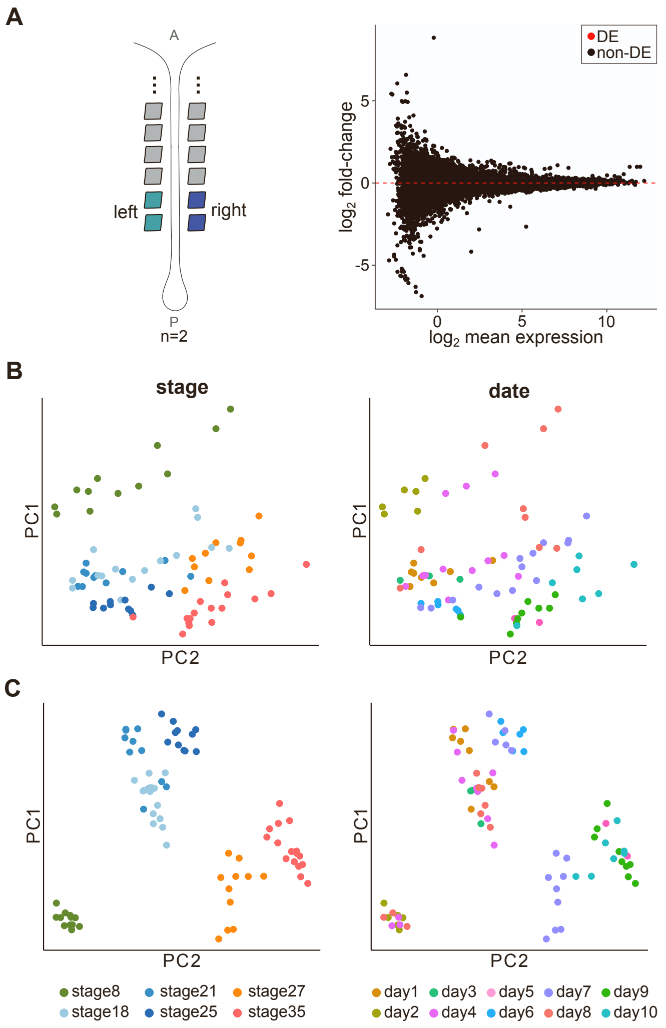

Figure S1 | The left and right somites are transcriptionally equivalent, related to STAR Methods. **A)** We compared the transcriptomes of matched left and right somites by RNA-seq using the two most posterior somite pairs, as shown in the schematic. A: anterior; P: posterior. The scatter plot shows the average gene expression level on the x-axis and the corresponding $\log_2$ fold-change between the left and right somites on the y-axis. No significantly differentially expressed (DE) genes were identified (FDR 5%), indicating that the transcriptomes of the two somites from the same pair are equivalent. **B)** PCA of the normalised counts of the thousand most variable genes across samples. There is clear separation by developmental stage (left). However, PC1 also separates samples based on their collection day (right). **C)** PCA after regressing out covariates capturing variation unrelated to the experimental design. Samples separate better by their developmental stage (left) and grouping by collection day is no longer evident (right).

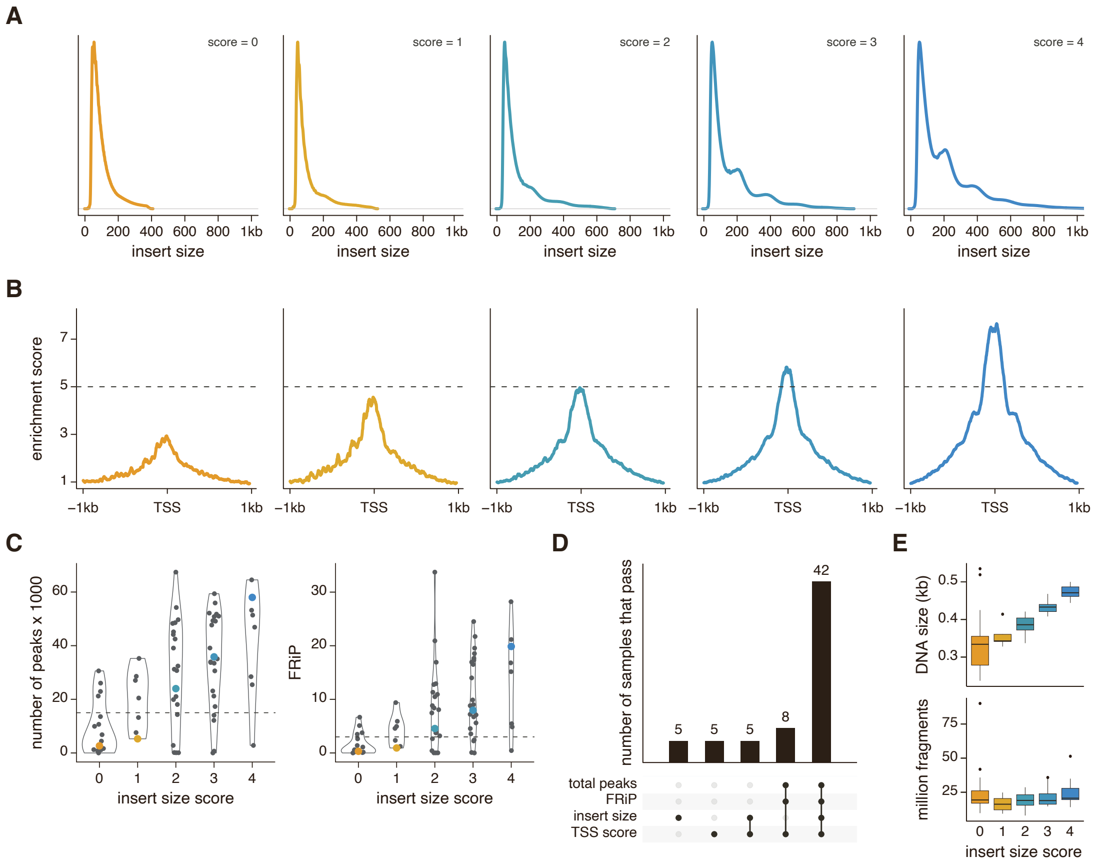

Figure S2 | Quality control of ATAC-seq libraries, related to STAR Methods.

Several metrics were used to determine if the ATAC-seq libraries were of good quality. **A)** Representative density plots of the fragment sizes of the sequencing data for each score group. Scores were determined based on the number of nucleosomal peaks. Colours indicate different score groups, with yellow (blue) shades assigned to poor (good) quality samples. **B)** Representative plots of the cumulative signal for 2 kb intervals centred at the transcription start site (TSS) of expressed genes, for the same samples shown in A. An enrichment score larger than 1 indicates an excess of insertions relative to background. Signal is smoothed by taking the rolling median of 25 bp intervals. **C)** Violin plots depicting the number of total peaks called from each library (left) and the fraction of reads in peaks (FRiP; right), stratified by the sample's insert size distribution score. The samples depicted in A-B are highlighted by coloured points. **D)** Number of samples that pass each of the QC criteria. The 50 samples that passed three or four criteria were deemed of good enough quality for downstream analyses. Samples that failed all four criteria are not shown. **E)** Boxplots of the experimentally determined DNA fragment size (top) and the library size of the sequenced samples (bottom), stratified by the insert size distribution score. No relationship is observed between library size and size distribution score, indicating that the poor-quality samples are not due to insufficient sequencing.

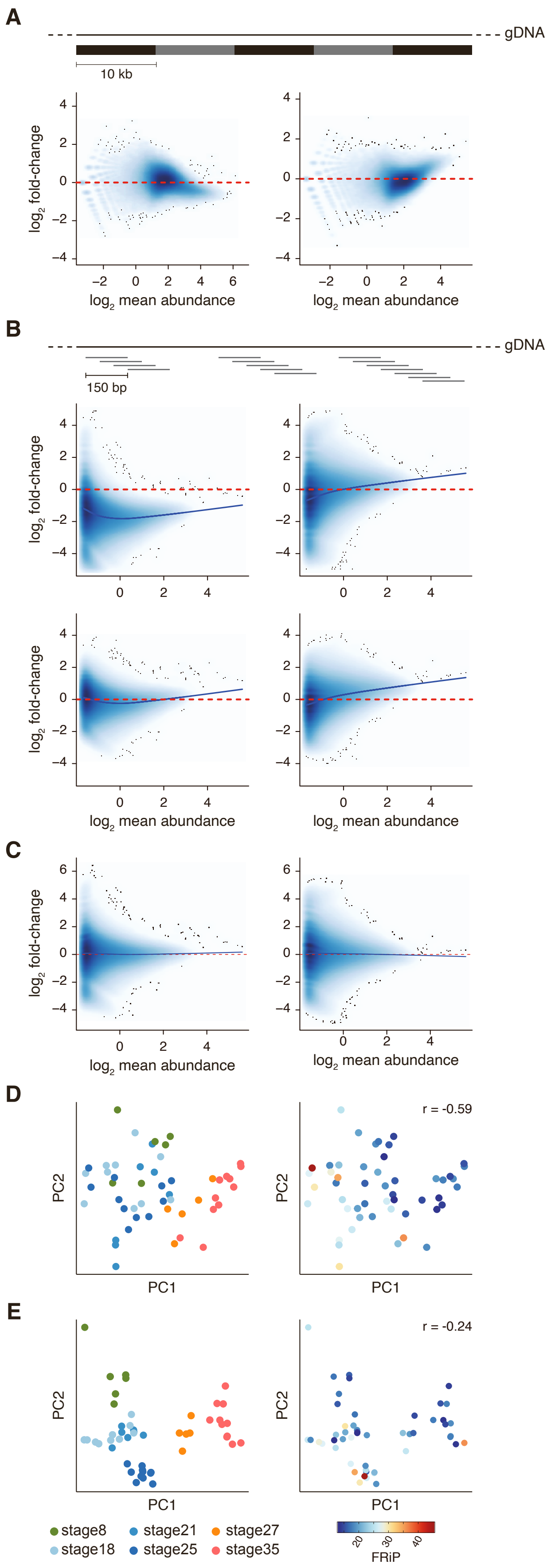

Figure S3 | Normalisation of ATAC-seq data, related to STAR Methods.

A) MA plots of two representative samples. On the x-axis is the log_2 average number of sequencing fragments in 10kb bins covering the mouse genome. The y-axis corresponds to the log_2 fold-change against a reference sample (same for both panels). Comparable samples should show log fold-changes centred around 0. High abundance bins (which contain open regions) show significant deviation from 0. This deviation shows a trend dependent on mean abundance. **B)** To normalise the observed biases we focused on the regions of open chromatin. The MA plots now show on the x-axis the average counts in 150bp windows that slide 50bp, restricted to regions overlapping called peaks. At the top, the raw counts for the same samples in A. At the bottom, scaling normalisation is applied, which results in a shift towards a fold-change of 0. However, the observed trend dependent on mean abundance is still present in the data. **C)** MA plots for the same samples in B, but after applying loess-based normalisation, which computes a size factor for each abundance level. This successfully captured and removed the observed trend, with windows now centred around 0. **D)** PCA of the normalised counts of the 5000 most variable windows. On the left, samples are coloured by developmental stage while on the right they are coloured by their fraction of reads in peaks (FRiP). There is clear grouping of samples based on their FRiP. The Pearson correlation coefficient between FRiP and PC1 is noted. **E)** PCA after regressing out covariates capturing variation unrelated to the experimental design. Samples separate better by their developmental stage (left) and there is no longer a significant correlation with FRiP (right).

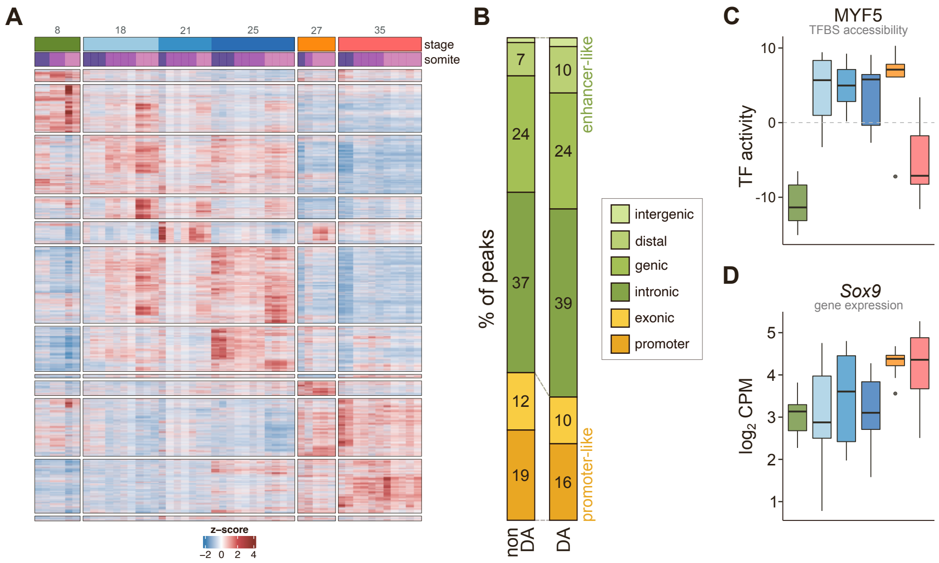

Figure S4 | Differentially accessible chromatin loci across development, related to Figure 3.

A) Similar to Figure 3B but showing the accessibility levels of all differential peaks across development. Samples (columns) are ordered based on their observed somite number, and their stage and somite level are indicated at the top. Peaks (rows) are split into clusters by hierarchical clustering. **B)** Same as Figure 2E but for differentially accessible regions across development. **C)** Chromatin activity scores from chromVAR for the genome-wide binding sites (TFBS) of MYF5 across development. **D)** Gene expression levels for *Sox9* across development.

						RNA-seq				ATAC-seq			
sampleName	embryo	somite	somiteNumber	somiteStage	collectionDate	RNA_ID	RNA_QC	size (bp)	concentration [nM]	ATAC_ID	ATAC_QC	size (bp)	concentration [nM]
e5_SI	e5	SI	8	8	06.04.18	do26267	1	474	2.42	do26190	1	486	5.78
e5_SII	e5	SII	7	8	06.04.18	do26268	1	442	3.56	do26191	0	425	9.5
e5_SIII	e5	SIII	6	8	06.04.18	do26269	1	439	3	do26192	0	469	10.76
e6_SI	e6	SI	8	8	06.04.18	do26270	1	408	2.76	do26193	0	425	5.86
e6_SII	e6	SII	7	8	06.04.18	do26271	1	403	1.88	do26194	0	439	7.1
e6_SIII	e6	SIII	6	8	06.04.18	do26272	1	390	1.5	NA	NA	NA	NA
e16_SI	e16	SI	8	8	26.04.18	do26285	1	445	2.4	do26205	0	341	6.26
e16_SII	e16	SII	7	8	26.04.18	do26286	1	451	3.1	do26206	1	342	7.88
e16_SIII	e16	SIII	6	8	26.04.18	do26287	1	440	2.9	do26207	1	420	6.08
e26_SI	e26	SI	8	8	01.06.18	do26314	1	470	8.2	do26233	1	437	7.74
e26_SII	e26	SII	7	8	01.06.18	do26315	1	527	12.88	do26234	1	372	5.04
e26_SIII	e26	SIII	6	8	01.06.18	do26316	1	518	8.36	do26235	1	444	8.12
e9_SI	e9	SI	18	18	12.04.18	do26273	1	484	2.36	do26195	1	423	6.84
e9_SII	e9	SII	17	18	12.04.18	do26274	1	416	3.04	do26196	0	414	9.7
e9_SIII	e9	SIII	16	18	12.04.18	do26275	1	438	4.98	do26197	1	437	7.46
e14_SI	e14	SI	18	18	26.04.18	do26279	1	500	6.16	NA	NA	NA	NA
e14_SII	e14	SII	17	18	26.04.18	do26280	1	474	5.06	do26201	1	473	7.72
e14_SIII	e14	SIII	16	18	26.04.18	do26281	1	467	3.98	do26202	0	425	7.56
e15_SI	e15	SI	18	18	26.04.18	do26282	1	474	3.18	do26203	1	393	6.8
e15_SII	e15	SII	17	18	26.04.18	do26283	1	497	2.9	do26204	1	447	6.64
e15_SIII	e15	SIII	16	18	26.04.18	do26284	1	449	3.8	NA	NA	NA	NA
e27_SI	e27	SI	18	18	01.06.18	do26317	1	489	13.08	do26236	1	380	7.66
e27_SII	e27	SII	17	18	01.06.18	do26318	1	446	6.7	do26237	1	364	9.04
e27_SIII	e27	SIII	16	18	01.06.18	do26319	1	378	1.91	do26238	1	348	14.8
e28_SI	e28	SI	18	18	01.06.18	do26320	1	424	14.2	do26239	0	303	17.8
e28_SII	e28	SII	17	18	01.06.18	do26321	1	435	8.02	do26240	1	334	10.1
e28_SIII	e28	SIII	16	18	01.06.18	do26322	1	484	2.52	do26241	1	390	5.97
e1_SI	e1	SI	21	21	05.04.18	do26259	1	479	5.66	do26181	1	328	18
e1_SII	e1	SII	20	21	05.04.18	do26260	1	522	4.32	do26182	1	368	18.6
e1_SIII	e1	SIII	19	21	05.04.18	do26261	1	477	3.76	do26183	0	344	18.1
e2_SI	e2	SI	21	21	05.04.18	do26262	1	469	4.46	do26184	0	396	8.87
e2_SII	e2	SII	20	21	05.04.18	NA	NA	NA	NA	do26185	1	421	6.42
e2_SIII	e2	SIII	19	21	05.04.18	do26263	1	412	3.68	do26186	0	337	12.1
e3_SI	e3	SI	21	21	05.04.18	do26264	1	466	8.98	do26187	0	376	14.9
e3_SII	e3	SII	20	21	05.04.18	do26265	1	488	7.88	do26188	1	409	8.44
e3_SIII	e3	SIII	19	21	05.04.18	do26266	1	514	9.92	do26189	0	414	11.2
e13_SI	e13	SI	21	21	26.04.18	do26276	1	469	9.62	do26198	1	441	7.38
e13_SII	e13	SII	20	21	26.04.18	do26277	1	500	2.4	do26199	1	489	6.22
e13_SIII	e13	SIII	19	21	26.04.18	do26278	1	496	3.76	do26200	1	447	6.76
e19_SI	e19	SI	25	25	20.05.18	do26294	1	504	9.12	do26214	1	421	11.4
e19_SII	e19	SII	24	25	20.05.18	do26295	1	501	6.7	do26215	1	408	6.19
e19_SIII	e19	SIII	23	25	20.05.18	do26296	1	518	9.94	do26216	0	415	8.24
e20_SI	e20	SI	25	25	20.05.18	do26297	1	446	3.9	do26217	1	468	5.3
e20_SII	e20	SII	24	25	20.05.18	do26298	1	415	7.82	do26218	1	419	9.44
e20_SIII	e20	SIII	23	25	20.05.18	do26299	1	422	3	do26219	1	441	6.75
e21_SI	e21	SI	25	25	24.05.18	do26300	1	465	11.9	do26220	1	404	8.26
e21_SII	e21	SII	24	25	24.05.18	NA	NA	NA	NA	do26221	1	461	7.58
e21_SIII	e21	SIII	23	25	24.05.18	do26301	1	586	6.98	do26222	1	438	7.32
e22_SI	e22	SI	25	25	24.05.18	do26302	1	508	7.8	do26223	1	433	5.7
e22_SII	e22	SII	24	25	24.05.18	do26303	1	557	8.86	do26224	1	421	7.34
e22_SIII	e22	SIII	23	25	24.05.18	do26304	1	520	9.14	do26225	1	407	8.58
e23_SI	e23	SI	27	27	24.05.18	do26305	1	590	26.8	do26226	1	237	5.88
e23_SII	e23	SII	26	27	24.05.18	do26306	1	510	8.82	do26227	0	280	32.2
e23_SIII	e23	SIII	25	27	24.05.18	do26307	1	385	1.6	do26228	0	372	8.05
e24_SI	e24	SI	27	27	24.05.18	do26308	1	473	12.98	do26229	1	343	14.8
e24_SII	e24	SII	26	27	24.05.18	do26309	1	437	8.88	do26230	1	405	6.22
e24_SIII	e24	SIII	25	27	24.05.18	do26310	1	391	13.3	do26231	1	361	8.52
e25_SI	e25	SI	27	27	24.05.18	do26311	1	486	14.8	do26232	1	380	9.48
e25_SII	e25	SII	26	27	24.05.18	do26312	1	546	14.12	NA	NA	NA	NA
e25_SIII	e25	SIII	25	27	24.05.18	do26313	1	576	16	NA	NA	NA	NA
e34_SI	e34	SI	27	27	14.06.18	do26337	1	450	16.66	do26256	0	356	15.7
e34_SII	e34	SII	26	27	14.06.18	do26338	1	476	17.52	do26257	0	274	14.3
e34_SIII	e34	SIII	25	27	14.06.18	NA	NA	NA	NA	do26258	0	324	30
e17_SI	e17	SI	35	35	27.04.18	do26288	1	507	15.8	do26208	1	466	7.94
e17_SII	e17	SII	34	35	27.04.18	do26289	0	528	12.2	do26209	1	449	7.52
e17_SIII	e17	SIII	33	35	27.04.18	do26290	1	543	7.82	do26210	1	382	9.8
e29_SI	e29	SI	35	35	11.06.18	do26323	1	468	13.76	do26242	0	535	10.86
e29_SII	e29	SII	34	35	11.06.18	do26324	1	470	12.02	do26243	1	372	15
e29_SIII	e29	SIII	33	35	11.06.18	do26325	1	528	9.04	do26244	0	360	19.2
e30_SI	e30	SI	35	35	11.06.18	do26326	1	489	14.42	do26245	1	386	10.3
e30_SII	e30	SII	34	35	11.06.18	do26327	1	497	12.5	do26246	1	393	12.5
e30_SIII	e30	SIII	33	35	11.06.18	do26328	1	492	4.94	NA	NA	NA	NA
e31_SI	e31	SI	35	35	11.06.18	do26329	1	506	7.46	do26247	1	262	39
e31_SII	e31	SII	34	35	11.06.18	do26330	1	469	10.14	do26248	0	269	20.2
e31_SIII	e31	SIII	33	35	11.06.18	do26331	1	496	7.1	do26249	0	334	37.1
e32_SI	e32	SI	35	35	14.06.18	do26332	1	468	12.3	do26250	1	419	6.73
e32_SII	e32	SII	34	35	14.06.18	NA	NA	NA	NA	do26251	1	436	6.36
e32_SIII	e32	SIII	33	35	14.06.18	do26333	1	428	2.52	do26252	1	500	6.92
e33_SI	e33	SI	35	35	14.06.18	do26334	1	453	21.6	do26253	1	424	10.76
e33_SII	e33	SII	34	35	14.06.18	do26335	1	448	7.42	do26254	0	355	5.82
e33_SIII	e33	SIII	33	35	14.06.18	do26336	1	476	11.84	do26255	0	519	7.78

Table S1 | Metadata of the samples collected and the RNA- and ATAC-seq libraries produced, related to STAR Methods.
The QC columns indicate whether the sample passed quality control (1) or not (0; highlighted in red); NA indicates that the sample did not yield a successful library for sequencing.

RNA-seq

sampleName	embryo	somite	somiteNumber	somiteStage	collectionDate	RNA_ID	RNA_QC	size (bp)	concentration [nM]	totalFragments	uniquelyMapped		multimapped		unmapped		uniqueInExons		numberGenes	QCpass
e5_SI	e5	SI	8	8	06.04.18	do26267	1	474	2.42	11,104,305	9,173,394	82.61%	732,698	6.60%	1,198,213	10.79%	7811293	85.15%	19,997	1
e5_SII	e5	SII	7	8	06.04.18	do26268	1	442	3.56	13,116,915	11,025,523	84.06%	835,470	6.37%	1,255,922	9.57%	9471526	85.91%	20,042	1
e5_SIII	e5	SIII	6	8	06.04.18	do26269	1	439	3	15,002,486	12,870,669	85.79%	972,766	6.48%	1,159,051	7.73%	11039612	85.77%	19,860	1
e6_SI	e6	SI	8	8	06.04.18	do26270	1	408	2.76	28,218,690	20,787,050	73.66%	2,052,009	7.27%	5,379,631	19.06%	16323073	78.53%	22,343	1
e6_SII	e6	SII	7	8	06.04.18	do26271	1	403	1.88	28,976,459	21,475,857	74.11%	2,018,767	6.97%	5,481,835	18.92%	16985675	79.09%	22,074	1
e6_SIII	e6	SIII	6	8	06.04.18	do26272	1	390	1.5	26,472,304	17,277,583	65.27%	2,092,029	7.90%	7,102,692	26.83%	13215689	76.49%	22,272	1
e16_SI	e16	SI	8	8	26.04.18	do26285	1	445	2.4	11,679,604	9,127,484	78.15%	783,202	6.71%	1,768,918	15.15%	7778120	85.22%	19,549	1
e16_SII	e16	SII	7	8	26.04.18	do26286	1	451	3.1	30,925,538	24,211,119	78.29%	2,204,497	7.13%	4,509,922	14.58%	20816820	85.98%	21,462	1
e16_SIII	e16	SIII	6	8	26.04.18	do26287	1	440	2.9	24,721,994	19,018,399	76.93%	1,775,099	7.18%	3,928,496	15.89%	16093472	84.62%	22,029	1
e26_SI	e26	SI	8	8	01.06.18	do26314	1	470	8.2	17,096,838	14,592,409	85.35%	1,074,804	6.29%	1,429,625	8.36%	12666068	86.80%	22,087	1
e26_SII	e26	SII	7	8	01.06.18	do26315	1	527	12.88	19,270,385	17,269,913	89.62%	1,071,621	5.56%	928,851	4.82%	14700672	85.12%	24,105	1
e26_SIII	e26	SIII	6	8	01.06.18	do26316	1	518	8.36	14,645,123	13,137,082	89.70%	797,987	5.45%	710,054	4.85%	11294665	85.98%	21,659	1
e9_SI	e9	SI	18	18	12.04.18	do26273	1	484	2.36	15,593,104	12,579,677	80.67%	1,007,566	6.46%	2,005,861	12.86%	10713991	85.17%	20,588	1
e9_SII	e9	SII	17	18	12.04.18	do26274	1	416	3.04	31,478,723	24,015,046	76.29%	2,163,684	6.87%	5,299,993	16.84%	20124042	83.80%	22,634	1
e9_SIII	e9	SIII	16	18	12.04.18	do26275	1	438	4.98	27,148,512	21,202,082	78.10%	1,943,573	7.16%	4,002,857	14.74%	18208045	85.88%	21,933	1
e14_SI	e14	SI	18	18	26.04.18	do26279	1	500	6.16	17,394,443	15,118,264	86.91%	1,065,859	6.13%	1,210,320	6.96%	12954009	85.68%	21,779	1
e14_SII	e14	SII	17	18	26.04.18	do26280	1	474	5.06	19,982,005	17,456,879	87.36%	1,152,538	5.77%	1,372,588	6.87%	14631050	83.81%	22,200	1
e14_SIII	e14	SIII	16	18	26.04.18	do26281	1	467	3.98	10,890,982	9,421,084	86.50%	658,028	6.04%	811,870	7.45%	7986486	84.77%	20,732	1
e15_SI	e15	SI	18	18	26.04.18	do26282	1	474	3.18	17,078,623	13,481,094	78.94%	1,172,388	6.86%	2,425,141	14.20%	10788866	80.03%	22,038	1
e15_SII	e15	SII	17	18	26.04.18	do26283	1	497	2.9	10,364,313	8,441,580	81.45%	678,653	6.55%	1,244,080	12.00%	7027280	83.25%	20,432	1
e15_SIII	e15	SIII	16	18	26.04.18	do26284	1	449	3.8	13,957,817	10,892,022	78.04%	969,397	6.95%	2,096,398	15.02%	9125957	83.79%	21,273	1
e27_SI	e27	SI	18	18	01.06.18	do26317	1	489	13.08	14,965,632	13,169,244	88.00%	873,075	5.83%	923,313	6.17%	11188830	84.96%	22,448	1
e27_SII	e27	SII	17	18	01.06.18	do26318	1	446	6.7	18,204,147	15,468,276	84.97%	1,138,716	6.26%	1,597,155	8.77%	13239896	85.59%	20,979	1
e27_SIII	e27	SIII	16	18	01.06.18	do26319	1	378	1.91	23,044,782	15,687,604	68.07%	1,957,936	8.50%	5,399,242	23.43%	13016231	82.97%	21,107	1
e28_SI	e28	SI	18	18	01.06.18	do26320	1	424	14.2	21,814,598	18,827,750	86.31%	1,407,966	6.45%	1,578,882	7.24%	16485741	87.56%	21,396	1
e28_SII	e28	SII	17	18	01.06.18	do26321	1	435	8.02	16,757,748	14,073,742	83.98%	1,088,761	6.50%	1,595,245	9.52%	12076425	85.81%	20,790	1
e28_SIII	e28	SIII	16	18	01.06.18	do26322	1	484	2.52	15,095,217	12,315,332	81.58%	1,033,774	6.85%	1,746,111	11.57%	10363915	84.15%	20,544	1
e1_SI	e1	SI	21	21	05.04.18	do26259	1	479	5.66	19,115,686	15,918,562	83.27%	1,309,923	6.85%	1,887,201	9.87%	13636604	85.66%	21,988	1
e1_SII	e1	SII	20	21	05.04.18	do26260	1	522	4.32	16,223,856	13,632,341	84.03%	1,052,151	6.49%	1,539,364	9.49%	11380276	83.48%	22,523	1
e1_SIII	e1	SIII	19	21	05.04.18	do26261	1	477	3.76	16,078,906	13,362,203	83.10%	1,060,086	6.59%	1,656,617	10.30%	11447256	85.67%	21,115	1
e2_SI	e2	SI	21	21	05.04.18	do26262	1	469	4.46	17,745,006	14,931,863	84.15%	1,149,455	6.48%	1,663,688	9.38%	12962117	86.81%	20,462	1
e2_SIII	e2	SIII	19	21	05.04.18	do26263	1	412	3.68	26,208,112	21,044,414	80.30%	1,732,159	6.61%	3,431,539	13.09%	17537606	83.34%	22,003	1
e3_SI	e3	SI	21	21	05.04.18	do26264	1	466	8.98	12,070,510	10,319,762	85.50%	773,006	6.40%	977,742	8.10%	8651097	83.83%	21,326	1
e3_SII	e3	SII	20	21	05.04.18	do26265	1	488	7.88	8,914,760	7,662,977	85.96%	558,011	6.26%	693,772	7.78%	6375753	83.20%	20,696	1
e3_SIII	e3	SIII	19	21	05.04.18	do26266	1	514	9.92	9,473,279	8,231,138	86.89%	598,800	6.32%	643,341	6.79%	6683762	81.20%	24,827	1
e13_SI	e13	SI	21	21	26.04.18	do26276	1	469	9.62	19,031,213	16,444,192	86.41%	1,187,389	6.24%	1,399,632	7.35%	13907261	84.57%	21,801	1
e13_SII	e13	SII	20	21	26.04.18	do26277	1	500	2.4	5,274,715	4,539,096	86.05%	342,472	6.49%	393,147	7.45%	3854885	84.93%	18,565	1
e13_SIII	e13	SIII	19	21	26.04.18	do26278	1	496	3.76	12,628,888	10,178,030	80.59%	797,088	6.31%	1,653,770	13.10%	7895052	77.57%	22,795	1
e19_SI	e19	SI	25	25	20.05.18	do26294	1	504	9.12	14,886,262	12,901,995	86.67%	887,518	5.96%	1,096,749	7.37%	10948970	84.86%	21,142	1
e19_SII	e19	SII	24	25	20.05.18	do26295	1	501	6.7	16,366,161	14,062,380	85.92%	1,037,077	6.34%	1,266,704	7.74%	12199572	86.75%	21,038	1
e19_SIII	e19	SIII	23	25	20.05.18	do26296	1	518	9.94	17,630,796	15,148,231	85.92%	1,065,552	6.04%	1,417,013	8.04%	12944414	85.45%	21,879	1
e20_SI	e20	SI	25	25	20.05.18	do26297	1	446	3.9	21,319,850	17,305,153	81.17%	1,368,369	6.42%	2,646,328	12.41%	14187805	81.99%	22,506	1
e20_SII	e20	SII	24	25	20.05.18	do26298	1	415	7.82	15,418,029	12,469,799	80.88%	921,318	5.98%	2,026,912	13.15%	10109585	81.07%	22,276	1
e20_SIII	e20	SIII	23	25	20.05.18	do26299	1	422	3	16,551,117	13,177,452	79.62%	974,416	5.89%	2,399,249	14.50%	10478383	79.52%	22,499	1
e21_SI	e21	SI	25	25	24.05.18	do26300	1	465	11.9	14,032,161	12,045,302	85.84%	833,824	5.94%	1,153,035	8.22%	10029660	83.27%	21,805	1
e21_SIII	e21	SIII	23	25	24.05.18	do26301	1	586	6.98	19,797,880	17,365,881	87.72%	1,175,918	5.94%	1,256,081	6.34%	14389506	82.86%	23,832	1
e22_SI	e22	SI	25	25	24.05.18	do26302	1	508	7.8	14,736,983	12,770,927	86.66%	1,042,722	7.08%	923,334	6.27%	11009395	86.21%	21,737	1
e22_SII	e22	SII	24	25	24.05.18	do26303	1	557	8.86	16,622,239	14,928,806	89.81%	944,611	5.68%	748,822	4.50%	12848950	86.07%	21,395	1
e22_SIII	e22	SIII	23	25	24.05.18	do26304	1	520	9.14	16,896,833	15,083,645	89.27%	983,086	5.82%	830,102	4.91%	13056410	86.56%	21,854	1

sampleName	embryo	somite	somiteNumber	somiteStage	collectionDate	RNA_ID	RNA_QC	size (bp)	concentration [nM]	totalFragments	uniquelyMapped		multimapped		unmapped		uniqueInExons		numberGenes	QCpass
e23_SI	e23	SI	27	27	24.05.18	do26305	1	590	26.8	7,988,810	6,947,827	86.97%	495,655	6.20%	545,328	6.83%	5977893	86.04%	20,149	1
e23_SII	e23	SII	26	27	24.05.18	do26306	1	510	8.82	21,886,661	19,168,094	87.58%	1,327,708	6.07%	1,390,859	6.35%	16418749	85.66%	23,407	1
e23_SIII	e23	SIII	25	27	24.05.18	do26307	1	385	1.6	22,118,311	13,060,634	59.05%	1,803,166	8.15%	7,254,511	32.80%	9098507	69.66%	23,499	1
e24_SI	e24	SI	27	27	24.05.18	do26308	1	473	12.98	47,578,277	41,692,027	87.63%	3,002,440	6.31%	2,883,810	6.06%	35909124	86.13%	25,226	1
e24_SII	e24	SII	26	27	24.05.18	do26309	1	437	8.88	38,420,055	31,791,135	82.75%	3,309,846	8.61%	3,319,074	8.64%	27773459	87.36%	24,161	1
e24_SIII	e24	SIII	25	27	24.05.18	do26310	1	391	13.3	31,117,702	26,144,820	84.02%	2,177,639	7.00%	2,795,243	8.98%	22629196	86.55%	23,983	1
e25_SI	e25	SI	27	27	24.05.18	do26311	1	486	14.8	24,509,980	20,539,533	83.80%	2,208,717	9.01%	1,761,730	7.19%	18139676	88.32%	23,452	1
e25_SII	e25	SII	26	27	24.05.18	do26312	1	546	14.12	21,765,155	19,324,858	88.79%	1,377,513	6.33%	1,062,784	4.88%	16730504	86.58%	23,325	1
e25_SIII	e25	SIII	25	27	24.05.18	do26313	1	576	16	21,884,352	19,485,576	89.04%	1,364,596	6.24%	1,034,180	4.73%	17027011	87.38%	23,057	1
e34_SI	e34	SI	27	27	14.06.18	do26337	1	450	16.66	33,546,238	29,193,121	87.02%	2,262,418	6.74%	2,090,699	6.23%	24729002	84.71%	25,963	1
e34_SII	e34	SII	26	27	14.06.18	do26338	1	476	17.52	24,831,126	22,068,851	88.88%	1,520,308	6.12%	1,241,967	5.00%	18430704	83.51%	25,214	1
e17_SI	e17	SI	35	35	27.04.18	do26288	1	507	15.8	15,111,788	13,227,335	87.53%	923,753	6.11%	960,700	6.36%	11068316	83.68%	22,645	1
e17_SII	e17	SII	34	35	27.04.18	do26289	1	528	12.2	88,342	71,120	80.51%	6,268	7.10%	10,954	12.40%	59881	84.20%	8,159	0
e17_SIII	e17	SIII	33	35	27.04.18	do26290	1	543	7.82	24,112,273	20,926,547	86.79%	1,474,496	6.12%	1,711,230	7.10%	17689437	84.53%	23,960	1
e29_SI	e29	SI	35	35	11.06.18	do26323	1	468	13.76	27,241,840	23,423,979	85.99%	2,066,584	7.59%	1,751,277	6.43%	20182512	86.16%	23,686	1
e29_SII	e29	SII	34	35	11.06.18	do26324	1	470	12.02	19,935,563	17,051,356	85.53%	1,453,390	7.29%	1,430,817	7.18%	14463539	84.82%	24,695	1
e29_SIII	e29	SIII	33	35	11.06.18	do26325	1	528	9.04	12,410,163	10,739,506	86.54%	786,704	6.34%	883,953	7.12%	8895134	82.83%	22,049	1
e30_SI	e30	SI	35	35	11.06.18	do26326	1	489	14.42	15,437,320	13,568,906	87.90%	928,398	6.01%	940,016	6.09%	11259137	82.98%	23,051	1
e30_SII	e30	SII	34	35	11.06.18	do26327	1	497	12.5	14,387,094	12,502,964	86.90%	923,627	6.42%	960,503	6.68%	10795415	86.34%	21,445	1
e30_SIII	e30	SIII	33	35	11.06.18	do26328	1	492	4.94	11,655,620	9,978,735	85.61%	782,173	6.71%	894,712	7.68%	8253505	82.71%	21,641	1
e31_SI	e31	SI	35	35	11.06.18	do26329	1	506	7.46	23,218,716	19,641,242	84.59%	1,452,083	6.25%	2,125,391	9.15%	16435852	83.68%	23,301	1
e31_SII	e31	SII	34	35	11.06.18	do26330	1	469	10.14	15,194,366	13,221,314	87.01%	960,989	6.32%	1,012,063	6.66%	11083470	83.83%	22,647	1
e31_SIII	e31	SIII	33	35	11.06.18	do26331	1	496	7.1	22,757,132	19,427,603	85.37%	1,415,503	6.22%	1,914,026	8.41%	16270683	83.75%	24,511	1
e32_SI	e32	SI	35	35	14.06.18	do26332	1	468	12.3	25,719,125	22,599,520	87.87%	1,595,615	6.20%	1,523,990	5.93%	18921965	83.73%	24,665	1
e32_SIII	e32	SIII	33	35	14.06.18	do26333	1	428	2.52	17,945,810	13,162,156	73.34%	1,649,726	9.19%	3,133,928	17.46%	10670675	81.07%	20,929	1
e33_SI	e33	SI	35	35	14.06.18	do26334	1	453	21.6	23,850,064	20,744,926	86.98%	1,591,931	6.67%	1,513,207	6.34%	17885958	86.22%	22,422	1
e33_SII	e33	SII	34	35	14.06.18	do26335	1	448	7.42	23,934,429	20,276,034	84.71%	1,833,093	7.66%	1,825,302	7.63%	17363611	85.64%	25,072	1
e33_SIII	e33	SIII	33	35	14.06.18	do26336	1	476	11.84	39,323,747	33,632,900	85.53%	3,004,980	7.64%	2,685,867	6.83%	29112812	86.56%	25,033	1

Table S2 | Quality control statistics from the RNA-seq libraries, related to STAR Methods.
Only one sample failed QC (in red) due to insufficient sequencing depth (totalFragments). The 'uniqueInExons' column indicates the number of fragments uniquely mapped to the genome that also can be assigned unambiguously to annotated exons.

ATAC-seq																					
sampleName	embryo	somite	somiteNumber	somiteStage	collectionDate	ATAC_ID	ATAC_QC	size (bp)	concentration [nM]	totalFragments	mapped		unique		goodQuality		insertSizeDist	numberPeaks	readsInPeaks	TSSenrichment	QCpass
e5_SI	e5	SI	8	8	06.04.18	do26190	1	486	5.78	41,520,261	37,764,252	90.95%	21,474,949	51.72%	20,125,635	93.72%	4	53,210	3,382,072	6.31	1
e5_SII	e5	SII	7	8	06.04.18	do26191	0	425	9.5	30,371,932	27,596,028	90.86%	16,843,741	55.46%	15,485,088	91.93%	3	7	481	2.30	0
e5_SIII	e5	SIII	6	8	06.04.18	do26192	0	469	10.76	32,466,242	29,472,826	90.78%	19,147,566	58.98%	18,121,596	94.64%	4	2,811	80,214	2.96	0
e6_SI	e6	SI	8	8	06.04.18	do26193	0	425	5.86	49,200,969	44,628,904	90.71%	26,846,785	54.57%	24,700,063	92.00%	3	14,058	554,250	3.75	0
e6_SII	e6	SII	7	8	06.04.18	do26194	0	439	7.1	43,376,906	39,417,510	90.87%	23,855,029	54.99%	21,925,536	91.91%	3	683	18,535	2.56	0
e16_SI	e16	SI	8	8	26.04.18	do26205	0	341	6.26	19,523,615	17,703,369	90.68%	10,864,615	55.65%	9,290,851	85.51%	1	5,273	86,320	4.50	0
e16_SII	e16	SII	7	8	26.04.18	do26206	1	342	7.88	20,962,982	19,057,936	90.91%	12,212,591	58.26%	10,659,160	87.28%	1	20,477	488,795	5.54	1
e16_SIII	e16	SIII	6	8	26.04.18	do26207	1	420	6.08	25,555,851	23,211,869	90.83%	13,987,736	54.73%	12,357,920	88.35%	2	39,098	1,036,066	5.28	1
e26_SI	e26	SI	8	8	01.06.18	do26233	1	437	7.74	69,169,028	62,813,968	90.81%	38,236,045	55.28%	35,810,413	93.66%	3	35,081	2,579,196	4.46	1
e26_SII	e26	SII	7	8	01.06.18	do26234	1	372	5.04	43,572,763	39,586,640	90.85%	25,586,947	58.72%	23,131,659	90.40%	2	23,985	1,054,320	4.31	1
e26_SIII	e26	SIII	6	8	01.06.18	do26235	1	444	8.12	67,280,508	61,033,758	90.72%	36,787,364	54.68%	34,969,382	95.06%	4	28,442	1,908,884	4.01	1
e9_SI	e9	SI	18	18	12.04.18	do26195	1	423	6.84	32,624,338	29,564,356	90.62%	16,503,078	50.59%	14,656,807	88.81%	3	51,754	2,539,535	6.73	1
e9_SII	e9	SII	17	18	12.04.18	do26196	0	414	9.7	44,416,466	40,355,298	90.86%	23,754,141	53.48%	21,837,354	91.93%	2	13,212	511,689	3.75	0
e9_SIII	e9	SIII	16	18	12.04.18	do26197	1	437	7.46	48,591,338	44,136,194	90.83%	26,668,661	54.88%	24,342,954	91.28%	3	33,463	1,727,050	4.67	1
e14_SII	e14	SII	17	18	26.04.18	do26201	1	473	7.72	57,427,746	52,127,849	90.77%	26,828,129	46.72%	25,503,529	95.06%	4	57,994	5,065,873	6.82	1
e14_SIII	e14	SIII	16	18	26.04.18	do26202	0	425	7.56	43,138,589	39,100,379	90.64%	23,329,698	54.08%	21,628,928	92.71%	0	4,411	129,143	2.83	0
e15_SI	e15	SI	18	18	26.04.18	do26203	1	393	6.8	21,142,333	19,168,513	90.66%	11,326,869	53.57%	9,826,753	86.76%	2	32,405	866,781	6.03	1
e15_SII	e15	SII	17	18	26.04.18	do26204	1	447	6.64	48,507,019	44,129,083	90.97%	25,268,838	52.09%	23,615,417	93.46%	3	49,130	3,306,248	6.09	1
e27_SI	e27	SI	18	18	01.06.18	do26236	1	380	7.66	61,879,228	56,150,469	90.74%	31,437,853	50.81%	28,226,636	89.79%	2	67,413	5,903,924	7.73	1
e27_SII	e27	SII	17	18	01.06.18	do26237	1	364	9.04	50,280,194	45,713,793	90.92%	28,119,885	55.93%	24,868,793	88.44%	2	48,291	2,717,378	6.50	1
e27_SIII	e27	SIII	16	18	01.06.18	do26238	1	348	14.8	43,095,342	39,106,388	90.74%	26,296,450	61.02%	23,083,729	87.78%	0	21,220	804,344	4.16	1
e28_SI	e28	SI	18	18	01.06.18	do26239	0	303	17.8	28,015,581	25,479,427	90.95%	18,180,641	64.89%	15,657,252	86.12%	0	1,152	19,708	2.66	0
e28_SII	e28	SII	17	18	01.06.18	do26240	1	334	10.1	35,113,967	31,943,185	90.97%	20,978,024	59.74%	18,137,094	86.46%	0	22,931	683,499	4.74	1
e28_SIII	e28	SIII	16	18	01.06.18	do26241	1	390	5.97	39,153,744	35,575,751	90.86%	21,865,433	55.85%	19,216,659	87.89%	1	49,674	2,175,964	6.15	1
e1_SI	e1	SI	21	21	05.04.18	do26181	1	328	18	26,285,398	23,805,193	90.56%	15,699,543	59.73%	13,500,945	86.00%	1	35,243	1,267,894	7.18	1
e1_SII	e1	SII	20	21	05.04.18	do26182	1	368	18.6	27,923,115	25,363,674	90.83%	16,868,650	60.41%	14,956,907	88.67%	2	31,206	1,206,547	6.58	1
e1_SIII	e1	SIII	19	21	05.04.18	do26183	0	344	18.1	22,545,472	20,472,674	90.81%	14,252,736	63.22%	12,675,623	88.93%	0	6	278	2.14	0
e2_SI	e2	SI	21	21	05.04.18	do26184	0	396	8.87	30,698,288	27,882,112	90.83%	18,694,786	60.90%	17,124,982	91.60%	2	150	3,419	2.70	0
e2_SII	e2	SII	20	21	05.04.18	do26185	1	421	6.42	42,503,669	38,660,336	90.96%	23,286,344	54.79%	21,466,478	92.18%	3	33,724	1,934,851	5.67	1
e2_SIII	e2	SIII	19	21	05.04.18	do26186	0	337	12.1	21,572,118	19,607,938	90.89%	14,192,023	65.79%	12,516,018	88.19%	1	81	1,816	2.87	0
e3_SI	e3	SI	21	21	05.04.18	do26187	0	376	14.9	27,883,412	25,342,010	90.89%	16,961,929	60.83%	15,491,632	91.33%	2	83	2,439	3.19	0
e3_SII	e3	SII	20	21	05.04.18	do26188	1	409	8.44	34,035,542	30,921,806	90.85%	20,205,853	59.37%	18,337,752	90.75%	2	21,201	834,258	4.99	1
e3_SIII	e3	SIII	19	21	05.04.18	do26189	0	414	11.2	33,237,354	30,189,528	90.83%	19,287,813	58.03%	17,771,925	92.14%	3	12,144	428,856	4.02	0
e13_SI	e13	SI	21	21	26.04.18	do26198	1	441	7.38	36,859,933	33,359,197	90.50%	16,685,449	45.27%	15,323,118	91.84%	3	52,140	2,841,072	7.44	1
e13_SII	e13	SII	20	21	26.04.18	do26199	1	489	6.22	46,126,231	41,858,744	90.75%	21,185,446	45.93%	20,090,201	94.83%	4	51,387	4,251,284	7.61	1
e13_SIII	e13	SIII	19	21	26.04.18	do26200	1	447	6.76	31,970,370	28,986,376	90.67%	15,388,171	48.13%	14,096,109	91.60%	3	46,888	2,137,431	6.38	1
e19_SI	e19	SI	25	25	20.05.18	do26214	1	421	11.4	31,525,091	28,547,683	90.56%	16,796,287	53.28%	15,641,546	93.13%	4	50,824	3,834,123	8.56	1
e19_SII	e19	SII	24	25	20.05.18	do26215	1	408	6.19	33,277,602	30,182,182	90.70%	17,514,786	52.63%	15,606,419	89.10%	2	49,316	2,644,494	7.47	1
e19_SIII	e19	SIII	23	25	20.05.18	do26216	0	415	8.24	46,082,074	41,704,050	90.50%	25,551,639	55.45%	23,172,217	90.69%	2	14,305	614,083	4.16	0
e20_SI	e20	SI	25	25	20.05.18	do26217	1	468	5.3	38,450,312	34,902,562	90.77%	18,187,382	47.30%	16,856,794	92.68%	3	47,689	2,977,092	7.50	1
e20_SII	e20	SII	24	25	20.05.18	do26218	1	419	9.44	36,502,685	33,219,443	91.01%	20,946,689	57.38%	19,397,511	92.60%	2	30,605	1,305,794	5.39	1
e20_SIII	e20	SIII	23	25	20.05.18	do26219	1	441	6.75	45,028,161	40,914,608	90.86%	26,120,484	58.01%	24,141,739	92.42%	3	39,115	2,386,067	6.08	1
e21_SI	e21	SI	25	25	24.05.18	do26220	1	404	8.26	18,193,619	16,499,754	90.69%	8,569,971	47.10%	7,956,487	92.84%	2	48,557	2,683,644	9.77	1
e21_SII	e21	SII	24	25	24.05.18	do26221	1	461	7.58	40,543,973	36,810,397	90.79%	20,627,302	50.88%	18,895,591	91.60%	3	51,010	3,118,988	7.06	1
e21_SIII	e21	SIII	23	25	24.05.18	do26222	1	438	7.32	35,421,485	32,182,060	90.85%	16,672,562	47.07%	15,308,685	91.82%	3	55,913	3,325,865	7.46	1
e22_SI	e22	SI	25	25	24.05.18	do26223	1	433	5.7	61,751,926	56,157,557	90.94%	30,769,038	49.83%	28,310,041	92.01%	3	59,371	5,535,868	7.42	1
e22_SII	e22	SII	24	25	24.05.18	do26224	1	421	7.34	41,092,266	37,305,894	90.79%	21,255,700	51.73%	19,013,735	89.45%	2	44,161	2,427,196	6.47	1
e22_SIII	e22	SIII	23	25	24.05.18	do26225	1	407	8.58	61,349,519	55,715,809	90.82%	31,691,112	51.66%	28,654,120	90.42%	2	45,151	3,003,688	6.20	1
e23_SI	e23	SI	27	27	24.05.18	do26226	1	237	5.88	16,621,773	15,083,965	90.75%	11,271,492	67.81%	9,699,800	86.06%	0	26,033	494,922	6.38	1
e23_SII	e23	SII	26	27	24.05.18	do26227	0	280	32.2	33,069,762	30,028,502	90.80%	21,955,382	66.39%	19,092,921	86.96%	0	2,652	58,996	2.79	0
e23_SIII	e23	SIII	25	27	24.05.18	do26228	0	372	8.05	32,049,089	29,116,276	90.85%	19,407,711	60.56%	17,276,310	89.02%	2	2,811	70,947	3.57	0
e24_SI	e24	SI	27	27	24.05.18	do26229	1	343	14.8	34,700,675	31,517,200	90.83%	21,766,776	62.73%	19,015,349	87.36%	1	28,575	1,122,828	5.36	1
e24_SII	e24	SII	26	27	24.05.18	do26230	1	405	6.22	36,776,104	33,402,398	90.83%	20,508,945	55.77%	18,505,572	90.23%	2	54,228	3,130,807	7.42	1
e24_SIII	e24	SIII	25	27	24.05.18	do26231	1	361	8.52	46,008,731	41,773,412	90.79%	28,414,076	61.76%	24,820,262	87.35%	1	27,050	1,227,985	4.74	1
e25_SI	e25	SI	27	27	24.05.18	do26232	1	380	9.48	51,623,765	46,831,482	90.72%	28,176,462	54.58%	25,520,753	90.57%	2	42,475	3,296,630	7.68	1
e34_SI	e34	SI	27	27	14.06.18	do26256	0	356	15.7	27,614,285	25,097,089	90.88%	18,076,382	65.46%	16,779,282	92.82%	0	1,239	25,151	2.23	0
e34_SII	e34	SII	26	27	14.06.18	do26257	0	274	14.3	58,378,625	52,989,711	90.77%	38,558,648	66.05%	35,014,726	90.81%	0	813	23,158	1.97	0
e34_SIII	e34	SIII	25	27	14.06.18	do26258	0	324	30	28,666,363	26,027,868	90.80%	18,397,554	64.18%	17,063,699	92.75%	0	1,653	35,847	2.64	0

sampleName	embryo	somite	somiteNumber	somiteStage	collectionDate	ATAC_ID	ATAC_QC	size (bp)	concentration [nM]	totalFragments	mapped		unique		goodQuality		insertSizeDist	numberPeaks	readsInPeaks	TSSenrichment	QCpass
e17_SI	e17	SI	35	35	27.04.18	do26208	1	466	7.94	45,492,062	41,369,622	90.94%	22,245,164	48.90%	21,162,155	95.13%	4	64,529	5,970,483	7.95	1
e17_SII	e17	SII	34	35	27.04.18	do26209	1	449	7.52	39,016,972	35,453,023	90.87%	19,977,394	51.20%	18,309,152	91.65%	3	33,926	1,614,976	5.28	1
e17_SIII	e17	SIII	33	35	27.04.18	do26210	1	382	9.8	32,048,955	29,156,005	90.97%	18,614,091	58.08%	16,632,055	89.35%	1	19,899	625,586	4.76	1
e29_SI	e29	SI	35	35	11.06.18	do26242	0	535	10.86	86,308,470	78,688,161	91.17%	42,112,412	48.79%	41,825,316	99.32%	0	9,935	458,883	1.72	0
e29_SII	e29	SII	34	35	11.06.18	do26243	1	372	15	37,337,982	33,945,412	90.91%	23,211,427	62.17%	20,946,981	90.24%	2	30,715	1,564,078	6.07	1
e29_SIII	e29	SIII	33	35	11.06.18	do26244	0	360	19.2	28,491,304	25,885,991	90.86%	17,628,708	61.87%	16,233,753	92.09%	0	7,598	198,393	3.78	0
e30_SI	e30	SI	35	35	11.06.18	do26245	1	386	10.3	35,506,847	32,308,450	90.99%	22,573,564	63.58%	20,108,032	89.08%	2	18,073	641,229	4.15	1
e30_SII	e30	SII	34	35	11.06.18	do26246	1	393	12.5	45,918,477	41,699,097	90.81%	27,273,171	59.39%	24,676,203	90.48%	2	21,036	926,212	4.45	1
e31_SI	e31	SI	35	35	11.06.18	do26247	1	262	39	40,486,472	36,709,938	90.67%	26,127,958	64.54%	23,096,416	88.40%	0	30,594	1,539,181	6.07	1
e31_SII	e31	SII	34	35	11.06.18	do26248	0	269	20.2	59,363,143	53,934,215	90.85%	39,535,843	66.60%	35,830,900	90.63%	0	10,671	496,829	3.11	0
e31_SIII	e31	SIII	33	35	11.06.18	do26249	0	334	37.1	33,880,646	30,756,782	90.78%	21,573,355	63.67%	19,623,065	90.96%	0	13,553	516,202	3.87	0
e32_SI	e32	SI	35	35	14.06.18	do26250	1	419	6.73	36,641,370	33,278,083	90.82%	22,217,580	60.64%	21,023,453	94.63%	3	23,125	1,178,262	4.81	1
e32_SII	e32	SII	34	35	14.06.18	do26251	1	436	6.36	66,155,891	60,239,521	91.06%	36,863,084	55.72%	35,286,664	95.72%	3	35,831	2,809,175	5.23	1
e32_SIII	e32	SIII	33	35	14.06.18	do26252	1	500	6.92	107,251,325	97,546,856	90.95%	52,103,795	48.58%	51,368,604	98.59%	4	25,444	2,494,536	3.80	1
e33_SI	e33	SI	35	35	14.06.18	do26253	1	424	10.76	33,566,554	30,537,273	90.98%	19,300,878	57.50%	17,850,388	92.48%	3	17,310	634,722	4.65	1
e33_SII	e33	SII	34	35	14.06.18	do26254	0	355	5.82	123,644,401	112,271,797	90.80%	98,363,427	79.55%	90,080,165	91.58%	0	6,807	449,023	1.39	0
e33_SIII	e33	SIII	33	35	14.06.18	do26255	0	519	7.78	37,302,329	34,000,527	91.15%	18,250,353	48.93%	17,779,911	97.42%	0	2,439	71,493	2.56	0

Table S3 | Quality control statistics from the ATAC-seq libraries, related to STAR Methods.
A third of the samples failed QC (in red). The 'unique' column corresponds to the number of fragments retained after removing PCR duplicates. The 'insertSizeDist', 'numberPeaks', 'readsInPeaks' and 'TSSenrichment' columns correspond to the metrics used to determine if

Data S1: microscopy images of all embryos used in this study, related to STAR methods. Embryo number corresponds to the annotation in Tables S1-3.

Stage: 8 somite pairs

e5	e6
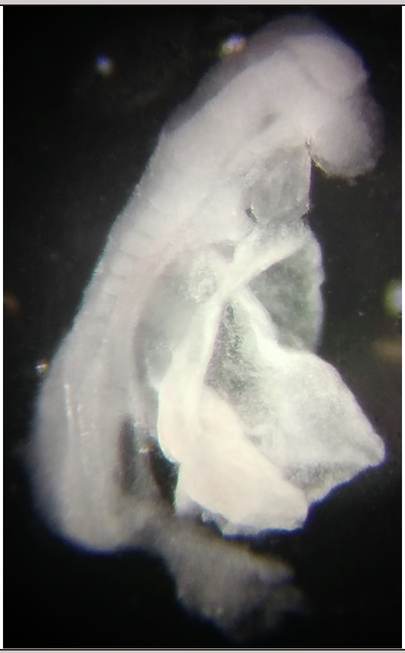	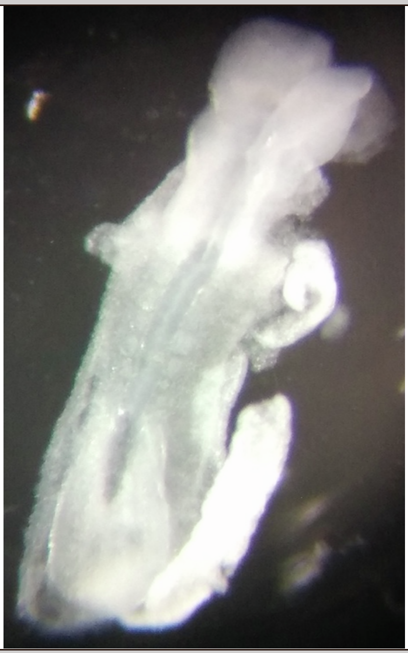
**e16**	**e26**
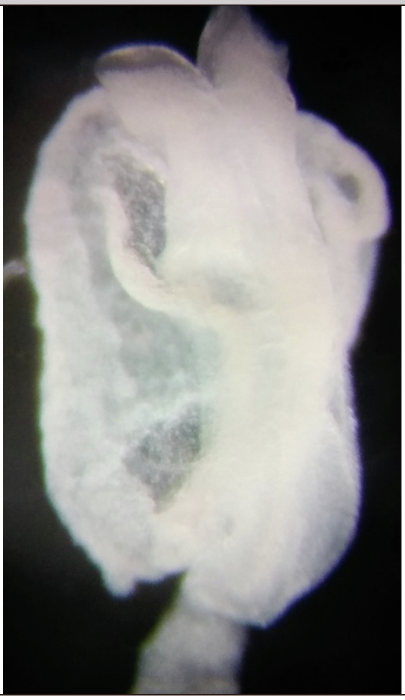	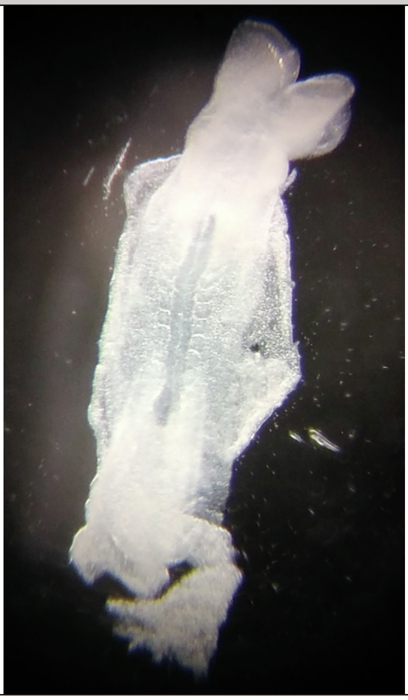

Stage: 18 somite pairs

e9	e14
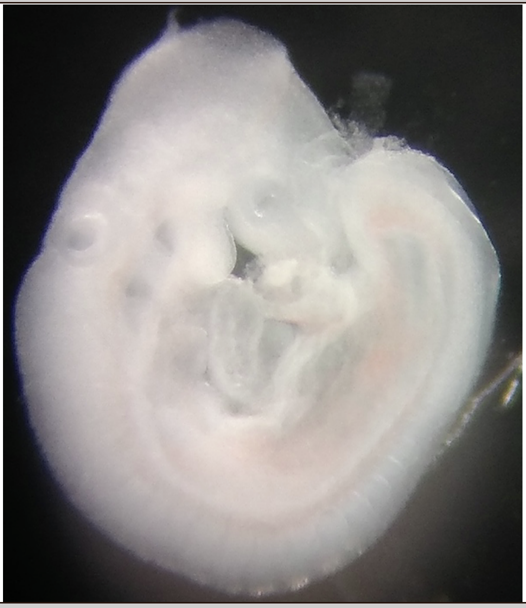	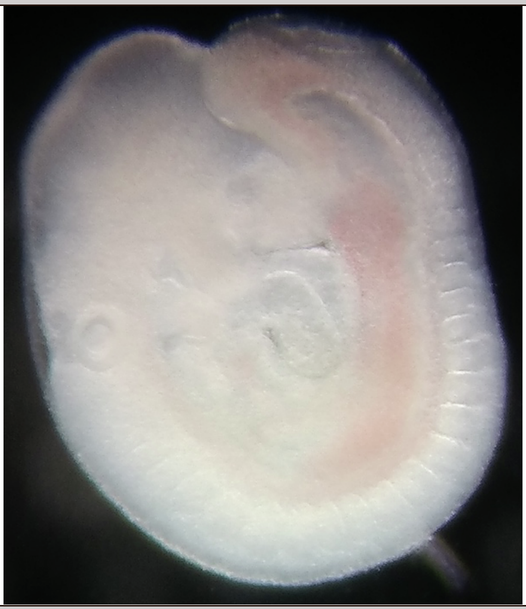
**e15**	**e27**
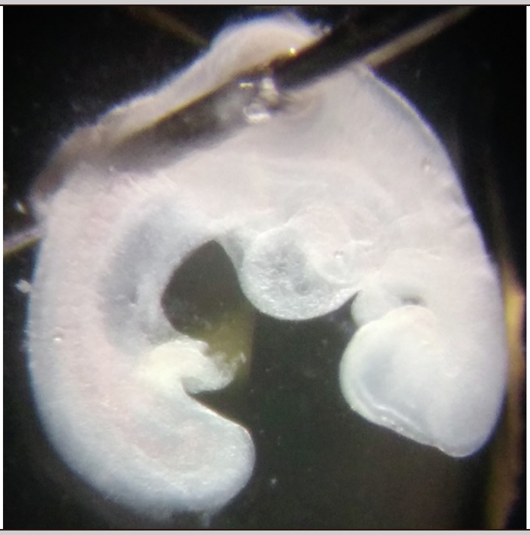	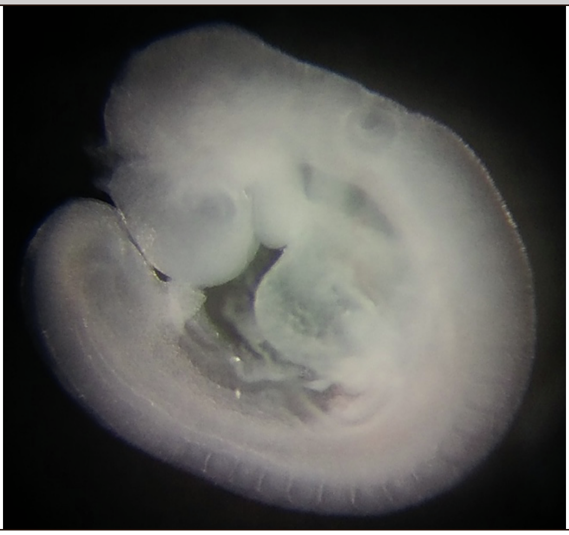
**e28**	
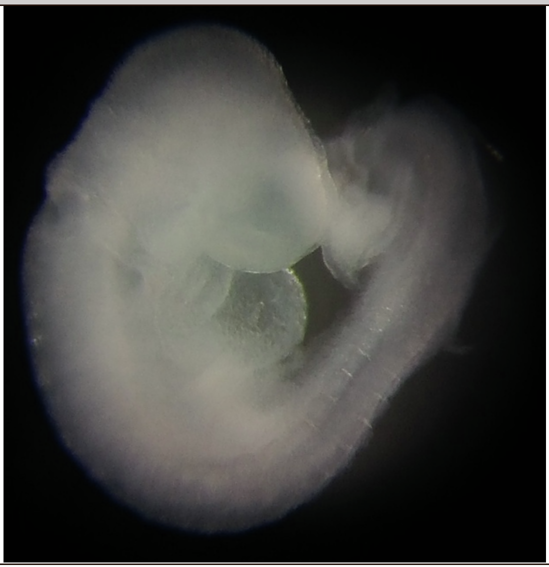	

Stage: 21 somite pairs

e1	e2
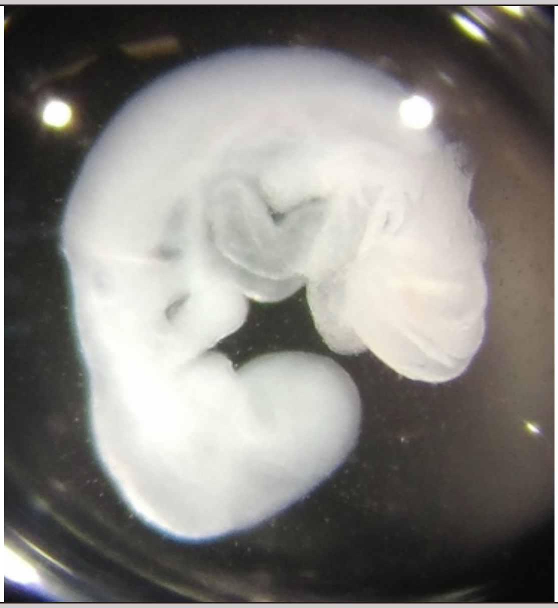	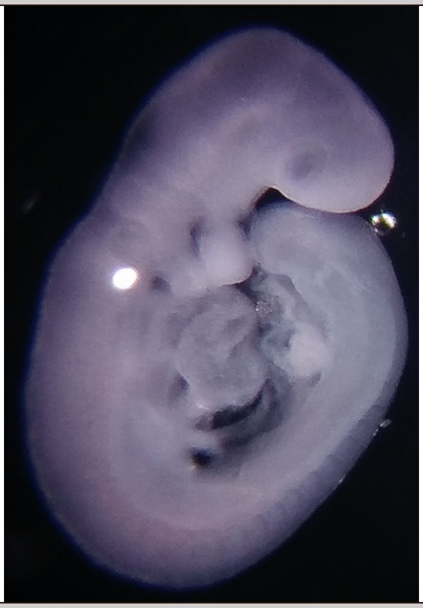
**e3**	**e13**
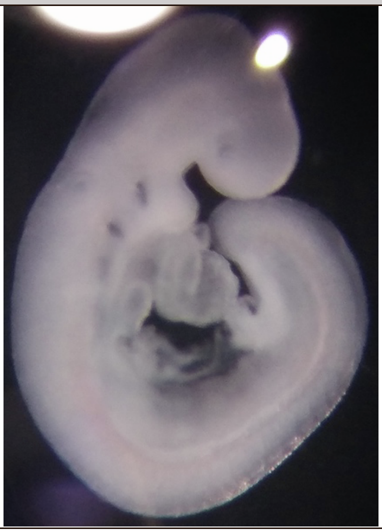	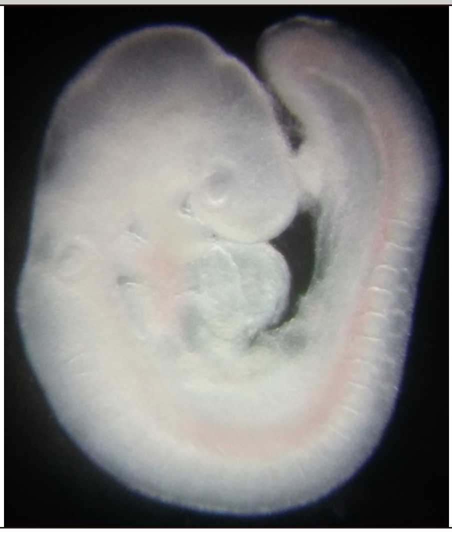

Stage: 25 somite pairs

e19	e20
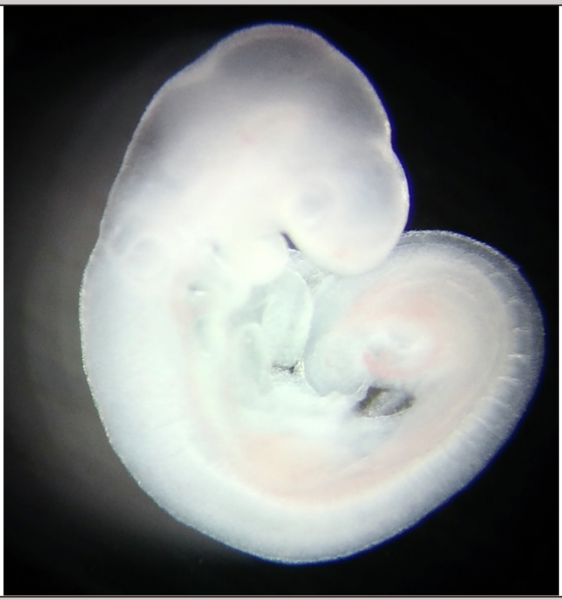	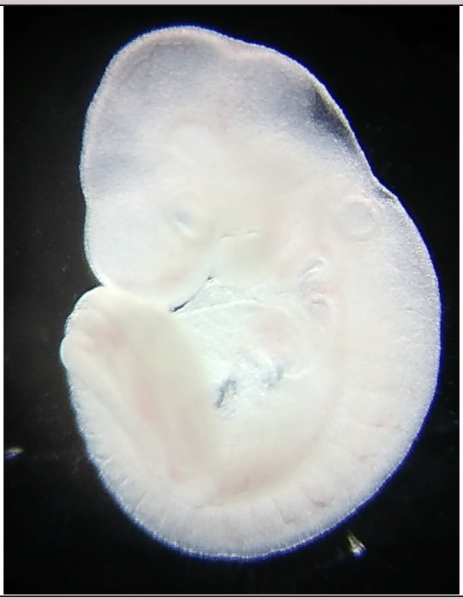
**e21**	**e22**
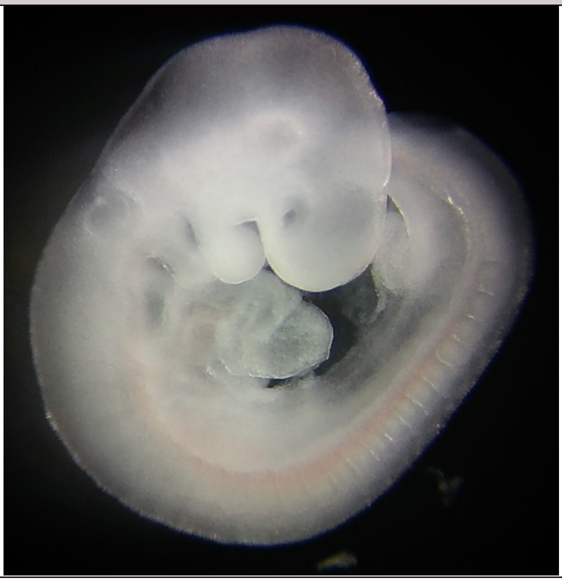	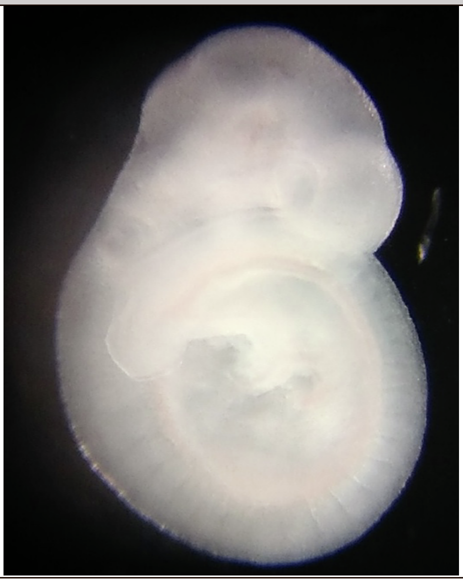

Stage: 27 somite pairs

e23	e24
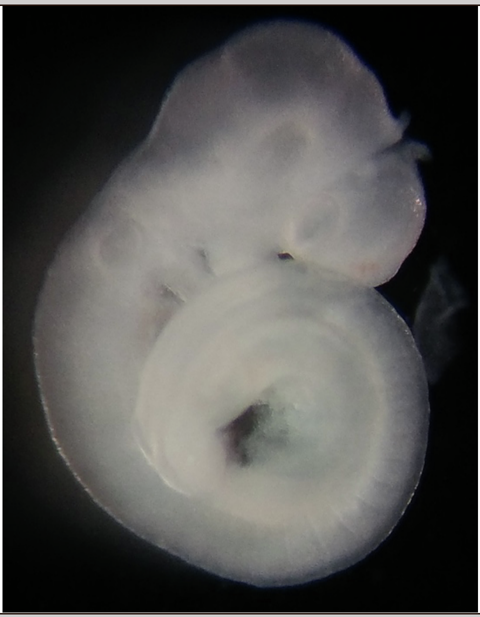	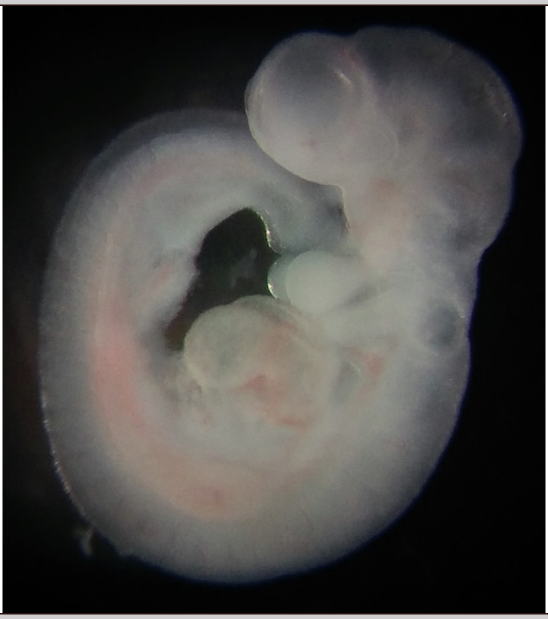
**e25**	**e34**
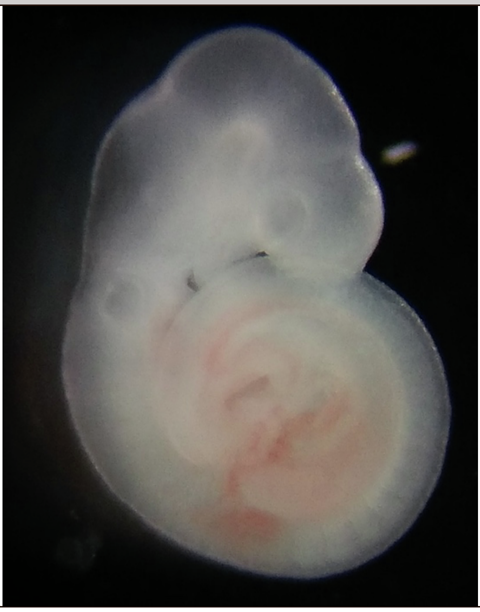	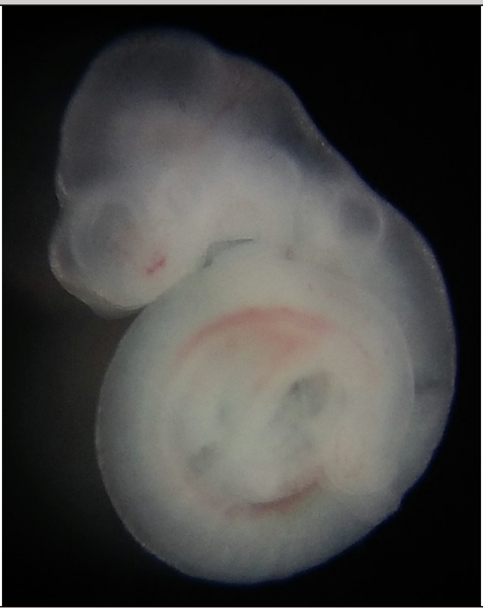

Stage: 35 somite pairs

e17	e29
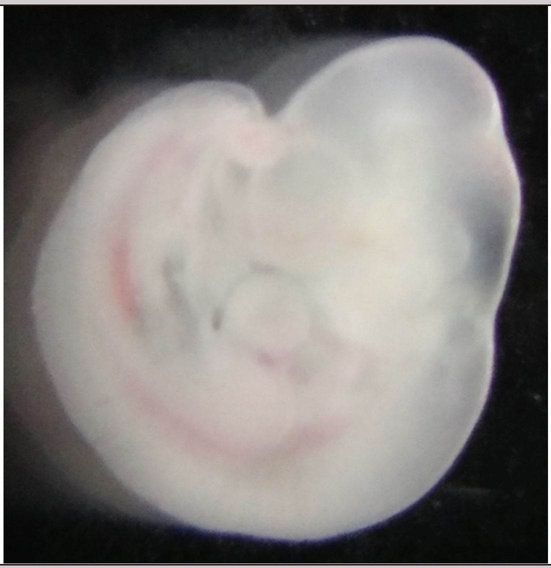	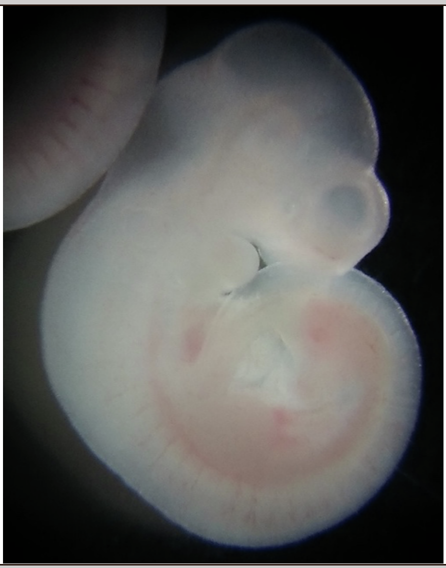
**e30**	**e31**
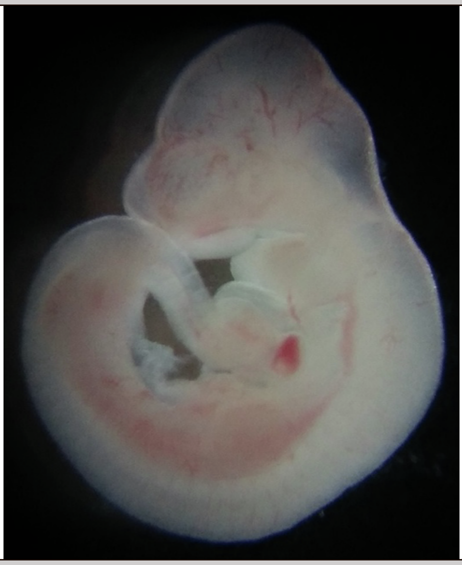	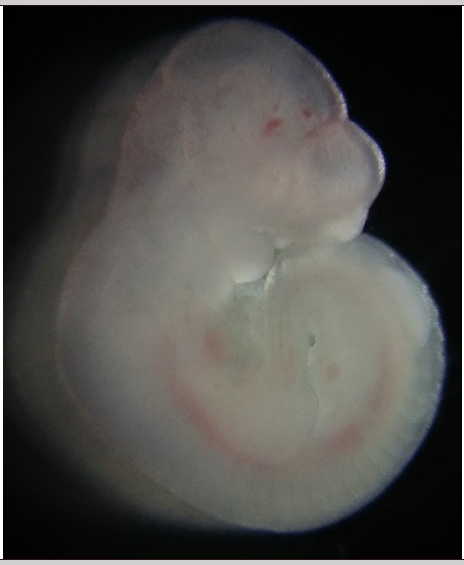
**e32**	**e33**
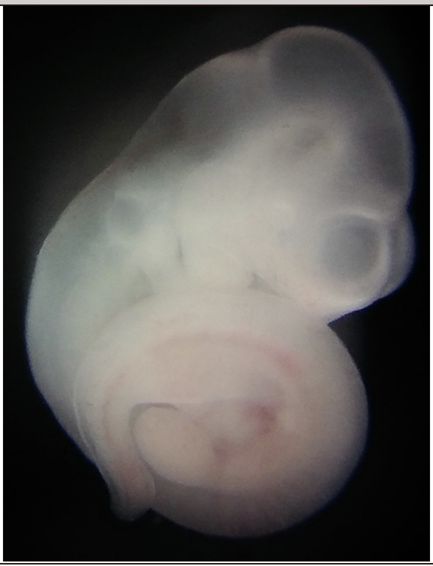	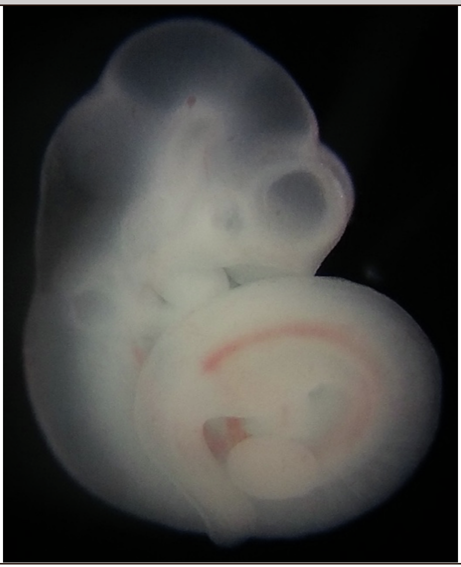

Example of dissected somites

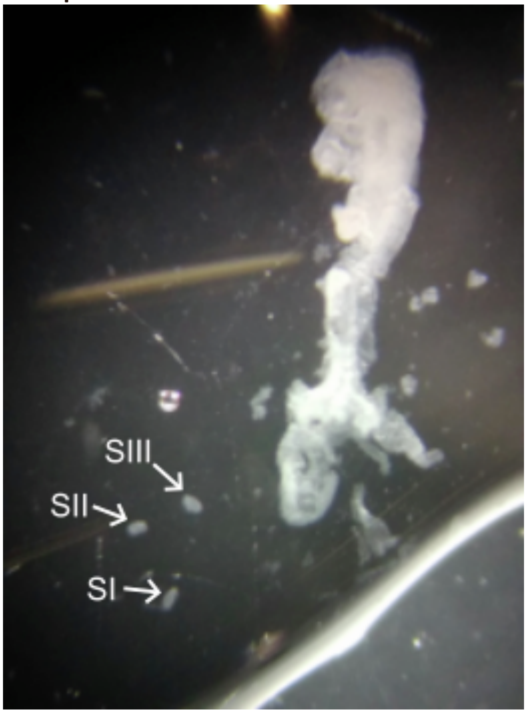

Supplement: Document S2. Article plus supplemental information [file mmc2.pdf]
